# Implications of stress-induced gene expression for hematopoietic stem cell aging studies

**DOI:** 10.1038/s43587-023-00558-z

**Published:** 2024-01-16

**Authors:** Anna Konturek-Ciesla, Rasmus Olofzon, Shabnam Kharazi, David Bryder

**Affiliations:** Division of Molecular Hematology, Lund Stem Cell Center, Institution for Laboratory Medicine, Lund University, Lund, Sweden

**Keywords:** Ageing, Gene expression analysis

## Abstract

A decline in hematopoietic stem cell (HSC) function is believed to underlie hematological shortcomings with age; however, a comprehensive molecular understanding of these changes is currently lacking. Here we provide evidence that a transcriptional signature reported in several previous studies on HSC aging is linked to stress-induced changes in gene expression rather than aging. Our findings have strong implications for the design and interpretation of HSC aging studies.

## Abstract (web summary)

This study reveals that many previously reported changes in gene expression in aging hematopoietic stem cells (HSCs) may be linked to extraction stress rather than aging itself and has implications for future HSC aging research.

## Main

Lifelong blood cell production emanates from bone marrow (BM) hematopoietic stem cells (HSCs). During aging, the hematopoietic output from HSCs declines^1^. To reveal the molecular mechanisms for this, several studies conducted genome-wide RNA expression profiling of young and aged candidate HSCs (cHSCs). Recently, a meta-analysis of previous data revealed a core aging signature (AS) of the most consistent gene expression changes, while highlighting discrepancies among different studies and datasets^2^.

In HSC research, cHSCs are typically extracted from the BM using fluorescence-activated cell sorting (FACS) and such procedures take time because of the scarcity of HSCs. Also, when isolating HSCs and other hematopoietic stem and progenitor cells (HSPCs) using dye uptake and exclusion techniques, cells are exposed to elevations in temperature before FACS. Limited information is available on how different experimental procedures might affect the molecular profiles of HSCs.

In this study, by reanalyzing transcriptomic HSC aging data from multiple previous studies, we identified a stress-associated signature that was uncoupled from aging. We found that some cell isolation procedures could evoke this response strongly in primary HSPCs. We discuss its implications for previous interpretations on HSC aging.

**Fig. 1:**
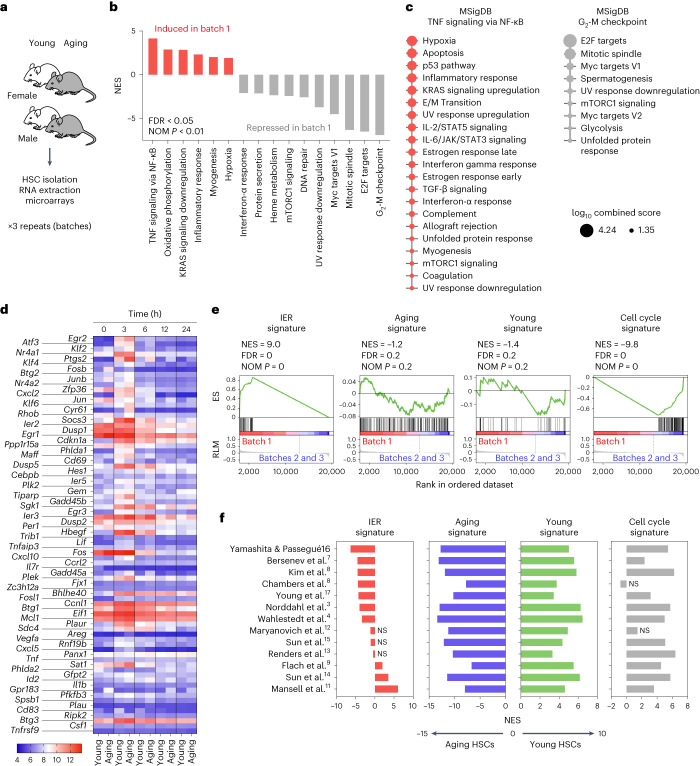
Identification of a batch-associated transcriptional signature that is unrelated to HSC aging. **a**, Experimental design. RNA was extracted from purified cHSCs (lineage-SCA-1^+^cKIT^+^CD150^+^CD48^−^) from cohorts of young (2–4 months) and aged (21–24 months) mice of both sexes and processed for gene expression analysis using Affymetrix 430 2.0 arrays. The experiment involved three separate batches (*n* = 4,000–10,000 per cHSC/group in each replicate). Data are from Norddahl et al.^3^ and Wahlestedt et al.^4^. **b**, GSEA for MSigDB Hallmark 2020 pathways that were associated with genes induced and repressed in batch 1. Shown are pathways with an FDR < 0.05 and nominal *P* < 0.01 (NOM *P*). **c**, MSigDB Hallmark 2020 pathway analysis of leading-edge genes extracted from the TNF signaling via NF-κB and G_2_-M checkpoint-associated gene sets. Shown are pathways with an adjusted *P* < 0.05 (one-sided Fisher’s exact test with Benjamini–Hochberg correction). The size of the dots is proportional to the Enrichr log_10_ combined scores, which is a combined metric of *P* values and ORs (computed using the Enrichr software). **d**, Heatmap depicting the time course gene expression of the 78 IER genes associated with batch 1 in freshly isolated and in vitro-cultured HSCs. Data are from Beerman et al.^33^. **e**, GSEA signature enrichment plots for the IER, age-induced, age-repressed and cell cycle-related genes in batch 1 cHSCs compared to cHSCs from batches 2 and 3. **f**, GSEA results for the IER, age-induced, age-repressed and cell cycle-related genes in young and aged cHSCs from the indicated datasets. Unless indicated by NS (not significant), the bars depict the normalized enrichment scores (NES) with an FDR < 0.05 and NOM *P* < 0.01. NES > 0 indicates signature enrichment in young cHSCs, while NES < 0 indicates enrichment in aged cHSCs. **b**,**e**,**f**, The FDR method was applied to correct for multiple hypotheses testing. **b**,**c**,**f**, Detailed statistical information is provided in Supplementary Table 2. RLM, ranked list metric. Source data

**Extended Data Fig. 1:**
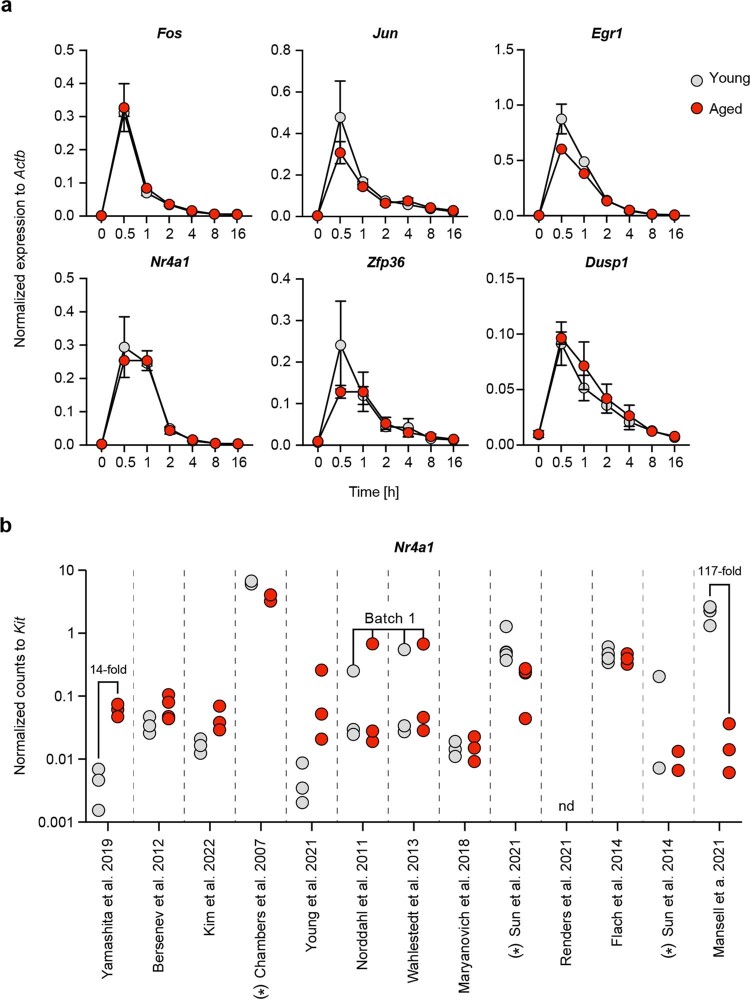
Validation of the IER signature. **a**) Time course expression of selected IERsig genes in *ex vivo* cultured HSPCs from young (2 months) and aged (20 months) mice. Error bars denote mean ± SEM (n = 2 mice/group analyzed in two independent experiments). Related to Fig. 1d. **b**) Expression of *Nr4a1* in young and aged cHSCs in the indicated datasets. The *Nr4a1* read counts were normalized to *Kit*. The asterisks (*) mark datasets that were generated using cell isolation procedures that involve incubations at 37 ºC (see also Supplementary Table 2). Fold differences in *Nr4a1* expression in young and aged cHSCs in Yamashita et al.^16^ and Mansell et al.^11^ are indicated above each comparison. Samples derived from batch 1 in Norddahl et al.^3^ and Wahlestedt et al.^4^ are also indicated. nd, not detected. Source data

Two of our previous microarray datasets were part of a meta-analysis on HSC aging^2^ and correlated well with the AS. However, while published separately as one for female and one for male cells^3,4^, they were generated simultaneously by isolating cHSCs in three batches, each with samples from young and aged male and female cells (Fig. 1a). This setup allowed us to directly assess potential batch effects. When comparing batch 1 to the other two batches for Molecular Signatures Database (MSigDB) Hallmark gene sets^5^, we observed that genes expressed higher in batch 1 included several immediate early response (IER) genes (Supplementary Table 1) that associated particularly to ‘tumor necrosis factor (TNF) signaling via nuclear factor kappa-light-chain-enhancer of activated B cells (NF-κB)’, with downregulated genes relating strongly to ‘G_2_-M checkpoint‘ (Fig. 1b and Supplementary Table 2). We defined an IER signature (IERsig) based on these upregulated genes (Supplementary Table 1). The IER genes were also positively associated with many other gene sets coupled to stress and negatively to cell cycle pathways (Fig. 1c and Supplementary Table 2). It is unknown to us why the samples of our first batch associated more with these expression signatures. Regardless, we next assessed the IERsig after brief culture of young and aged cHSCs to evaluate whether aging impacted on its induction. The IERsig genes *Fos* and *Jun* presented with high expression already after 0.5 h of culture, with other assessed IER genes peaking at 1–3 h and declining thereafter (Fig. 1d and Extended Data Fig. 1a). This agrees with *Fos* and *Jun* being primary IER genes that decline rapidly after their induction^6^. Notably, the induction of the IERsig was independent of age (Fig. 1d and Extended Data Fig. 1a).

Using gene set enrichment analysis (GSEA) for the IERsig and cell cycle gene sets from our batch analysis (Fig. 1b), and AS and young signature sets from Flohr Svendsen et al.^2^, we assessed genome-wide transcription in 13 reported datasets on murine HSC aging^3,4,7–17^ (Supplementary Tables 2 and 3). These gene sets were benchmarked by comparing batch 1 to the other two batches (Fig. 1e). In all investigated studies, aged cHSCs displayed a strong AS, with the young signature associating with the young samples. Furthermore, in general, cell cycle genes were associated with young cHSCs. By contrast, the IERsig varied significantly between the datasets, being enriched in young or old samples, or showing no age-dependent enrichment (Fig. 1f, Extended Data Fig. 1b and Supplementary Table 2).

The literature presents a complex landscape when examining transcriptional features in HSCs associated with aging. One perspective posits that aging HSCs share transcriptional characteristics similar to those triggered by TNF^16^. However, this viewpoint may be influenced by the particular dataset generated, which showed a high correlation of the aged HSCs with the IERsig (Fig. 1f). Another line of research suggested that young HSCs may exhibit faster gene transcription than aging ones^11^, but a contrasting study posited an inverse relationship, indicating that transcription rates could actually be higher in aging HSCs^2^. The faster transcription observed in young HSCs in the former study could potentially be attributed to a strong induction of the IERsig in young cells in the particular dataset generated (Fig. 1f). The complexity deepens with work proposing reduced transforming growth factor-β (TGFβ) signaling in aging cHSCs^14^, which was not observed in a later meta-analysis^2^. The connection between the IERsig and TGFβ (Fig. 1c) and the specific genes showcased as downregulated with age in Sun et al.^14^, which represent prominent IER genes, is noteworthy. Finally, it is crucial to acknowledge that there are datasets that exhibit no discernible link with the IERsig in HSCs from either age group (Fig. 1f). Therefore, although RNA profiling studies have often detected the IERsig when examining murine HSC aging, its relationship to age is inconsistent. This variability urges cautious interpretation of preceding findings involving these genes.

**Fig. 2:**
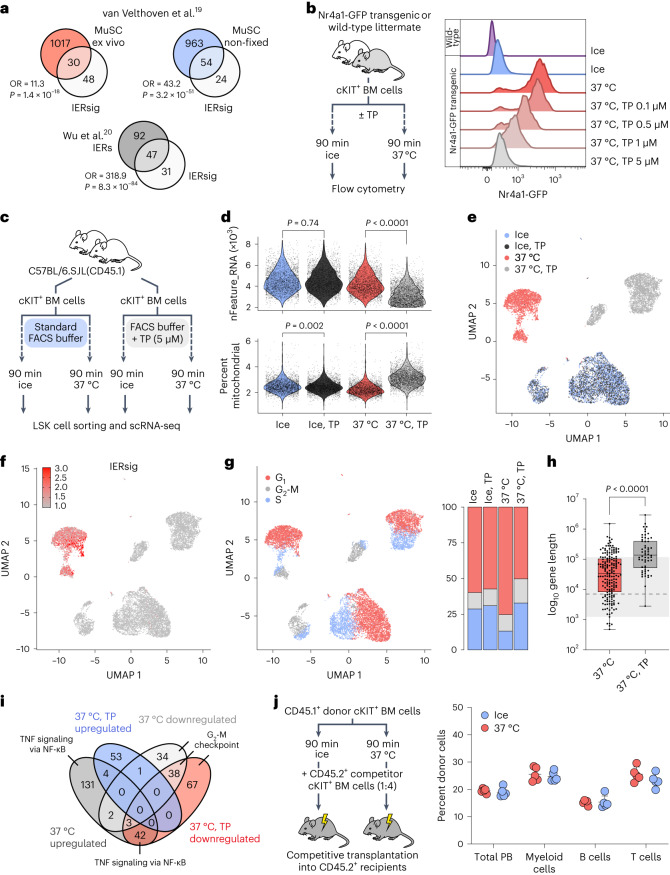
The effect of cell isolation procedures on induction of the IER signature. **a**, Top left, IERsig genes compared to differentially increased nascent transcripts (FDR < 0.1, fold change > 2) during muscle stem cell (MuSC) isolation (red circle). Top right, Overlap of IERsig genes with differentially upregulated genes in untreated versus in vivo-fixed MuSCs (FDR < 0.1, fold change > 2, blue circle). Data were derived from van Velthoven et al.^19^ Bottom, IERsig genes compared to IER genes identified by Wu et al.^20^ (gray circle). Each comparison displays the OR and *P* values (two-sided Fisher’s exact test). **b**, Left, experimental design. Right, representative histograms depicting the Nr4a1-GFP signal in HSPCs incubated on ice or at 37 °C with increasing concentrations of TP (*n* = 3–4 mice per genotype analyzed in three independent experiments). **c**, Experimental design to evaluate the IER in lineage-SCA-1^+^cKIT^+^ (LSK) HSPCs using scRNA-seq. **d**, Feature counts (ice ± TP, *P* = 0.074; 37 °C ± TP, *P* < 0.0001) and mitochondrial gene content (ice ± TP, *P* = 0.002; 37 °C ± TP, *P* < 0.0001) detected per cell (two-sided Mann–Whitney *U*-test). **e**, UMAP embedding of 12,556 LSK cells color-coded based on sample assignments (37 °C, *n* = 2,873 cells; 37 °C TP, *n* = 3,580 cells; ice, *n* = 3,048 cells; ice, TP *n* = 3,065 cells). **f**, UMAP embedding of LSK cells with aggregated IERsig expression depicted in red. **g**, Left, UMAP embedding of LSK cells color-coded according to cell cycle phase. Right, bar graphs depicting cell cycle phase distributions across samples. **h**, Distribution of gene lengths for genes upregulated in HSPCs incubated at 37 °C ± TP compared to cells kept on ice (*n* = 183 and *n* = 58 genes respectively, analyzed in one experiment). The box plots span the 25th to 75th centiles. The whiskers and center line indicate the minimum, maximum and median length. The background box represents the range of lengths for IERsig genes, with the dashed line indicating the median. *P* < 0.0001 (two-sided Mann–Whitney *U*-test). **i**, DEGs in HSPCs incubated at 37 °C ± TP compared to cells kept on ice. The MSigDB Hallmark 2020 pathways with the highest Enrichr combined score (adjusted *P* < 0.05, one-sided Fisher’s exact test with Benjamini–Hochberg correction) for the indicated groups of genes are shown. **j**, Left, experimental design. Right, donor-derived chimerism in the indicated peripheral blood (PB) lineages 16 weeks after transplantation (*n* = 5 mice per group). The error bars denote the mean ± s.d. Source data

**Extended Data Fig. 2:**
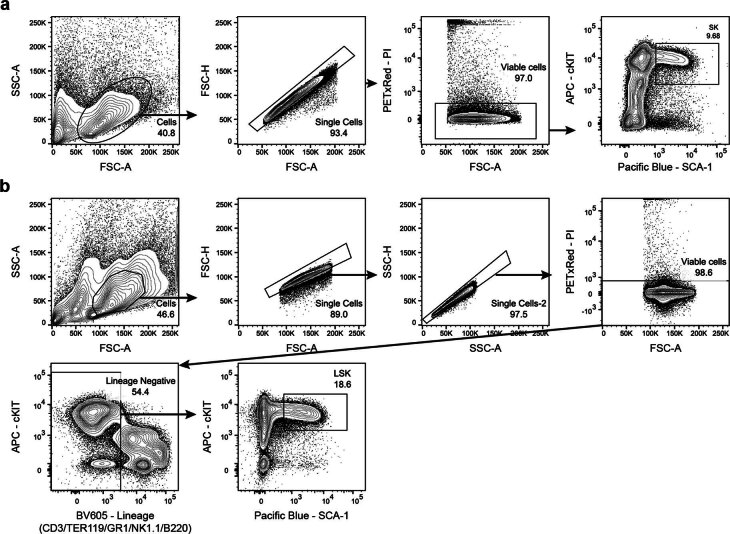
The gating strategy for flow cytometry analysis and FACS sorting. **a**) The gating strategy for the analysis of Nr4a1-GFP signal in bone marrow SCA-1+cKIT+ HSPCs. Related to Fig. 2b. **b**) The gating strategy for bone marrow LSK cell sorting for single cell RNA sequencing experiment. Related to Fig. 2c.

The IERsig has been highlighted as an artifact during cell preparations outside hematopoiesis, which often require elevations in temperature^18^. Indeed, by comparing the IERsig to stress-associated genes identified in previous studies on muscle or neuronal cells^19,20^, we observed a significant overlap (Fig. 2a). We next used a green fluorescent protein (GFP)-reporter mouse line for *Nr4a1* (ref. ^21^), a prominent IER gene (Extended Data Fig. 1), and compared cKIT^+^ Nr4a1-GFP BM cells kept on ice with those incubated at 37 °C for 90 min (Fig. 2b and Extended Data Fig. 2a). We also explored whether the IERsig could be mitigated with the transcriptional inhibitor triptolide (TP). Incubation at 37 °C led to a pronounced induction of GFP, which was blocked by TP in a dose-dependent manner (Fig. 2b). Hence, while cHSCs can be isolated with less induction of the IERsig, procedures that involve a rise in temperature lead to its direct induction.

To detail the IERsig further, we assessed the effects of temperature and TP using single-cell RNA sequencing (scRNA-seq) of HSPCs isolated from young mice (Fig. 2c and Extended Data Fig. 2b). We noted a significant reduction in RNA feature counts and a small yet steady elevation in mitochondrial gene content when incubating at 37 °C with TP, implying a decline in overall cell health after TP treatment (Fig. 2d). Using uniform manifold approximation and projection (UMAP), we distinguished cell clusters based on their treatment (Fig. 2e). Cells kept on ice formed a cluster regardless of TP treatment, while the samples incubated at 37 °C with or without TP formed separate clusters. Analysis of the IERsig revealed a potent and fairly uniform activation after incubation at 37 °C, which TP prevented. No noticeable impact from TP on the IERsig was observed when cells were kept on ice (Fig. 2f).

**Extended Data Fig. 3:**
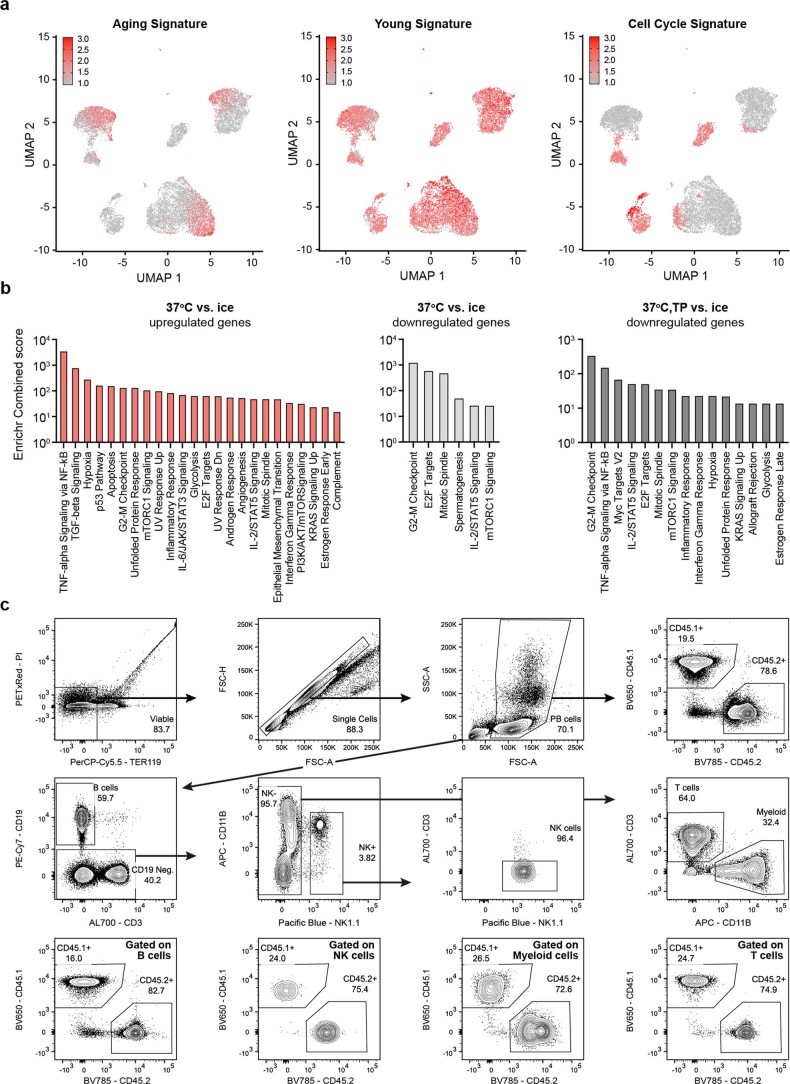
The effect of cell isolation procedures on induction of the IER signature. **a**) UMAP embeddings of LSK cells with the aggregated expression of the age-induced, age-repressed and cell cycle-related genes depicted in red. Related to Fig. 2f. **b**) The MSigDB Hallmark 2020 pathways that associate with genes induced and repressed in HSPCs incubated at 37 °C ± TP compared to cells kept on ice. Shown are Enrichr combined scores (computed by Enrichr software) for pathways with an adjusted p-value < 0.05 (one-sided Fisher’s exact test with Benjamini-Hochberg correction). No significant pathways were associated with genes upregulated in HSPCs incubated at 37 °C + TP. Detailed statistical information is provided in Supplementary Table 2. Related to Fig. 2i. **c**) Representative flow cytometry analysis of donor-derived chimerism in indicated peripheral blood lineages. Related to Fig. 2j. Source data

The projection of additional signatures identified in our batch analysis (Fig. 1b) on the scRNA-seq data revealed a homogenous distribution of the young-associated signature across samples (Extended Data Fig. 3a). The AS was associated with specific regions for each of the three clusters, but with no association as for treatment, and was inversely correlated to the cell cycle signature (Extended Data Fig. 3a). In agreement with the stress-induced downregulation of genes associated with the cell cycle pathways (Fig. 1b), further analysis revealed a pronounced decrease in the proportion of actively cycling cells after incubation at 37 °C (Fig. 2g).

Exploring the differentially expressed genes (DEGs) in cells incubated at 37 °C ± TP (Supplementary Table 4) revealed that the genes upregulated in the absence of TP exhibited remarkably shorter lengths compared to those induced after TP treatment (Fig. 2h). In addition, by intersecting these DEGs, we identified the ‘TNF signaling via NF-κB’ as the most prominent pathway upregulated at 37 °C and that could be mitigated by transcriptional inhibition (Fig. 2i). Consistent with our previous observations, genes downregulated at 37 °C associated strongly with cell cycle progression (Fig. 2i and Extended Data Fig. 3b).

Finally, to assess the impact of IERsig on the in vivo function of HSPCs, we competitively transplanted cKIT^+^ BM cells incubated on ice or at 37 °C for 90 min (Fig. 2j and Extended Data Fig. 3c). This revealed no functional consequences of IERsig induction on long-term HSC activity (Fig. 2j).

In this study, we highlighted variable activation of the IERsig across different research studies on HSC aging. Although some of these discrepancies may be due to variations in experimental design and methods across different studies (Supplementary Table 3), our findings underscore that HSPC exposure to stress triggers a swift induction of the IERsig, which is independent of the cells’ age. Consequently, variations in handling of cells during isolation and the time elapsed until RNA extraction are probable factors contributing to the induction of the IERsig in primary HSPCs.

**Extended Data Fig. 4:**
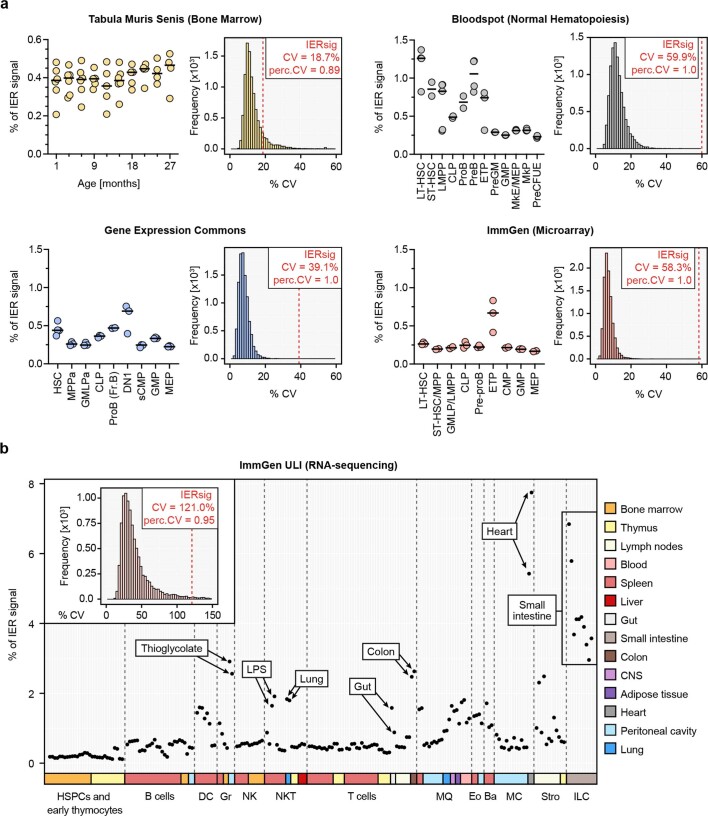
Expression of the IER signature in indicated datasets. **a**) Dot plots depicting the cumulative expression of IERsig genes in BM samples across various age groups and different HSPC subsets in indicated datasets. Data points indicate individual mice and bars denote median. Histograms display the distribution of bootstrap coefficient of variation (CV) and the CV of the IERsig in the respective datasets. Data derive from the Tabula Muris Senis^25^, Immunological Genome Project (ImmGen, microarray)^23^, Bloodspot Mouse Normal Hematopoietic System^22^ and Gene Expression Commons^24^ datasets. **b**) Aggregated expression of IERsig genes in distinct hematopoietic cell populations isolated from various organs. Samples derived from mice exposed to inflammatory stimuli (Thioglycolate and lipopolysaccharide, LPS), or from selected organs that require enzymatic treatment are indicated. The histogram in the top-left corner represents the distribution of bootstrap coefficient of variation (CV) and the CV of the IERsig. Data derive from Immunological Genome Project (ImmGen ULI RNA sequencing)^26^. Ba, basophils; DC, dendritic cells; Eo, eosinophils; Gr, granulocytes; ILC, innate lymphoid cells; NK, natural killer cells; NKT, natural killer T cells; MC, mast cells; MQ, macrophages; Stro, stromal cells Source data

We present our findings primarily in the context of HSC aging, but we have also observed that samples from the reference sets on defined HSPCs, including BloodSpot^22^, Immunological Genome Project (ImmGen)^23^, Gene Expression Commons^24^ and data in the Tabula Muris Senis project^25^, all associate with the IERsig to various degrees (Extended Data Fig. 4a). Furthermore, in a recent extensive large-scale RNA-seq dataset on murine immune cells (ImmGen ULI)^26^, we observed that while a high IERsig associated with samples exposed to inflammatory stimuli (including thioglycolate and lipopolysaccharide), probably due to an elevated immune activation, a markedly upregulated IERsig was also consistent in samples extracted from organs that require enzymatic treatment at elevated temperatures (Extended Data Fig. 4b). Crucially, the IERsig in all these evaluated datasets was more likely to show variability than random gene sets (Extended Data Fig. 4). Hence, while some transcriptional features, in this study related to HSC aging, can still be extracted from cells with a simultaneous IERsig, this requires for the most part previous knowledge. Also, the identity of all the genes comprising the IER in HSPCs and their relationship to subsequent waves of transcription are unclear. For instance, given the central role of quiescence in HSC biology, the correlation between induction of the IERsig with reductions in cell cycle-associated genes^18,27^ is noteworthy.

**Extended Data Fig. 5:**
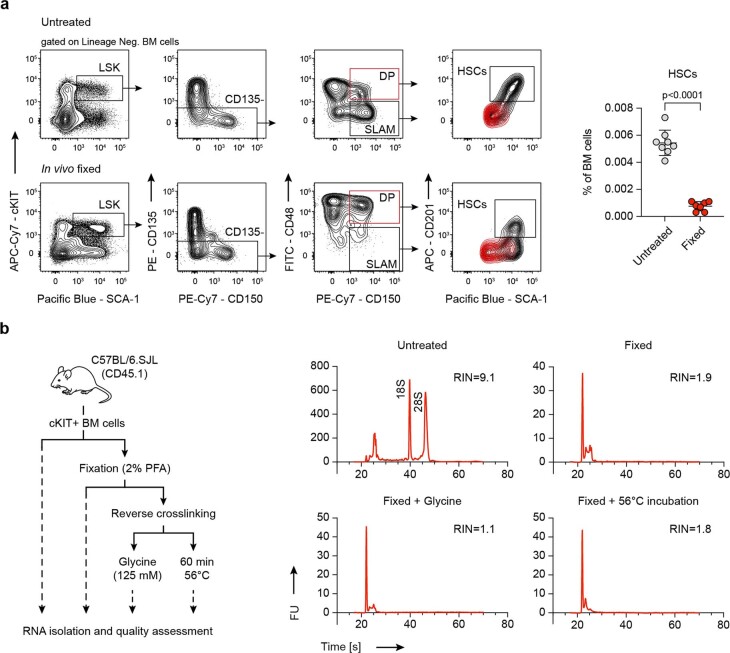
The effect of fixation on HSPC phenotypes and RNA quality. **a**) Evaluation of HSC phenotypes (*left*) and frequencies (*right*) in C57BL/6 mice following *in vivo* fixation with paraformaldehyde (n = 8). Untreated mice served as controls (n = 7). LSK CD135-CD48+CD150+ population (DP, red) was used as internal negative control to set the gate for CD201. Analyses were performed in three independent experiments. Error bars denote mean ± SD, p = 1.4 × 10^−8^ (two-sided Student’s *t*-test). **b**) Analysis of RNA recovery and integrity following isolation from untreated and *ex vivo* fixed HSPCs. Experimental strategy (*left*) and electropherograms representing RNA profiles (*right*). RNA was 10x diluted prior to analysis. FU, arbitrary fluorescence units Source data

While methods have been suggested to counteract the expression of IER genes caused by preparation, such as adding transcription inhibitors to isolation buffers^20^, we found that this can have its own drawbacks by affecting RNA quality (Fig. 2d) and the induction of other genes with specific attributes (Fig. 2h,i). Likewise, and in agreement with what has previously been observed for MuSCs^19^, in vivo cell fixation before isolation^18,19^ led to changes in cell surface epitopes and substantial losses of recoverable cells (Extended Data Fig. 5a), with fixation also leading to poor RNA yield and integrity (Extended Data Fig. 5b). Instead, as a currently more effective approach for evaluating gene expression in primary HSPCs, we advocate for careful maintenance of primary HSPCs on ice during isolation, coupled with batch processing and a subsequent monitoring of the IERsig in the ensuing gene expression profiles.

## Methods

### Mice

Young (2–6 months) and aged (20 months) C57BL/6NTac (strain no. B6, Taconic Bioscience), C57BL/6.SJL (bred in house) and Nr4a1-GFP (strain no. 016617, The Jackson Laboratory) male and female mice were used. Mice were housed in a controlled environment with 12-h light–dark cycles with chow and water provided ad libitum. All animal procedures were approved by the Lund University Ethics Committee (no. 16468-20).

### Reagents

All commercially available reagents used in this study are listed in Supplementary Table 5.

### Flow cytometry analyses and cell sorting

BM cells were isolated from the tibia, femur and pelvis into ice-cold FACS buffer (2% FCS/PBS) with or without 5 µM TP (Tocris)^28^ and filtered through 70-µm cell strainers. Cells were cKIT-enriched by anti-cKIT-APC staining, followed by incubation with anti-APC MicroBeads and magnetic separation on LS columns (Miltenyi Biotec). Aliquots of cells were resuspended in culture medium (DMEM with high glucose, 10 mM HEPES, 2% FCS) ± TP (0.1, 0.5, 1 and 5 µM) and incubated on ice or at 37 °C for 90 min. After incubation, cells were stained with biotinylated antibodies against B220, Gr-1, TER119, CD3, NK1.1 and Sca-1 Pacific Blue (Sony Biotechnology) for 30 min on ice in the dark. For scRNA-seq, cells were additionally stained with oligo-conjugated hashing (HTO) antibodies (TotalSeq-A0301, -A0302, -A0303, and -A0304, BioLegend). Secondary staining was performed with streptavidin-Brilliant Violet 605 (Sony Biotechnology). After staining, LSK HPSCs were FACS-purified on a BD FACS Aria III instrument (BD Biosciences) and subjected to single-cell profiling (10x Genomics) or analyzed on an LSRFortessa X20 analyzer with the FACS Diva software v.9.0 (BD Biosciences). For PB analysis using flow cytometry, blood samples were sedimented with 1% Dextran T500 (Sigma-Aldrich) for 30 min at 37 °C; the remaining erythrocytes were lysed using ammonium chloride solution (STEMCELL Technologies) for 3 min at room temperature. Cells were stained with CD19-PE-Cy7, TER119-PerCP-Cy5.5, CD11B-APC, NK1.1-Pacific Blue, CD3-Alexa Fluor 700, CD45.1-Brilliant Violet 650 and CD45.2-Brilliant Violet 785 (Sony Biotechnology). For the analysis of in vivo-fixed HSPCs, unfractionated BM cells were stained with PE-Cy5-conjugated B220, Gr-1, TER119, CD3, NK1.1, Sca-1 Pacific Blue, CD48-FITC, CD150-PE-Cy7, CD135-PE (Sony Biotechnology) and cKIT-APC-eFluor 780, CD201-APC (eBioscience) antibodies. Before analysis or sorting, cells were stained with propidium iodide (1:1,000, Invitrogen) to exclude dead cells. Data analysis was done using FlowJo v.10.5.3 (FlowJo LLC).

### Transplantation experiment

cKIT-enriched BM cells derived from C57BL/6.SJL-CD45.1 donor mice were isolated into ice-cold FACS buffer and incubated on ice or at 37 °C for 90 min. Competitor cKIT-enriched BM cells derived from C57BL/6N-CD45.2 mice were kept on ice throughout the whole experiment. After incubation, donor and competitor cells were mixed at a 1:4 ratio and transplanted into lethally irradiated (9.5 Gy) C57BL/6N-CD45.2 recipient mice via intravenous injections. Recipient mice received antibiotic prophylaxis (ciprofloxacin-supplemented water, 125 mg l^−1^, Krka) for 2 weeks beginning on the day of irradiation. Donor-derived reconstitution was monitored in PB using flow cytometry.

### Analysis of in vivo-fixed HSPCs

Young (2 months old) C57BL/6 female mice were anesthetized with ketamine (MDS Animal Health)-xylazine (Bayer Animal Health) (10% KX, 10 µl g^−1^); in vivo fixation was performed using transcardiac perfusion with PBS followed by 4% paraformaldehyde (PFA). BM cells were isolated from the tibia, femur and pelvis into ice-cold FACS buffer (2% FCS/PBS), filtered through 70-µm cell strainers and processed as described in the ‘Flow cytometry analyses and cell sorting’ section.

### Quantitative PCR with reverse transcription

cKIT-enriched BM cells were isolated from young (2 months) and aging (20 months) C57BL/6 male mice and incubated at 37 °C for up to 16 h in DMEM High Glucose (Gibco) supplemented with 10% FCS, 0.1 mM 2-mercaptoethanol (Invitrogen), 1× penicillin-streptomycin-glutamine (Invitrogen), 50 ng ml^−1^ stem cell factor, 10 ng ml^−1^ TPO and 10 ng ml^−1^ Flt3L (all from PeproTech). After incubation, RNA was extracted using the Direct-zol RNA MicroPrep Kit with on-column DNase I treatment (Zymo Research). Reverse transcription was carried out using the qScript cDNA Supermix according to the manufacturer’s instruction (Quantabio). The resulting complementary DNA (cDNA) was used for quantitative PCR with reverse transcription (RT–qPCR) with SsoAdvanced SYBR Green Supermix (BioRad Laboratories) and the following primers: *Actb*: 5′-CCACAGCTGAGAGGGAAATC-3′ (forward), 5′-CTTCTCCAGGGAGGAAGAGG-3′ (reverse); *Dusp1:* 5′-ACCATCTGCCTTGCTTACCTC-3′ (forward), 5′-CTCCGCCTCTGCTTCACAAA-3′ (reverse); *Egr1:* 5′-CCTATGAGCACCTGACCACA-3′ (forward), 5′-GAAGCGGCCAGTATAGGTGA-3′ (reverse); *Fos:* 5′-ATGGGCTCTCCTGTCAACAC-3′ (forward), 5′-GCTGTAACCGTGGGGATAA-3′ (reverse); *Jun:* 5′-GGAAACGACCTTCTACGACGAT-3′ (forward), 5′-GGGTTACTGTAGCCGTAGGC-3′ (reverse); *Nr4a1:* 5′-TTGAGTTCGGCAAGCCTACC-3′ (forward), 5′-GTGTACCCGTCCATGAAGGTG-3′ (reverse); *Zfp36:* 5′-CCCTCACCTACTTCGCCTAC-3′ (forward), 5′-ACTTGTGGCAGAGTTCCGTTT-3′ (reverse). Analyses were performed in two independent experiments with *n* = 2 biological replicates per group.

### Analysis of RNA integrity from fixed HSPCs

cKIT-enriched BM cells were isolated from young (2 months old) C57BL/6.SJL female mice and split into four groups (approximately 900,000 cells per sample). Untreated cells were kept on ice throughout the whole procedure. Cells from remaining samples were spun down, resuspended in 2% PFA in PBS and incubated at room temperature for 15 min. Cells were spun down at 400*g* for 5 min after adding wash buffer (PBS supplemented with 1% BSA and 40 U ml^−1^ SUPERase•In, Invitrogen) and were subsequently resuspended in the wash buffer. For reverse crosslinking, cells were incubated either at room temperature for 5 min in wash buffer supplemented with 125 mM glycine (Serva) or at 56 °C for 60 min in wash buffer supplemented with 40 U ml^−1^ proteinase K (Roche), followed by incubation on ice for at least 5 min. RNA was isolated using the Direct-zol RNA MicroPrep Kit with on-column DNase I treatment (Zymo Research). RNA integrity was measured using the RNA 6000 Pico Kit and a 2100 Bioanalyzer instrument (Agilent Technologies).

### scRNA-seq and bioinformatics analysis

scRNA-seq was performed in a single batch. BM cells were isolated from a pool of young (2–3-month-old) C57BL.6/SJL female mice (*n* = 5), split into four groups and processed simultaneously. cDNA libraries were generated using the Chromium Next GEM Single Cell 3′ Reagent Kit v.3.1 (10x Genomics). Briefly, LSK cells (40,000 cells for each group) were sorted into ice-cold FACS buffer, resuspended at 1,250 cells per microliter in FACS buffer and 54,000 cells were loaded onto the Chromium Controller (10x Genomics). The cDNA sequencing library was generated according to the manufacturer’s instructions (10x Genomics). The HTO-derived sequencing library was prepared according to CITE-seq_and_Hashing_protocol_190213 (https://cite-seq.com/protocols). Final cDNA-derived and HTO-derived libraries were pooled and sequenced on an Illumina NovaSeq 6000 instrument using the S2 Reagent Kit v.1.5 (100 cycles). Preparation of libraries and sequencing were done at the Center for Translational Genomics at Lund University.

After sequencing libraries were processed using Cell Ranger v.7.0.0. The FASTQ files were aligned to the mouse reference genome (mm10) to create unique molecular identifier count tables of gene expression. HTO demultiplexing was performed with cellhashr (https://github.com/BimberLab/cellhashR). Quality control and downstream analyses were performed using Seurat^29^ v.4.1.1. The filtering thresholds used for cell barcode exclusion were as follows: a high number of mitochondrial transcripts (>6%); low-quality or empty droplets (the number of detected genes below 2,000 or above 9,000 and the number of detected transcripts below 5,000 or above 60,000); and the number of HTO transcripts below 20 or above 400. Motivated by the simultaneous processing of all cells and the near complete overlap between two of the sample groups (the two groups where cells were kept on ice; Fig. 2e), no additional sample integration was performed. Identification of highly variable genes, data normalization and dimensionality reduction were performed using Seurat^29^ with default parameters.

Cell cycle analysis was carried out using the CellCycleScoring function from Seurat. For the analysis of gene signatures, the aggregated expression of the respective genes was calculated using the AddModuleScore function from Seurat; cells were classified based on the aggregated expression using a modified version of the CellCycleScoring function that output only two classifications. The aggregated values for genes were calculated using a bin normalization approach for each individual gene and visualized on the UMAP embeddings through the FeaturePlot function.

DEGs were analyzed using Seurat’s FindMarkers function with default parameters and testing performed only on highly variable genes (two-sided Wilcoxon rank-sum test). DEG testing between cells incubated on ice with or without TP yielded three DEGs with nonsignificant *P* values; therefore, these samples were treated as one (‘ice’) for subsequent comparisons. As the dataset showed clear separation based on cell cycle phase classification, the samples were first compared cell cycle phase-wise. The DEGs between 37 °C with TP versus ice, 37 °C without TP versus ice across three cell cycle phases (G_1_/S/G_2_-M) were next used for MSigDB pathway analysis using Enrichr (https://maayanlab.cloud/Enrichr/).

All analysis steps except for the BCL to FASTQ conversion and the Cell Ranger run can be found in a Snakemake pipeline in the NCBI repository, with included conda (https://zenodo.org/record/4774217) environment specifications and version numbers of all packages used.

### Analysis of published microarray and bulk RNA-seq data

The analysis was performed using R v.4.1.0, v.4.1.3 and v.4.3.2. For the microarray data, raw.CEL files were retrieved from the Gene Expression Omnibus (GEO) and RMA-normalized using the affy v.1.76.0 or oligo v.1.62.2 packages. For bulk RNA-seq data analysis, count tables were downloaded from the GEO and data were normalized in R using the DESeq2 (ref. ^30^) v.1.38.3. When raw gene counts were not available, the normalized count tables provided by the authors were used. Genes with average expression counts less than the total number of samples were filtered out. GSEA^31^ was performed using normalized data of the input files and the following gene sets: MSigDB Hallmark provided by the software^5^ (MSigDB v.2022.1.Mm); custom-generated gene sets for IER and cell cycle; and aging and young signatures retrieved from Flohr Svendsen et al.^2^. All analyses were performed using the GSEA software v.4.3.2 (https://www.gsea-msigdb.org/gsea/index.jsp). The false discovery rate (FDR) method was applied to correct multiple hypotheses testing. Mining of gene lists was performed using the online tool Enrichr^32^ (https://maayanlab.cloud/Enrichr/) with default parameters. Graphs were generated using Prism v.9.5.1 (GraphPad Software).

For the analysis of stress-associated genes from van Velthoven et al.^19^ and Wu et al.^20^, data were downloaded from the GEO or using the supplementary tables provided by the authors. The DEGs with an FDR < 0.1 and fold change > 2 were used for comparison between IERsig genes identified in this study. The odds ratios (ORs) were computed in R by calculating the hypergeometric distribution, where the number of IERsig genes was 78 and the total number of genes was 19,570 (defined based on the total number of annotated genes on Affymetrix 430.2 microarrays from which the IERsig gene list was curated). The calculations of *P* values for the hypergeometric distributions were performed in R using a Fisher’s exact test.

For the analysis of the IERsig in the reference datasets on murine HSPCs, the R packages dplyr v.1.1.3 and ggplot2 v.3.4.4 were used. Briefly, the fraction of the IERsig in each sample was calculated by dividing the sum of gene expression levels for the IERsig genes by the total sum of gene expression levels in each sample. Data were visualized as dot plots with the fraction of IERsig across samples. To assess how the IERsig compared to randomly sampled gene sets, bootstrapping was used with a total of 10,000 iterations for each dataset. For reproducibility, a random seed (123) was set. In each iteration, a set of genes (RNA-seq data) or probe sets (microarrays), corresponding to the size of the IERsig, were randomly sampled from the gene expression data. The fraction of the bootstrapped genes in each sample was calculated in the same manner as for the IERsig. Descriptive statistics were computed for both the bootstrapped results and the IER signature fractions. To assess how the IER signature compared to the bootstrapped values, the centile rank of the coefficient of variation (CV) of the IER signature within the distribution of bootstrap CVs was computed and converted to a percentage. To visualize the results, histograms were generated to display the distribution of bootstrap CVs and the CV of the IER signature.

### Statistics and reproducibility

Data were analyzed using R v.4.1.0, v.4.1.3 and v.4.3.2, Microsoft Excel v.16.54 and Prism v.9.5.1 (GraphPad Software). All experiments were repeated as indicated; *n* indicates the number of independent biological replicates. No statistical methods were used to predetermine sample sizes but our sample sizes are similar to those reported in previous publications^3,4^. For the comparisons presented in Fig. 2d,h and Extended Data Fig. 5a, normality and equal variances were formally tested and data met the assumptions of the statistical tests used. Data collection and analysis were not performed blinded to the conditions of the experiments. For the transplantation and in vivo fixation experiments, no randomization method was applied to allocate animals to the experimental groups. Animals (*n* = 3–5) in the same cage received the same treatment. For the analyses of Nr4a1-GFP transgenic mice and the RT–qPCR experiments, no randomization method was applied and mice per sample were assigned to the experimental groups based on genotype or age. No animals or data were excluded from the analyses. For the flow cytometry analyses and RT–qPCR experiments, the analyses were performed in two or three independent experiments; no inconsistent results were observed. All results were successfully reproduced. The specific statistical test used for each experiment is indicated in the corresponding figure legend.

### Reporting summary

Further information on research design is available in the Nature Portfolio Reporting Summary linked to this article.

### Supplementary information

Reporting Summary

Supplementary Table 1 DEGs between batch 1 and combined batches 2 and 3 associated to ‘TNF signaling via NF-κB’ and ‘G_2_-M checkpoint’ pathway. Data derived from the GSEA. The FDR method was applied to correct for multiple hypotheses testing (Fig. 1b).

Supplementary Table 2 Detailed statistical information related to Fig. 1b,c,f and Extended Data Fig. 3b. Data were derived from the GSEA with FDR correction for multiple hypotheses testing. For the Enrichr analysis, a one-sided Fisher’s exact test with Benjamini–Hochberg correction was applied (computed using the Enrichr software).

Supplementary Table 3 Summary of the experimental procedures in the indicated transcriptome profiling studies on murine HSCs aging (Fig. 1f).

Supplementary Table 4 DEGs in HSPCs incubated on ice or at 37 °C ± TP (two-sided Wilcoxon rank-sum test) (Fig. 2i).

Supplementary Table 5 List of commercial reagents used in this study.

Supplementary Software The code for the bioinformatics analysis.

### Source data

Source Data Fig. 1 Statistical source data.

Source Data Fig. 2 Statistical source data.

Source Data Extended Data Fig. 1 Statistical source data.

Source Data Extended Data Fig. 3 Statistical source data.

Source Data Extended Data Fig. 4 Statistical source data.

Source Data Extended Data Fig. 5 Statistical source data.

## Data Availability

The original scRNA-seq data have been deposited in GEO under the accession no. GSE224590. The published datasets used for the analysis were retrieved from the GEO using the following accession numbers: HSC aging datasets (GSE27686, GSE44923, GSE55525, GSE6503, GSE48893, GSE39553, GSE47817, GSE127522, GSE128050, GSE151333, GSE156807, GSE109546, GSE157455 and GSE165982); datasets on muscle and neuronal cells (GSE97399, GSE103976, GSE15907); reference sets on murine HSPCs: GSE14833 and GSE6506 (bloodspot microarray); GSE34723 (gene expression commons microarray; GSE15907 (ImmGen microarray); GSE109125 (ImmGen ULI RNA-seq); and GSE132042 (Tabula Muris Senis RNA-seq data from BM cells). Other data generated in this study are available in the Source data files. Source data are provided with this paper.

## Code availability

The code for the bioinformatics analysis, alongside information on software and package versions, is available at https://github.com/razofz/DB_AKC_citeseq and in the Supplementary Software file.
