# Supplementary material for: Implications of stress-induced gene expression for hematopoietic stem cell aging studies
Source: Nat Aging. 2024 Jan 16;4(2):177–84. doi: 10.1038/s43587-023-00558-z (PMC10878961; doi:10.1038/s43587-023-00558-z)
Supplement: Supplementary file 1 — Reporting Summary [file 43587_2023_558_MOESM1_ESM.pdf]

nature portfolio

Corresponding author(s): Prof. David Bryder

Last updated by author(s): Dec 13, 2023

Reporting Summary

Nature Portfolio wishes to improve the reproducibility of the work that we publish. This form provides structure for consistency and transparency in reporting. For further information on Nature Portfolio policies, see our Editorial Policies and the Editorial Policy Checklist.

Statistics

For all statistical analyses, confirm that the following items are present in the figure legend, table legend, main text, or Methods section.

n/a	Confirmed	
☐	☒	The exact sample size (*n*) for each experimental group/condition, given as a discrete number and unit of measurement
☐	☒	A statement on whether measurements were taken from distinct samples or whether the same sample was measured repeatedly
☐	☒	The statistical test(s) used AND whether they are one- or two-sided<br>*Only common tests should be described solely by name; describe more complex techniques in the Methods section.*
☒	☐	A description of all covariates tested
☐	☒	A description of any assumptions or corrections, such as tests of normality and adjustment for multiple comparisons
☐	☒	A full description of the statistical parameters including central tendency (e.g. means) or other basic estimates (e.g. regression coefficient) AND variation (e.g. standard deviation) or associated estimates of uncertainty (e.g. confidence intervals)
☐	☒	For null hypothesis testing, the test statistic (e.g. *F*, *t*, *r*) with confidence intervals, effect sizes, degrees of freedom and *P* value noted<br>*Give P values as exact values whenever suitable.*
☒	☐	For Bayesian analysis, information on the choice of priors and Markov chain Monte Carlo settings
☒	☐	For hierarchical and complex designs, identification of the appropriate level for tests and full reporting of outcomes
☒	☐	Estimates of effect sizes (e.g. Cohen's *d*, Pearson's *r*), indicating how they were calculated

Our web collection on statistics for biologists contains articles on many of the points above.

Software and code

Policy information about availability of computer code

Data collection	Published datasets were retrived from Gene Expression Omnibus.<br>Flow cytometry data were collected using BD LSRFortessa X-20 and BD FACS Aria III instruments and FACS Diva software v9.0 (BD Bioscience).<br>Single cell RNA sequencing data were collected using Illumina NovaSeq6000 instrument.
Data analysis	For the analysis of published transcriptomic datasets, the following software were used: Cell Ranger v7.0.0, R v4.1.0, v4.1.3 and v4.3.2, Seurat, v4.1.1, affy v1.76.0, oligo v1.62.2, DESeq2 v1.38.3, MSigDB v2022.1.Mm, GSEA v4.3.2, dplyr v1.1.3, ggplot2 v3.4.4, Enrichr (https://maayanlab.cloud/Enrichr/), GraphPad Prism v9.5.1, Microsoft Excel v16.54. The FASTQ files were aligned to the mouse reference genome (mm10). All code for the single cell RNA sequencing analysis, with accompanying conda environments with package version specifications, can be found at https://github.com/razofz/DB_AKC_citeseq and in the Supplementary Software file.<br>Flow cytometry data were analyzed using FlowJo v10.5.3 (Treestar).

For manuscripts utilizing custom algorithms or software that are central to the research but not yet described in published literature, software must be made available to editors and reviewers. We strongly encourage code deposition in a community repository (e.g. GitHub). See the Nature Portfolio guidelines for submitting code & software for further information.

Data

Policy information about availability of data

All manuscripts must include a data availability statement. This statement should provide the following information, where applicable:

- Accession codes, unique identifiers, or web links for publicly available datasets
- A description of any restrictions on data availability
- For clinical datasets or third party data, please ensure that the statement adheres to our policy

Original single-cell RNA-sequencing data is deposited in GEO under the accession number GSE224590. Published datasets used for the analysis were retrieved from GEO using accession numbers: HSC aging datasets: GSE27686, GSE44923, GSE55525, GSE6503, GSE48893, GSE39553, GSE47817, GSE127522, GSE128050, GSE151333, GSE156807, GSE109546, GSE157455 and GSE165982; datasets on muscle and neuronal cells: GSE97399, GSE103976, GSE15907; reference sets on murine HSPCs: GSE14833 and GSE6506 (Bloodspot microarray), GSE34723 (Gene Expression Commons microarray), GSE15907 (ImmGen microarray) and GSE109125 (ImmGen ULI RNA-sequencing) and GSE132042 (Tabula Muris Senis RNA-sequencing data from BM cells). Other data generated in this study is available in the Source Data files.

Research involving human participants, their data, or biological material

Policy information about studies with human participants or human data. See also policy information about sex, gender (identity/presentation), and sexual orientation and race, ethnicity and racism.

Reporting on sex and gender	N/A
Reporting on race, ethnicity, or other socially relevant groupings	N/A
Population characteristics	N/A
Recruitment	N/A
Ethics oversight	N/A

Note that full information on the approval of the study protocol must also be provided in the manuscript.

Field-specific reporting

Please select the one below that is the best fit for your research. If you are not sure, read the appropriate sections before making your selection.

☒ Life sciences ☐ Behavioural & social sciences ☐ Ecological, evolutionary & environmental sciences

For a reference copy of the document with all sections, see nature.com/documents/nr-reporting-summary-flat.pdf

Life sciences study design

All studies must disclose on these points even when the disclosure is negative.

Sample size	No statistical methods were used to pre-determine sample sizes, but our sample sizes are similar to those reported in previous publications (Norddahl et al. 2011, Wahlestedt et al. 2013). For mice experiments, the number of mice is indicated in the corresponding figure legends. For single cell RNA sequencing, 40,000 LSK bone marrow cells were isolated from each sample and pooled prior to sequencing. In total 56,000 cells were loaded on 10x Chromium platform. For transplantation experiment, 250,000 donor and 1,000,000 competitor cKIT+ bone marrow cells were mixed and transplanted into each recipient mouse. For quantitative RT-PCR experiments, 5,000,000 cKIT+ bone marrow cells were isolated from young and aged mice and split into samples for time course analyses (700,000 cells per each sample). For ex vivo HSPC fixation, 4,000,000 cKIT+ bone marrow cells were isolated from young mice and split into four samples (~900,000 cells per each sample) for subsequent RNA integrity analyses.
Data exclusions	No data were excluded.
Replication	For flow cytometry analysis of Nr4a1-GFP signal in bone marrow cells, a total of n= 3-4 mice/genotype were analyzed in three independent experiments. Quantitative RT-PCR analyses were performed in two independent experiments. For HSPC analysis following in vivo fixation, a total of n=7-8 mice/group were analyzed in three independent experiments. For all experiments no inconsistent results were observed. The results were successfully replicated. Transplantation analysis was performed in one replicate with n=5 recipients/group.
Randomization	Adult (8-30 weeks old) and aged (20 months) female and male mice were used. For transplantation experiment, female mice were used due to housing and maintenance convenience. For transplantation and in vivo fixation experiments, a simple randomization procedure was applied to cages of mice, such that n = 3-5 animals per cage were assigned to each group and all animals within a cage received the same treatment. For analyses of Nr4a1-GFP transgenic mice and quantitative real-time PCR experiments, no randomization method was applied and the mice/samples were assigned to experimental groups based on genotype or age.

Blinding

In all experiments, samples and groups were known, as information on the treatment (cell isolation procedure) was required for data collection and analysis.

Reporting for specific materials, systems and methods

We require information from authors about some types of materials, experimental systems and methods used in many studies. Here, indicate whether each material, system or method listed is relevant to your study. If you are not sure if a list item applies to your research, read the appropriate section before selecting a response.

Materials & experimental systems

n/a	Involved in the study
☐	☒ Antibodies
☒	☐ Eukaryotic cell lines
☒	☐ Palaeontology and archaeology
☐	☒ Animals and other organisms
☒	☐ Clinical data
☒	☐ Dual use research of concern
☒	☐ Plants

Methods

n/a	Involved in the study
☒	☐ ChIP-seq
☐	☒ Flow cytometry
☒	☐ MRI-based neuroimaging

Antibodies

Antibodies used

CD11b – APC (Sony Biotechnology, Cat# 1106060, clone M1/70), dilution 1:800
CD117 - APC (Sony Biotechnology, Cat# 1129060, clone 2B8), dilution 1:200
CD117 - APCeFluor780 (eBioscience, Cat# 47-1171-82, clone 2B8), dilution 1:200
CD135 - PE (Sony Biotechnology, Cat# 1276530, clone A2F10), dilution 1:100
CD150 - PE/Cy7 (Sony Biotechnology, Cat# 1179570, clone TC15-12F12.2), dilution 1:200
CD19 - PE/Cy7 (Sony Biotechnology, Cat# 1177600, clone 6D5), dilution 1:200
CD201 - APC (eBioscience, Cat# 17-2012-82, clone eBio1560), dilution 1:200
CD3 - Alexa Fluor 700 (Sony Biotechnology, Cat# 1101080, clone 17A1), dilution 1:200
CD3 - Biotin (Sony Biotechnology, Cat# 1101220, clone 17A1), dilution 1:200
CD3 - PE/Cy5 (Sony Biotechnology, Cat# 1101550, clone 145-2C11), dilution 1:200
CD45.1 - Brilliant Violet 650 (Sony Biotechnology, Cat# 1153680, clone A20), dilution 1:100
CD45.2 - Brilliant Violet 785 (Sony Biotechnology, Cat# 1149195, clone 104), dilution 1:100
CD45R/B220 - Biotin (Sony Biotechnology, Cat# 1116020, clone RA3-6B2), dilution 1:200
CD45R/B220 - PE/Cy5 (Sony Biotechnology, Cat# 1116050, clone RA3-6B2), dilution 1:200
CD48 - FITC (Sony Biotechnology, Cat# 1117020, clone HM48-1), dilution 1:200
NK1.1 - Biotin (BD Bioscience, Cat# 553163, clone PK136), dilution 1:200
NK1.1 - PE/Cy5 (Sony Biotechnology, Cat# 1143580, clone PK136), dilution 1:200
NK1.1 - Pacific Blue (Sony Biotechnology, Cat# 1143610, clone PK136), dilution 1:200
Ly6A/E (Sca-1) - Pacific Blue (Biolegend, Cat# 122520, clone E13-161.7), dilution 1:200
Ly6G/Ly6C (Gr-1) - Biotin (Sony Biotechnology, Cat# 1142020, clone RB6-8C5), dilution 1:400
Ly6G/Ly6C (Gr-1) - PE/Cy5 (Sony Biotechnology, Cat# 1142050, clone RB6-8C5), dilution 1:400
Streptavidin - Brilliant Violet 605 (Sony Biotechnology, Cat# 2626145), dilution 1:400
Ter119 - Biotin (Sony Biotechnology, Cat# 1181020, clone TER119), dilution 1:400
Ter119 - PE/Cy5 (Sony Biotechnology, Cat# 1181050, clone TER119), dilution 1:400
Ter119 - PerCP/Cy5.5 (Sony Biotechnology, Cat# 1181140, clone TER119), dilution 1:400
Hashtag 1 - TotalSeq-A0301 (Biolegend, Cat# 155801, clone M1/42; 30-F11), 0.5 ug/sample (up to 10 mln cells)
Hashtag 2 - TotalSeq-A0302 (Biolegend, Cat# 155803, clone M1/42; 30-F11), 0.5 ug/sample (up to 10 mln cells)
Hashtag 3 - TotalSeq-A0303 (Biolegend, Cat# 155805, clone M1/42; 30-F11), 0.5 ug/sample (up to 10 mln cells)
Hashtag 4 - TotalSeq-A0304 (Biolegend, Cat# 155807, clone M1/42; 30-F11), 0.5 ug/sample (up to 10 mln cells)

Validation

The antibodies were validated for the specified applications by the manufacturer. Validations profiles and references can be found in the provided links. All antibodies have been used in test panels prior to experimental procedures to determine optimal dilutions.
CD11b – APC; species reactivity: Mouse, Human; tested application: FC, product citations: 32; https://www.sonybiotechnology.com/us/catalog/product/view/id/360/s/apc-anti-mouse-human-cd11b/
CD117 – APC; species reactivity: Mouse; tested application: FC, product citations: 7; https://www.sonybiotechnology.com/us/apc-anti-mouse-cd117-c-kit-13
CD117 - APCeFluor780; species reactivity: Mouse, Human; tested application: FC, FN, IV; product citations: 88; https://www.thermofisher.com/antibody/product/CD117-c-Kit-Antibody-clone-2B8-Monoclonal/47-1171-82
CD135 – PE; species reactivity: Mouse; tested application: FC, product citations: 3; https://www.sonybiotechnology.com/se/pe-anti-mouse-cd135-7
CD150 - PE/Cy7; species reactivity: Mouse; tested application: FC, product citations: 22; https://www.sonybiotechnology.com/se/pe-cy7-anti-mouse-cd150-slam-7
CD19 - PE/Cy7; species reactivity: Mouse; tested application: FC, product citations: 12; https://www.sonybiotechnology.com/se/pe-cy7-anti-mouse-cd19-7
CD201 – APC; species reactivity: Mouse; tested application: FC, product citations: 12; https://www.thermofisher.com/antibody/product/CD201-EPCR-Antibody-clone-eBio1560-1560-Monoclonal/17-2012-82
CD3 - Alexa Fluor 700; species reactivity: Mouse; tested application: FC, product citations: 9; https://www.sonybiotechnology.com/

us/alexa-fluor-reg-700-anti-mouse-cd3-6
CD3 – Biotin; species reactivity: Mouse; tested application: FC, product citations: 8; https://www.sonybiotechnology.com/se/biotin-anti-mouse-cd3-7
CD3 - PE/Cy5; species reactivity: Mouse; tested application: FC, product citations: 31; https://www.sonybiotechnology.com/se/pe-cy5-anti-mouse-cd3-epsilon-5
CD45.1 - Brilliant Violet 650; species reactivity: Mouse; tested application: FC, product citations: 21; https://www.sonybiotechnology.com/se/brilliant-violet-650-trade-anti-mouse-cd45-1-7
CD45.2 - Brilliant Violet 785; species reactivity: Mouse; tested application: FC, product citations: 12; https://www.sonybiotechnology.com/us/brilliant-violet-785-trade-anti-mouse-cd45-2-3
CD45R/B220 – Biotin; species reactivity: Mouse, Human; tested application: FC, product citations: 13; https://www.sonybiotechnology.com/se/biotin-anti-mouse-human-cd45r-b220-7
CD45R/B220 - PE/Cy5; species reactivity: Mouse, Human; tested application: FC, product citations: 13; https://www.sonybiotechnology.com/se/pe-cy5-anti-mouse-human-cd45r-b220-7
CD48 – FITC; species reactivity: Mouse; tested application: FC, product citations: 6; https://www.sonybiotechnology.com/se/fitc-anti-mouse-cd48-7
NK1.1 – Biotin; species reactivity: Mouse; tested application: FC, product citations: 11; https://www.bdbiosciences.com/en-us/products/reagents/flow-cytometry-reagents/research-reagents/single-color-antibodies-ruo/biotin-mouse-anti-mouse-nk-1-1.553163
NK1.1 - PE/Cy5; species reactivity: Mouse; tested application: FC, product citations: 15; https://www.sonybiotechnology.com/se/pe-cy5-anti-mouse-nk-1-1-7
NK1.1 - Pacific Blue; species reactivity: Mouse; tested application: IF, product citations: 15; https://www.sonybiotechnology.com/us/pacific-blue-trade-anti-mouse-nk-1-1-6
Ly6A/E (SCA-1) - Pacific Blue; species reactivity: Mouse; tested application: FC, product citations: 31; https://www.biolegend.com/en-us/products/pacific-blue-anti-mouse-ly-6a-e-sca-1-antibody-3901
Ly6G/Ly6C (Gr-1) – Biotin; species reactivity: Mouse; tested application: FC, product citations: 21; https://www.sonybiotechnology.com/se/biotin-anti-mouse-ly-6g-ly-6c-gr-1-7
Ly6G/Ly6C (Gr-1) - PE/Cy5; species reactivity: Mouse; tested application: FC, product citations: 22; https://www.sonybiotechnology.com/se/pe-cy5-anti-mouse-ly-6g-ly-6c-gr-1-7
TER119 – Biotin; species reactivity: Mouse; tested application: FC, product citations: 8; https://www.sonybiotechnology.com/se/biotin-anti-mouse-ter-119-erythroid-cells-7
TER119 - PE/Cy5; species reactivity: Mouse; tested application: FC, product citations: 7; https://www.sonybiotechnology.com/se/pe-cy5-anti-mouse-ter-119-erythroid-cells-7
TER119 - PerCP/Cy5.5; species reactivity: Mouse; tested application: FC, product citations: 8; https://www.sonybiotechnology.com/se/percp-cy5-5-anti-mouse-ter-119-erythroid-cells-6
Hashtag 1 - TotalSeq-A0301; species reactivity: Mouse; tested application: PG, product citations: 13; https://www.biolegend.com/en-us/products/totalseq-a0301-anti-mouse-hashtag-1-antibody-16103
Hashtag 2 - TotalSeq-A0302; species reactivity: Mouse; tested application: PG, product citations: 11; https://www.biolegend.com/en-us/products/totalseq-a0302-anti-mouse-hashtag-2-antibody-16104
Hashtag 3 - TotalSeq-A0303; species reactivity: Mouse; tested application: PG, product citations: 9; https://www.biolegend.com/en-us/products/totalseq-a0303-anti-mouse-hashtag-3-antibody-16105
Hashtag 4 - TotalSeq-A0304, species reactivity: Mouse; tested application: PG, product citations: 8; https://www.biolegend.com/en-us/products/totalseq-a0304-anti-mouse-hashtag-4-antibody-16106

Animals and other research organisms

Policy information about studies involving animals; ARRIVE guidelines recommended for reporting animal research, and Sex and Gender in Research

Laboratory animals	Mus musculus, Nr4a1-GFP (JAX, #016617), 22-32 weeks old, female mice<br>Mus musculus, C57BL/6NTac (Taconic Biosciences, #B6), 8-12 weeks old and 20 months old, males and female mice<br>Mus musculus, C57BL/6.SJL - CD45.1 (in-house breeding), 8-12 weeks old, female mice<br>All mice were housed in a controlled environment with 12-hour light-dark cycles with chow and water provided ad libitum.
Wild animals	This study did not involve wild animals.
Reporting on sex	For transplantation experiments, analyses of Nr4a1-GFP reporter cells and single cell RNA sequencing, the cells were isolated from female mice. For quantitative RT-PCR experiments, the cells were isolated from male mice. For in vivo and ex vivo fixation, the cells were isolated from female mice.
Field-collected samples	This study did not involve samples collected from the field.
Ethics oversight	All animal experiments performed in this study were approved by the Lund University Ethical Committee (#16468-20)

Note that full information on the approval of the study protocol must also be provided in the manuscript.

Flow Cytometry

Plots

Confirm that:

- [x] The axis labels state the marker and fluorochrome used (e.g. CD4-FITC).
- [x] The axis scales are clearly visible. Include numbers along axes only for bottom left plot of group (a 'group' is an analysis of identical markers).
- [x] All plots are contour plots with outliers or pseudocolor plots.
- [x] A numerical value for number of cells or percentage (with statistics) is provided.

Methodology

Sample preparation	Bone marrow cells were isolated from tibia, femur, and pelvis into ice-cold FACS buffer (2% FBS/PBS) with or without 5 uM Triptolide (Tocris). The cells were cKIT-enriched by anti-cKIT-APC staining, followed by incubation with anti-APC MicroBeads and magnetic separation on LS columns (Miltenyi Biotec). Aliquots of cells were resuspended in culture media (DMEM with high glucose/10 mM HEPES/2% FBS) with or without Triptolide (0.1, 0.5, 1 and 5 uM) and incubated on ice or at 37ºC for 90 min. Following incubation, the cells were stained with biotinylated antibodies against B220, Gr-1, TER119, CD3, NK1.1, SCA-1-Pacific Blue (Sony Biotechnology) for 30 min on ice in the dark. For single cell RNA sequencing, the cells were additionally stained with oligo-conjugated hashing antibodies (TotalSeq-A0301, -A0302, -A0303, and -A0304, BioLegend). The secondary staining was performed with streptavidin-BV605 (Sony Biotechnology). For peripheral blood analysis by flow cytometry, blood samples were sedimented with 1% Dextran T500 (Sigma-Aldrich) for 30 min at 37°C, and remaining erythrocytes were lysed using ammonium chloride solution (STEMCELL Technologies) for 3 min at room temperature. The cells were stained with CD19-PE-Cy7 (dilution 1:200), TER119-PerCP-Cy5.5 (dilution 1:400), CD11B-APC (dilution 1:800), NK1.1-Pacific Blue (dilution 1:200), CD3-Alexa Fluor700 (dilution 1:200), CD45.1-BV650 (dilution 1:100) and CD45.2-BV785 (dilution 1:100) (Sony Biotechnology). For analysis of in vivo-fixed HSPCs, unfractionated BM cells were stained with PE-Cy5-conjugated B220, Gr-1, TER119, CD3, NK1.1, SCA-1-Pacific Blue, CD48-FITC, CD150-PE-Cy7, CD135-PE (Sony Biotechnology) and cKIT-APC-eFluor780, CD201-APC (eBioscience) antibodies. Prior to analysis or sorting, cells were stained with PI (1:1000, Invitrogen) to exclude dead cells.
Instrument	LSRFortessa X-20 analyzer (BD Bioscience)<br>BD FACSAria III cell sorter (BD Bioscience)
Software	BD Diva software v9.0 (BD Bioscience)<br>FlowJo 10 software v10.5.3 (Treestar)
Cell population abundance	For single cell RNA sequencing, 40,000 Lin-SCA-1+cKIT+ bone marrow cells were sorted from each sample. Sort purity was determined by re-analysis of sorted cells.
Gating strategy	For cell sorting, single cells were gated using FSC-A/FSC-H and live nucleated cells were gated based on SSC-A/FSC-A and PI staining. Lineage-, SCA-1+ and cKIT+ gating was set based on internal reference populations.<br>For peripheral blood analysis, single cells were gated using FSC-A/FSC-H and live nucleated cells were gated based on SSC-A/FSC-A, PI and TER119 staining. Cells were subsequently gated based on CD19, CD3, CD11B and NK1.1 expression. Donor- and competitor-derived cells were discriminated by gating CD45.1+ (donor) and CD45.2+ (competitor) cells within mature peripheral blood cell subsets. Gating strategy for all flow cytometry and FACS sorting experiments are included in Extended Data Figures 2, 3 and 5.

- [x] Tick this box to confirm that a figure exemplifying the gating strategy is provided in the Supplementary Information.
