# Supplementary material for: Implications of stress-induced gene expression for hematopoietic stem cell aging studies
Source: Nat Aging. 2024 Jan 16;4(2):177–84. doi: 10.1038/s43587-023-00558-z (PMC10878961; doi:10.1038/s43587-023-00558-z)

**HSPC stress response to cell isolation procedure measured by scRNAseq**

**AIM:** Aim of this experiment is to determine cellular stress in response to different HSPC isolation procedures. In addition, samples will be stained with CITE-seq antibodies to evaluate transcriptomic signatures of MPP Ly populations.

Groups:

1) BM cells isolated in FACS buffer

2) BM cells isolated in FACS buffer and incubated at 37ºC for 90 min

3) BM cells isolated in FACS buffer + 5 uM triptolide

4) BM cells isolated in FACS buffer + 5 uM triptolide and incubated at 37ºC for 90 min

**MICE AND REAGENTS**

Mice: 5 females, 8 weeks old B6SJL

Buffers:

FACS buffer: ice-cold PBS + 2% FBS

FACS buffer/Triptolide: ice-cold PBS + 2% FBS + 5 uM triptolide

FACS antibodies:

purified CD16/32 (Fc block)

B220 - biotin

Gr1 - biotin

Ter119 - biotin

CD3 - biotin

NK1 - biotin

SCA-1 - Pacific Blue

cKIT - APC

Streptavidin – BV605

CITE-seq antibodies [0.5mg/ml]:

CD48 – TotalSeq-A0429

CD150 – TotalSeq-A0203

CD135 – TotalSeq-A0098

CD201 – TotalSeq-A0439

Hashtag#1 – TotalSeq-A0301

Hashtag#2 – TotalSeq-A0302

Hashtag#3 – TotalSeq-A0303

Hashtag#4 – TotalSeq-A0304

Cell culture media:

Culture media: DMEM + 10 mM HEPES + 2% FBS

Culture media + triptolide: DMEM + 10 mM HEPES + 2% FBS + 5 uM triptolide

**EXPERIMENT OVERVIEW**

**
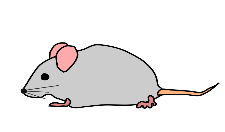
**

**
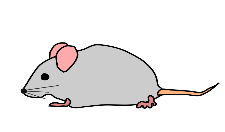
**

BM isolation

**Standard buffer**

cKit enrichment

½ sample

½ sample

90 min incubation at 37ºC

BM isolation

**Buffer with triptolide**

cKit enrichment

½ sample

½ sample

90 min incubation at 37ºC

90 min incubation on ice

90 min incubation on ice

Staining with FACS- and CITE-seq antibodies AND:

Hashtag#2

**(Sample 2)**

Hashtag#1

**(Sample 1)**

Hashtag#4

**(Sample 4)**

Hashtag#3

**(Sample 3)**

LSK sort (40 K/sample) into one collection tube.

Sorting in the following order:

1) Sample 4 (37ºC + triptolide)

2) Sample 3 (triptolide)

3) Sample 2 (37ºC)

4) Sample 1 (no treatment)

**PROCEDURE**

Keep the cells on ice at all steps with the exception of 37ºC incubation.

1. Harvest and pool BM cells from 2.5 mice into 5 ml ice-cold FACS buffer or FACS buffer/Triptolide.
2. Crush bones using mortar and pestle using 10 ml of respective FACS buffer (make sure to use separate mortars).
3. Filter the cells and spin down for 10 min, 400g, 4ºC.
4. Resuspend the cells in 2.5 ml cKIT-APC staining media (1ul antibody/1ml buffer/mouse):
5. 3 ml FACS buffer + 3 ul cKIT-APC
6. 3 ml FACS buffer/Triptolide + 3 ul cKIT-APC
7. Incubate for 30 min on ice.
8. Wash the cells by adding 8 ml of respective FACS buffer.
9. Spin down for 10 min, 400g, 4ºC.
10. Resuspend the cells in 1250 ul anti-APC beads staining media (25ul beads/500ul buffer/mouse):
11. 1235 ul FACS buffer + 65 ul anti-APC beads
12. 1235 ul FACS buffer/Triptolide + 65 ul anti-APC beads
13. Incubate for 30 min on ice.
14. Wash the cells by adding 8 ml of respective FACS buffer.
15. Spin down for 10 min, 400g, 4ºC.
16. Resuspend the cells in 1 ml of respective FACS buffer.
17. Perform positive selection using two Miltenyi LS column.
    1. Prepare each LS column by rinsing with 2 ml of respective FACS buffer.
    2. Apply the cells onto each column.
    3. Wash columns with 3 x 3 ml of respective FACS buffer.
    4. Remove columns from magnet and flush out positive fractions with 5 ml of respective FACS buffer into 15 ml tubes. Repeat this step twice (final elution volume is 10 ml).
18. Count the cells.
19. Spin down the cells for 10 min, 400g, 4ºC.
20. Resuspend samples 2 ml of DMEM/HEPES/FBS or DMEM/HEPES/FBS/Triptolide.
21. Transfer 1 ml from each sample into new 15 ml falcon tube. Label the samples #1-4 as shown in scheme on page 2.
22. Incubate samples #1 and #3 on ice and #2 and #4 at 37ºC for 90 min.
23. Spin down for 10 min, 400g, 4ºC.
24. Resuspend the cells in 50 ul of respective FACS buffers supplemented with Fc block (1:50). Incubate for 10 min on ice.
25. Prepare two FACS- and CITE-seq antibody mixes in 110 ul of respective FACS buffers (antibodies are 2x concentrated; their final dilutions are included in brackets):

Biotinylated B220 (1:200), Gr1 (1:400), Ter119 (1:400), CD3 (1:200), NK1.1 (1:200)

SCA-1 - Pacific Blue (1:200)

CD48, CD150, CD201, CD135 – TotalSeq-A (1:100 each [2.2ul of each Abs])

1. Add 50 ul of antibody mixes to respective samples with Fc block in FACS buffer +/- Triptolide.
2. Add 1 ul of one of Hashtags-Ab #1-4 to each tube. Resuspend well.

The samples are stained in 100 ul staining mixes with 0.5 ug of each CITE- and hashing antibodies/sample (~6 mln cKit-enriched cells/sample).

1. Incubate for 30 min on ice.
2. Wash the cells by adding 8 ml of respective FACS buffer.
3. Spin down the cells for 10 min, 400g, 4ºC.
4. Repeat steps 27-28 twice.
5. Resuspend the cells in 100 ul of respective FACS buffers supplemented with streptavidin-BV605 (1:400.)
6. Incubate for 30 min on ice.
7. Wash the cells by adding 8 ml of respective FACS buffer.
8. Resuspend the cells in 500 ul of respective FACS buffer supplemented with PI (1:1000).
9. Filter the cells.
10. Keep the cells on ice until sorting.
11. Sort 40,000 Lineage-Sca1+cKit+ cells from each sample into one 1.5 ml low retention tube with 500 ul FACS buffer (without Triptolide).

Sort the cells in the following order:

1) Sample 4 (37ºC + triptolide)

2) Sample 3 (triptolide)

3) Sample 2 (37ºC)

4) Sample 1 (no treatment)

**FACS-parameters**
Lineage BV605

c-kit APC

Sca1 Pacific Blue
Viability PI

1. After sorting, spin down the cells for 5 min, 400g, 4ºC.
2. Resuspend cell pellet in 50 ul FACS buffer.
3. Count the cells.
4. Dilute the sample by adding FACS buffer to get the final concentration of 1,200 cells/ul (51K cells should be in 42.5 ul which is the volume loaded onto Chromium).

Final number of cells after sorting and spinning down: 112,560 LSK

The sample was diluted to final concentration: 1,250 cells/ul

Cell number loaded onto Chromium platform: 54,000 cells

220331 TEST RUN

Mouse: 13 weeks old male (MLL-ENL CD45.1 strain, noninduced)

WBC cellularity: 120 mln

cKIT-enriched sample: 4.4 mln

Total number of sorted LSKs: 110K cells in ~10 min

Sample spin down, resuspension in 100 ul and cell counting: 78.1K cells


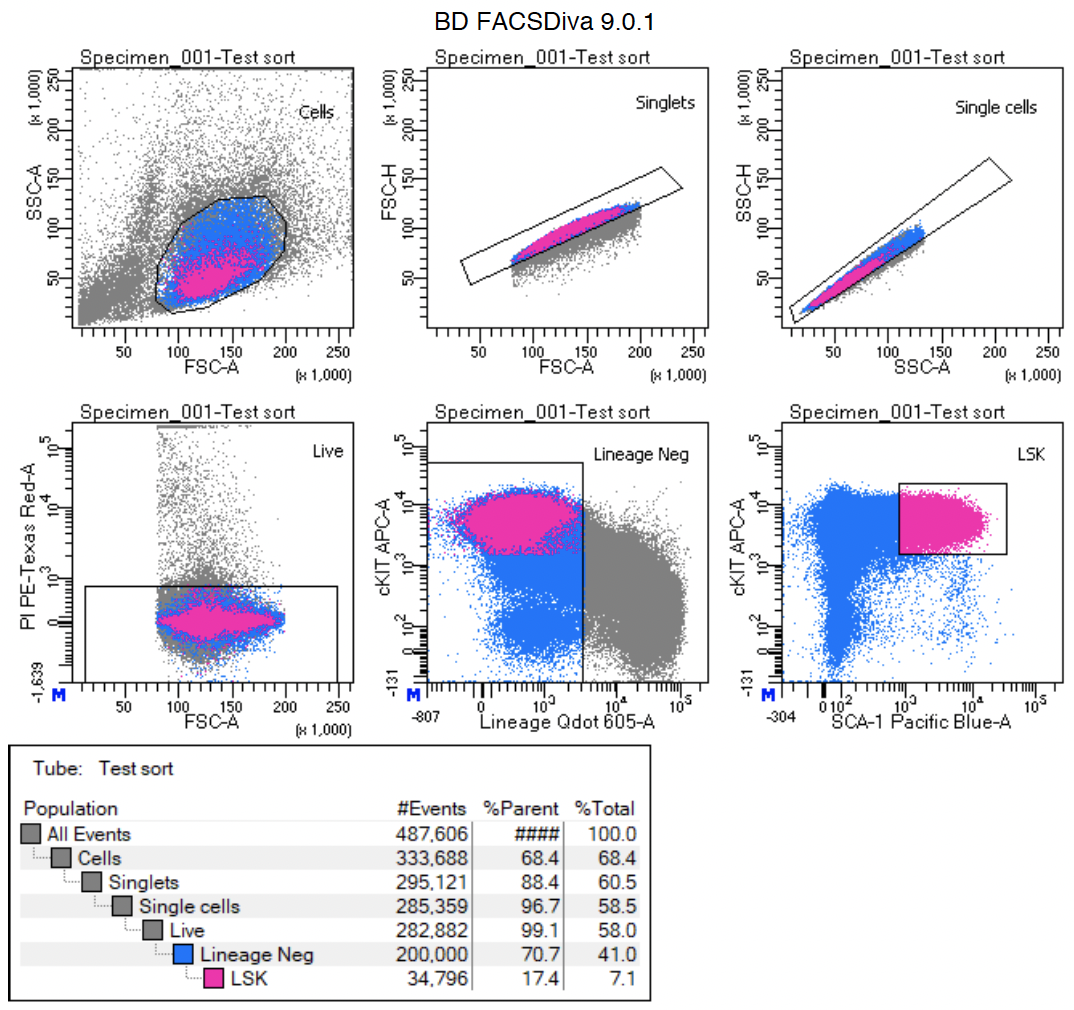


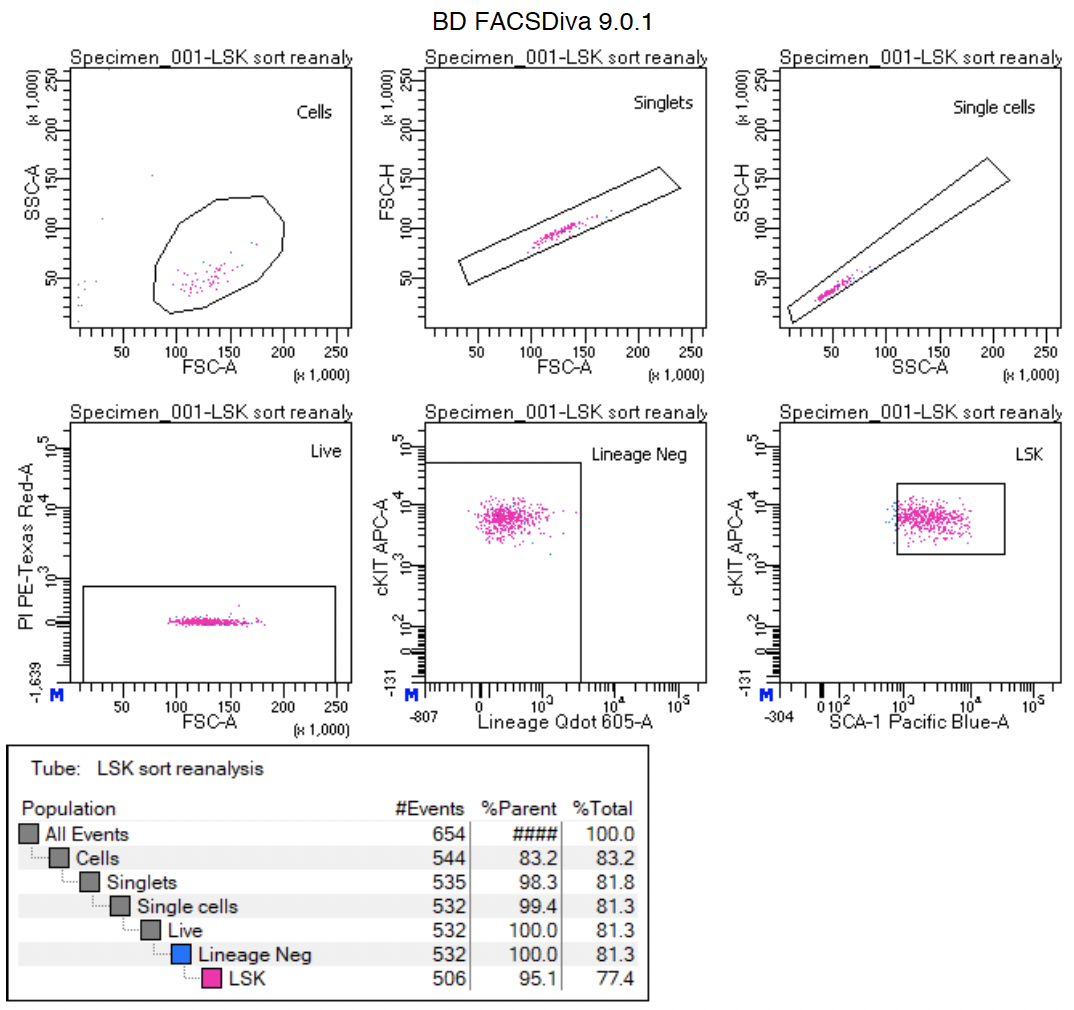

Supplement: Supplementary file 7 — The code for the bioinformatics analysis. [file 43587_2023_558_MOESM7_ESM.tar › references/220331_10x_protocol.docx]
