# Supplementary material for: Implications of stress-induced gene expression for hematopoietic stem cell aging studies
Source: Nat Aging. 2024 Jan 16;4(2):177–84. doi: 10.1038/s43587-023-00558-z (PMC10878961; doi:10.1038/s43587-023-00558-z)
Supplement: Supplementary file 7 — The code for the bioinformatics analysis. [file 43587_2023_558_MOESM7_ESM.tar › notebooks/sc_qc.r.html]

Revision plots, IER manuscript


## Table of contents

- Methods
  - Differentially expressed genes (DEG)
  - Gene signatures
- Setup
- Visualization of dataset with conditions, for reference
- Compare ice conditions
- Compare 37c conditions
  - Save DEG tables
  - Overlapping DEGs (intersection between cell cycle phases)
- Collated dot plots
  - Dot plots overlapping DEGs
- QC plots
- Signatures

# Revision plots, IER manuscript

Author

Rasmus Olofzon


---

## Methods

### Differentially expressed genes (DEG)

The samples were first compared cell cycle phase-wise, since the dataset showed clear separation based on phase classification, but its structure otherwise made biological sense. In the UMAP the two ice conditions co-located very clearly, which prompted the notion of treating them as one for the DEG analysis. This was corroborated by a DEG testing between the two ice conditions, which for the samples as wholes yielded three DEGs, non with significant p-values. Phase-wise no DEGs were found.

Based off of that, three comparisons were carried out:

1. 37c *with* triptolide VS ice
2. 37c *without* triptolide VS ice
3. 37c *without* triptolide VS 37c *without* triptolide

The comparisons were first made cell cycle phase-wise (G1, G2M, S), then the intersection of the found DEGs was taken. This would give the differentially expressed genes that most characterizes a given condition, without cell cycle-specific genes.

The DEG testing was performed with Seurat’s `FindMarkers` function, testing performed only on highly variable genes (HVGs). The intersection was taken with R’s `intersect` function for sets.

### Gene signatures

The gene signature scores were calculated and visualized similar to the procedure for the IER signature.

---

# Setup

```
library(Seurat)
library(stringr)
library(dplyr)
library(ggplot2)
```

```
Attaching SeuratObject


Attaching package: ‘dplyr’


The following objects are masked from ‘package:stats’:

    filter, lag


The following objects are masked from ‘package:base’:

    intersect, setdiff, setequal, union
```

```
library(tidyr)
library(dplyr)
library(viridis)
```

```
Loading required package: viridisLite
```

```
library(patchwork)
```

```
sobj <- readRDS("../data/processed/seurat_object_w_stress_sig.rds")
```

```
# cols_features <- c("moccasin", "darkslategray")
cols_features <- c("lightgray", "red3")
```

```
phases <- list("G1", "G2M", "S")
names(phases) <- phases
phases
```

$G1
:   'G1'

$G2M
:   'G2M'

$S
:   'S'

---

# Visualization of dataset with conditions, for reference

Metadata:

```
sobj[[]] %>% head
```

Table 1: Metadata for dataset

|  | orig.ident | nCount\_RNA | nFeature\_RNA | nCount\_ADT | nFeature\_ADT | nCount\_HTO | nFeature\_HTO | percent.mt | hto | sample | ⋯ | buffer\_treatment | incubation\_method | S.Score | G2M.Score | Phase | old.ident | RNA\_snn\_res.0.8 | seurat\_clusters | stress\_signature1 | is\_stressed |
| --- | --- | --- | --- | --- | --- | --- | --- | --- | --- | --- | --- | --- | --- | --- | --- | --- | --- | --- | --- | --- | --- |
|  | <chr> | <dbl> | <int> | <dbl> | <int> | <dbl> | <int> | <dbl> | <chr> | <chr> | ⋯ | <chr> | <chr> | <dbl> | <dbl> | <chr> | <fct> | <fct> | <fct> | <dbl> | <chr> |
| AAACCCAAGAGACAAG-1 | DB\_AKC\_citeseq | 11976 | 3437 | 128 | 4 | 32 | 3 | 2.104208 | HTO2 | 37c\_no\_t | ⋯ | no\_triptolide | 37c | -0.2740383 | -0.19990412 | G1 | HTO2 | 0 | 0 | 0.19599848 | stressed |
| AAACCCAAGAGTGAAG-1 | DB\_AKC\_citeseq | 21028 | 5475 | 54 | 4 | 108 | 4 | 2.425338 | HTO3 | ice\_t | ⋯ | with\_triptolide | ice | 0.2386460 | -0.01205648 | S | HTO3 | 1 | 1 | -0.10710847 | not\_stressed |
| AAACCCAAGCGAAACC-1 | DB\_AKC\_citeseq | 10688 | 2813 | 56 | 3 | 87 | 2 | 4.519087 | HTO4 | 37c\_t | ⋯ | with\_triptolide | 37c | -0.1966232 | -0.14132145 | G1 | HTO4 | 5 | 5 | -0.04234327 | not\_stressed |
| AAACCCAAGGTAAAGG-1 | DB\_AKC\_citeseq | 10627 | 3677 | 68 | 4 | 79 | 4 | 2.888868 | HTO1 | ice\_no\_t | ⋯ | no\_triptolide | ice | -0.1555948 | -0.21232288 | G1 | HTO1 | 2 | 2 | -0.04919840 | not\_stressed |
| AAACCCAAGGTCTACT-1 | DB\_AKC\_citeseq | 16865 | 3915 | 106 | 4 | 99 | 2 | 2.514082 | HTO4 | 37c\_t | ⋯ | with\_triptolide | 37c | 0.2038750 | 0.17149954 | S | HTO4 | 7 | 7 | -0.08366769 | not\_stressed |
| AAACCCAAGTCGGCCT-1 | DB\_AKC\_citeseq | 13939 | 4192 | 60 | 3 | 75 | 2 | 3.285745 | HTO3 | ice\_t | ⋯ | with\_triptolide | ice | -0.3008664 | -0.25975436 | G1 | HTO3 | 2 | 2 | -0.09381362 | not\_stressed |

```
DimPlot(sobj, group.by = "sample") + coord_fixed()
DimPlot(sobj, group.by = "Phase") + coord_fixed()
```

Figure 1: UMAP coloured on metadata

(a) Coloured on condition/sample

(b) Coloured on cell cycle phases

```
df <- table(sobj[[]][,c("sample", "Phase")]) %>% as.data.frame.matrix #%>% rbind(
# table(sobj[[]][,c("sample")]) %>% as.list
# )
df
```

A data.frame: 4 × 3

|  | G1 | G2M | S |
| --- | --- | --- | --- |
|  | <int> | <int> | <int> |
| 37c\_no\_t | 2164 | 334 | 375 |
| 37c\_t | 1799 | 608 | 1173 |
| ice\_no\_t | 1825 | 351 | 872 |
| ice\_t | 1760 | 360 | 945 |

```
df[["sample"]] <- table(sobj[["sample"]]) %>% as.list %>% as.numeric
df
```

A data.frame: 4 × 4

|  | G1 | G2M | S | sample |
| --- | --- | --- | --- | --- |
|  | <int> | <int> | <int> | <dbl> |
| 37c\_no\_t | 2164 | 334 | 375 | 2873 |
| 37c\_t | 1799 | 608 | 1173 | 3580 |
| ice\_no\_t | 1825 | 351 | 872 | 3048 |
| ice\_t | 1760 | 360 | 945 | 3065 |

```
df <- df %>% mutate(
    G1_frac = round(G1 / sample, digits = 2),
    G2M_frac = round(G2M / sample, digits = 2),
    S_frac = round(S / sample, digits = 2),
)
df
```

A data.frame: 4 × 7

|  | G1 | G2M | S | sample | G1\_frac | G2M\_frac | S\_frac |
| --- | --- | --- | --- | --- | --- | --- | --- |
|  | <int> | <int> | <int> | <dbl> | <dbl> | <dbl> | <dbl> |
| 37c\_no\_t | 2164 | 334 | 375 | 2873 | 0.75 | 0.12 | 0.13 |
| 37c\_t | 1799 | 608 | 1173 | 3580 | 0.50 | 0.17 | 0.33 |
| ice\_no\_t | 1825 | 351 | 872 | 3048 | 0.60 | 0.12 | 0.29 |
| ice\_t | 1760 | 360 | 945 | 3065 | 0.57 | 0.12 | 0.31 |

```
df <- table(sobj[[]][,c("sample", "Phase")]) %>% as.data.frame.matrix #%>% rbind(
# table(sobj[[]][,c("sample")]) %>% as.list
# )
df
df <- df %>% tibble::rownames_to_column(var = "sample") %>% pivot_longer(cols = c("G1", "G2M", "S"), names_to = "phase", values_to="n_cells")
df
```

Table 2: Cell cycle phase classification distribution per condition

(a) A data.frame: 4 × 3

|  | G1 | G2M | S |
| --- | --- | --- | --- |
|  | <int> | <int> | <int> |
| 37c\_no\_t | 2164 | 334 | 375 |
| 37c\_t | 1799 | 608 | 1173 |
| ice\_no\_t | 1825 | 351 | 872 |
| ice\_t | 1760 | 360 | 945 |

(b) A tibble: 12 × 3

| sample | phase | n\_cells |
| --- | --- | --- |
| <chr> | <chr> | <int> |
| 37c\_no\_t | G1 | 2164 |
| 37c\_no\_t | G2M | 334 |
| 37c\_no\_t | S | 375 |
| 37c\_t | G1 | 1799 |
| 37c\_t | G2M | 608 |
| 37c\_t | S | 1173 |
| ice\_no\_t | G1 | 1825 |
| ice\_no\_t | G2M | 351 |
| ice\_no\_t | S | 872 |
| ice\_t | G1 | 1760 |
| ice\_t | G2M | 360 |
| ice\_t | S | 945 |

```
ggplot(df, aes(fill=phase, y=n_cells, x=sample)) + 
    geom_bar(position="fill", stat="identity") +
    scale_fill_viridis(discrete = T, option = "cividis") +
    ylab("") +
    xlab("Condition")
ggsave(last_plot(), filename = "plots/cc_phase_distribution_per_condition.svg", device = "svg", units = "in", width = 6, height = 6)
```

# Compare ice conditions

```
WhichCells(sobj, expr = sample == "ice_t" & Phase == "S") %>% length
```

945

```
Idents(sobj) <- "sample"
DefaultAssay(sobj) <- "RNA"
degs <- FindMarkers(
    sobj,
    ident.1 = "ice_t",
    ident.2 = "ice_no_t",
    assay = "RNA",
    features = VariableFeatures(sobj)
) %>% arrange(desc(avg_log2FC))
degs
```

A data.frame: 3 × 5

|  | p\_val | avg\_log2FC | pct.1 | pct.2 | p\_val\_adj |
| --- | --- | --- | --- | --- | --- |
|  | <dbl> | <dbl> | <dbl> | <dbl> | <dbl> |
| C77080 | 0.21988781 | -0.2606570 | 0.285 | 0.293 | 1 |
| Ighm | 0.05580379 | -0.6127761 | 0.530 | 0.536 | 1 |
| Igkc | 0.32788650 | -0.6539980 | 0.457 | 0.464 | 1 |

So, only three DEGs, with insignificant p-values. Should probably be a good argument for treating them as one for this.

```
get_degs_cc_wise <- \(idents = "sample", group_1 = "ice_t", group_2 = c("ice_t", "ice_no_t"), cc_phase = "S") {
    Idents(sobj) <- idents
    DefaultAssay(sobj) <- "RNA"

    # extract cell groups:
    cells_1 = WhichCells(sobj, expr = sample %in% group_1 & Phase == cc_phase)
    cells_2 = WhichCells(sobj, expr = sample %in% group_2 & Phase == cc_phase)
    foo_1 <- table(sobj[[]][cells_1,c("sample", "Phase")]) %>% as.data.frame.matrix
    foo_2 <- table(sobj[[]][cells_2,c("sample", "Phase")]) %>% as.data.frame.matrix

    # double-check that the correct cell groups are extracted,
    # will throw an error and fail if they are not correct:
    stopifnot(phase_distributions[group_2, cc_phase] == foo_2[group_2, cc_phase])

    degs <- FindMarkers(
        sobj[["RNA"]],
        cells.1 = cells_1,
        cells.2 = cells_2,
        features = VariableFeatures(sobj)
    ) %>% arrange(desc(avg_log2FC))
    degs %>% return
}
```

```
get_degs_cc_wise(group_1 = "ice_t", group_2 = "ice_no_t", cc_phase = "S")
get_degs_cc_wise(group_1 = "ice_t", group_2 = "ice_no_t", cc_phase = "G1")
get_degs_cc_wise(group_1 = "ice_t", group_2 = "ice_no_t", cc_phase = "G2M")
```

```
Warning message in FindMarkers.default(object = data.use, slot = data.slot, counts = counts, :
“No features pass logfc.threshold threshold; returning empty data.frame”
Warning message in FindMarkers.default(object = data.use, slot = data.slot, counts = counts, :
“No features pass logfc.threshold threshold; returning empty data.frame”
Warning message in FindMarkers.default(object = data.use, slot = data.slot, counts = counts, :
“No features pass logfc.threshold threshold; returning empty data.frame”
```

A data.frame: 0 × 3

| avg\_log2FC | pct.1 | pct.2 |
| --- | --- | --- |
| <dbl> | <dbl> | <dbl> |

A data.frame: 0 × 3

| avg\_log2FC | pct.1 | pct.2 |
| --- | --- | --- |
| <dbl> | <dbl> | <dbl> |

A data.frame: 0 × 3

| avg\_log2FC | pct.1 | pct.2 |
| --- | --- | --- |
| <dbl> | <dbl> | <dbl> |

So yeah, no DEGs at all doing it phase-wise.

# Compare 37c conditions

```
sobj[[]][["sample"]] %>% unique
```

1. '37c\_no\_t'
2. 'ice\_t'
3. '37c\_t'
4. 'ice\_no\_t'

- 37c\_t vs ice
- 37c\_no\_t vs ice
- 37c\_t vs 37c\_no\_t

```
ice <- c("ice_t", "ice_no_t")
comparisons <- list(
    "37c_t" = list("37c_t", ice),
    "37c_no_t" = list("37c_no_t", ice),
    "37c_t_vs_no_t" = list("37c_t", "37c_no_t")
)
# degs <- list(
#     "37c_t" = "",
#     "37c_no_t" = "",
#     "37c_t_vs_no_t" = ""
# )
comparisons
# degs
```

$`37c\_t`
:   1. '37c\_t'
    2. 1. 'ice\_t'
       2. 'ice\_no\_t'

$`37c\_no\_t`
:   1. '37c\_no\_t'
    2. 1. 'ice\_t'
       2. 'ice\_no\_t'

$`37c\_t\_vs\_no\_t`
:   1. '37c\_t'
    2. '37c\_no\_t'

```
get_degs_cc_wise(group_1 = comparisons[["37c_t"]][[1]], group_2 = comparisons[["37c_t"]][[2]], cc_phase = "S") %>% head
```

A data.frame: 6 × 5

|  | p\_val | avg\_log2FC | pct.1 | pct.2 | p\_val\_adj |
| --- | --- | --- | --- | --- | --- |
|  | <dbl> | <dbl> | <dbl> | <dbl> | <dbl> |
| 4933406J09Rik | 0.000000e+00 | 1.0673711 | 0.882 | 0.111 | 0.000000e+00 |
| Gm40841 | 0.000000e+00 | 0.8333755 | 0.777 | 0.034 | 0.000000e+00 |
| Bcl2l14 | 4.972059e-238 | 0.8190220 | 0.552 | 0.038 | 1.605229e-233 |
| Pex1 | 2.962626e-229 | 0.6778770 | 0.708 | 0.216 | 9.564839e-225 |
| Sh2b2 | 8.265819e-252 | 0.6284175 | 0.713 | 0.173 | 2.668620e-247 |
| Gm28403 | 1.804430e-224 | 0.5924536 | 0.609 | 0.087 | 5.825601e-220 |

```
phase_distributions <- table(sobj[[]][,c("sample", "Phase")]) %>% as.data.frame.matrix #%>% rbind(
phase_distributions
```

A data.frame: 4 × 3

|  | G1 | G2M | S |
| --- | --- | --- | --- |
|  | <int> | <int> | <int> |
| 37c\_no\_t | 2164 | 334 | 375 |
| 37c\_t | 1799 | 608 | 1173 |
| ice\_no\_t | 1825 | 351 | 872 |
| ice\_t | 1760 | 360 | 945 |

```
degs <- lapply(comparisons, FUN = \(comp) {
    lapply(phases, FUN = \(phase) {
        group_1 <- comp[[1]]
        group_2 <- comp[[2]]
        print(group_1)
        print(group_2)
        print(phase)
        cells_1 = WhichCells(sobj, expr = sample %in% group_1 & Phase == phase)
        cells_2 = WhichCells(sobj, expr = sample %in% group_2 & Phase == phase)
        print(length(cells_1))
        print(length(cells_2))
        foo_1 <- table(sobj[[]][cells_1,c("sample", "Phase")]) %>% as.data.frame.matrix
        foo_2 <- table(sobj[[]][cells_2,c("sample", "Phase")]) %>% as.data.frame.matrix
        print(foo_1)
        print(foo_2)
        stopifnot(phase_distributions[group_2, phase] == foo_2[group_2, phase])
        print("------")
    })
})
```

```
[1] "37c_t"
[1] "ice_t"    "ice_no_t"
[1] "G1"
[1] 1799
[1] 3585
        G1
37c_t 1799
           G1
ice_no_t 1825
ice_t    1760
[1] "------"
[1] "37c_t"
[1] "ice_t"    "ice_no_t"
[1] "G2M"
[1] 608
[1] 711
      G2M
37c_t 608
         G2M
ice_no_t 351
ice_t    360
[1] "------"
[1] "37c_t"
[1] "ice_t"    "ice_no_t"
[1] "S"
[1] 1173
[1] 1817
         S
37c_t 1173
           S
ice_no_t 872
ice_t    945
[1] "------"
[1] "37c_no_t"
[1] "ice_t"    "ice_no_t"
[1] "G1"
[1] 2164
[1] 3585
           G1
37c_no_t 2164
           G1
ice_no_t 1825
ice_t    1760
[1] "------"
[1] "37c_no_t"
[1] "ice_t"    "ice_no_t"
[1] "G2M"
[1] 334
[1] 711
         G2M
37c_no_t 334
         G2M
ice_no_t 351
ice_t    360
[1] "------"
[1] "37c_no_t"
[1] "ice_t"    "ice_no_t"
[1] "S"
[1] 375
[1] 1817
           S
37c_no_t 375
           S
ice_no_t 872
ice_t    945
[1] "------"
[1] "37c_t"
[1] "37c_no_t"
[1] "G1"
[1] 1799
[1] 2164
        G1
37c_t 1799
           G1
37c_no_t 2164
[1] "------"
[1] "37c_t"
[1] "37c_no_t"
[1] "G2M"
[1] 608
[1] 334
      G2M
37c_t 608
         G2M
37c_no_t 334
[1] "------"
[1] "37c_t"
[1] "37c_no_t"
[1] "S"
[1] 1173
[1] 375
         S
37c_t 1173
           S
37c_no_t 375
[1] "------"
```

```
degs <- lapply(comparisons, FUN = \(comp) {
    lapply(phases, FUN = \(phase) {
        get_degs_cc_wise(group_1 = comp[[1]], group_2 = comp[[2]], cc_phase = phase)
    })
})
```

```
names(degs)
names(degs[["37c_t"]])
```

1. '37c\_t'
2. '37c\_no\_t'
3. '37c\_t\_vs\_no\_t'

1. 'G1'
2. 'G2M'
3. 'S'

```
degs[["37c_t"]][["S"]] %>% head
degs[["37c_t_vs_no_t"]][["S"]] %>% head
```

A data.frame: 6 × 5

|  | p\_val | avg\_log2FC | pct.1 | pct.2 | p\_val\_adj |
| --- | --- | --- | --- | --- | --- |
|  | <dbl> | <dbl> | <dbl> | <dbl> | <dbl> |
| 4933406J09Rik | 0.000000e+00 | 1.0673711 | 0.882 | 0.111 | 0.000000e+00 |
| Gm40841 | 0.000000e+00 | 0.8333755 | 0.777 | 0.034 | 0.000000e+00 |
| Bcl2l14 | 4.972059e-238 | 0.8190220 | 0.552 | 0.038 | 1.605229e-233 |
| Pex1 | 2.962626e-229 | 0.6778770 | 0.708 | 0.216 | 9.564839e-225 |
| Sh2b2 | 8.265819e-252 | 0.6284175 | 0.713 | 0.173 | 2.668620e-247 |
| Gm28403 | 1.804430e-224 | 0.5924536 | 0.609 | 0.087 | 5.825601e-220 |

A data.frame: 6 × 5

|  | p\_val | avg\_log2FC | pct.1 | pct.2 | p\_val\_adj |
| --- | --- | --- | --- | --- | --- |
|  | <dbl> | <dbl> | <dbl> | <dbl> | <dbl> |
| 4933406J09Rik | 9.915117e-148 | 1.1079427 | 0.882 | 0.061 | 3.201096e-143 |
| Bcl2l14 | 6.146130e-70 | 0.8325183 | 0.552 | 0.016 | 1.984278e-65 |
| Gm40841 | 7.159824e-116 | 0.8210346 | 0.777 | 0.053 | 2.311549e-111 |
| Asah2 | 2.039055e-88 | 0.6908264 | 0.731 | 0.192 | 6.583087e-84 |
| Pex1 | 6.065907e-81 | 0.6889232 | 0.708 | 0.205 | 1.958378e-76 |
| Gm28403 | 5.746071e-72 | 0.6102455 | 0.609 | 0.067 | 1.855119e-67 |

## Save DEG tables

```
write.csv(degs[["37c_t"]][["S"]], file = "plots/1.tsv")
```

```
lapply(names(comparisons), FUN = \(comp) {
    comp_name <- comp
    comp <- comparisons[[comp_name]]
    if (length(comp[[2]] > 1)) comp[[2]] <- str_flatten(comp[[2]], "_")
    # print(">>>>>>>>>>>>>>>>>>>>>>>>>>>>>>>>>>>>>>>")
    # print(comp_name)
    # print(comp)
    lapply(phases, FUN = \(phase) {
        # print(comp)
        # print(phase)
        filename <- str_c(
            "plots/", comp[[1]], "_VS_", comp[[2]], "_phase_", phase, ".tsv"
        )
        print(filename)
        # print(dim(degs[[comp_name]][[phase]]))
        # print(head(degs[[comp_name]][[phase]]))
        # print("------")
        write.csv(degs[[comp_name]][[phase]], file = filename)
        # return(file.exists(filename))
        return(dim(degs[[comp_name]][[phase]]))
    })
})
```

```
[1] "plots/37c_t_VS_ice_t_ice_no_t_phase_G1.tsv"
[1] "plots/37c_t_VS_ice_t_ice_no_t_phase_G2M.tsv"
[1] "plots/37c_t_VS_ice_t_ice_no_t_phase_S.tsv"
[1] "plots/37c_no_t_VS_ice_t_ice_no_t_phase_G1.tsv"
[1] "plots/37c_no_t_VS_ice_t_ice_no_t_phase_G2M.tsv"
[1] "plots/37c_no_t_VS_ice_t_ice_no_t_phase_S.tsv"
[1] "plots/37c_t_VS_37c_no_t_phase_G1.tsv"
[1] "plots/37c_t_VS_37c_no_t_phase_G2M.tsv"
[1] "plots/37c_t_VS_37c_no_t_phase_S.tsv"
```

1. $G1
   :   1. 127
       2. 5

   $G2M
   :   1. 153
       2. 5

   $S
   :   1. 136
       2. 5
2. $G1
   :   1. 181
       2. 5

   $G2M
   :   1. 194
       2. 5

   $S
   :   1. 173
       2. 5
3. $G1
   :   1. 255
       2. 5

   $G2M
   :   1. 268
       2. 5

   $S
   :   1. 303
       2. 5

## Overlapping DEGs (intersection between cell cycle phases)

```
comparisons
```

$`37c\_t`
:   1. '37c\_t'
    2. 1. 'ice\_t'
       2. 'ice\_no\_t'

$`37c\_no\_t`
:   1. '37c\_no\_t'
    2. 1. 'ice\_t'
       2. 'ice\_no\_t'

$`37c\_t\_vs\_no\_t`
:   1. '37c\_t'
    2. '37c\_no\_t'

```
tmp <- lapply(degs[["37c_t"]], rownames)
lapply(tmp, length)
intersect(tmp[["G1"]], tmp[["G2M"]]) %>% length
intersect(tmp[["G1"]], tmp[["S"]]) %>% length
intersect(tmp[["S"]], tmp[["G2M"]]) %>% length

a <- intersect(tmp[["G1"]], tmp[["G2M"]])
b <- intersect(tmp[["G1"]], tmp[["S"]])
c <- intersect(tmp[["S"]], tmp[["G2M"]])

intersect(a, intersect(b, c)) %>% length
intersect(a, intersect(b, c))
```

$G1
:   127

$G2M
:   153

$S
:   136

91

95

106

84

1. '4933406J09Rik'
2. 'Gm40841'
3. 'Sh2b2'
4. 'Bcl2l14'
5. 'Asah2'
6. '4930435F18Rik'
7. 'Gm28403'
8. 'Gm5099'
9. 'Pex1'
10. 'Acot12'
11. 'Gm36431'
12. 'Map3k15'
13. 'Nek10'
14. 'Mctp1'
15. 'Diaph2'
16. 'Tmbim7'
17. 'Hormad2'
18. 'Gna14'
19. 'Pard3b'
20. 'Arhgap15'
21. 'A430010J10Rik'
22. 'Gm30551'
23. '1700109H08Rik'
24. 'Kcnq1ot1'
25. 'Lepr'
26. 'Slc12a8'
27. 'Fgfr2'
28. 'C130071C03Rik'
29. 'Esr1'
30. 'Sugct'
31. 'Ubr2'
32. 'Tgfbr1'
33. 'Pml'
34. 'Lyst'
35. 'Atp10a'
36. 'Dyrk1a'
37. 'Fbxo11'
38. 'Fam111a'
39. 'Tbxas1'
40. 'Comt'
41. 'BC035044'
42. 'Parp8'
43. 'Gm15261'
44. 'Ssh2'
45. 'Dnajc6'
46. '1600010M07Rik'
47. 'Runx1'
48. 'Dock10'
49. 'Angpt1'
50. 'Satb1'
51. 'Hlf'
52. 'Runx2'
53. 'Nfkbia'
54. 'Dleu2'
55. 'Etv6'
56. 'Fut8'
57. 'Inpp5d'
58. 'Rps6ka5'
59. 'Ccnl1'
60. 'Zeb2'
61. 'Eya1'
62. 'Tubb4b'
63. 'St8sia4'
64. 'Btg1'
65. 'Ikzf2'
66. 'Calcrl'
67. 'Adgrl4'
68. 'Samsn1'
69. 'Dusp2'
70. 'Gm4258'
71. 'Fli1'
72. 'Ubc'
73. 'Pde4b'
74. 'Hist1h1e'
75. 'Abhd17b'
76. 'Ikzf1'
77. 'Il12a'
78. 'Myc'
79. 'Fchsd2'
80. 'Meis1'
81. 'Gcnt2'
82. 'Dapp1'
83. 'Slc38a2'
84. 'Zfp36l2'

```
# degs[["37c_t"]][["S"]][ intersect(a, intersect(b, c)), degs[["37c_t"]][["S"]]$avg_log2FC > 0]
# degs[["37c_t"]][["S"]][ intersect(a, intersect(b, c)) & "avg_log2FC" < 0, ]
degs[["37c_t"]][["S"]][ intersect(a, intersect(b, c)), ] %>% dim
degs[["37c_t"]][["S"]][ intersect(a, intersect(b, c)), ] %>% head
```

1. 84
2. 5

A data.frame: 6 × 5

|  | p\_val | avg\_log2FC | pct.1 | pct.2 | p\_val\_adj |
| --- | --- | --- | --- | --- | --- |
|  | <dbl> | <dbl> | <dbl> | <dbl> | <dbl> |
| 4933406J09Rik | 0.000000e+00 | 1.0673711 | 0.882 | 0.111 | 0.000000e+00 |
| Gm40841 | 0.000000e+00 | 0.8333755 | 0.777 | 0.034 | 0.000000e+00 |
| Sh2b2 | 8.265819e-252 | 0.6284175 | 0.713 | 0.173 | 2.668620e-247 |
| Bcl2l14 | 4.972059e-238 | 0.8190220 | 0.552 | 0.038 | 1.605229e-233 |
| Asah2 | 7.906624e-184 | 0.5868040 | 0.731 | 0.332 | 2.552654e-179 |
| 4930435F18Rik | 8.301540e-227 | 0.4689763 | 0.477 | 0.003 | 2.680152e-222 |

```
overlapping_degs <- \(comp = "37c_t") {
    tmp <- lapply(degs[[comp]], rownames)
    overlaps <- intersect( tmp[["G1"]], intersect(tmp[["G2M"]], tmp[["S"]]))
    return(degs[[comp]][["G1"]])
}
```

```
overlaps <- lapply(names(comparisons), FUN = \(comp) overlapping_degs(comp = comp))
names(overlaps) <- names(comparisons)
lapply(overlaps, dim)
lapply(overlaps, head)
```

$`37c\_t`
:   1. 127
    2. 5

$`37c\_no\_t`
:   1. 181
    2. 5

$`37c\_t\_vs\_no\_t`
:   1. 255
    2. 5

$`37c\_t`
:   A data.frame: 6 × 5

    |  | p\_val | avg\_log2FC | pct.1 | pct.2 | p\_val\_adj |
    | --- | --- | --- | --- | --- | --- |
    |  | <dbl> | <dbl> | <dbl> | <dbl> | <dbl> |
    | 4933406J09Rik | 0.000000e+00 | 1.1527444 | 0.844 | 0.095 | 0.000000e+00 |
    | Gm40841 | 0.000000e+00 | 0.9474124 | 0.759 | 0.038 | 0.000000e+00 |
    | Sh2b2 | 0.000000e+00 | 0.7757715 | 0.691 | 0.089 | 0.000000e+00 |
    | Bcl2l14 | 3.508155e-251 | 0.7053306 | 0.419 | 0.063 | 1.132608e-246 |
    | Asah2 | 8.817964e-294 | 0.6699535 | 0.723 | 0.333 | 2.846880e-289 |
    | 4930435F18Rik | 0.000000e+00 | 0.5804143 | 0.481 | 0.004 | 0.000000e+00 |

    $`37c\_no\_t`

    A data.frame: 6 × 5

    |  | p\_val | avg\_log2FC | pct.1 | pct.2 | p\_val\_adj |
    | --- | --- | --- | --- | --- | --- |
    |  | <dbl> | <dbl> | <dbl> | <dbl> | <dbl> |
    | Skil | 0 | 1.3172470 | 0.984 | 0.184 | 0 |
    | Pmepa1 | 0 | 1.3051771 | 0.957 | 0.085 | 0 |
    | Cxcr4 | 0 | 1.1231989 | 0.962 | 0.310 | 0 |
    | Pde10a | 0 | 1.0451602 | 0.730 | 0.033 | 0 |
    | Cdkn1a | 0 | 1.0317940 | 0.858 | 0.064 | 0 |
    | Hes1 | 0 | 0.9735955 | 0.687 | 0.054 | 0 |

    $`37c\_t\_vs\_no\_t`

    A data.frame: 6 × 5

    |  | p\_val | avg\_log2FC | pct.1 | pct.2 | p\_val\_adj |
    | --- | --- | --- | --- | --- | --- |
    |  | <dbl> | <dbl> | <dbl> | <dbl> | <dbl> |
    | 4933406J09Rik | 0.000000e+00 | 1.2142660 | 0.844 | 0.036 | 0.000000e+00 |
    | Gm40841 | 0.000000e+00 | 0.9398040 | 0.759 | 0.045 | 0.000000e+00 |
    | Asah2 | 0.000000e+00 | 0.8466622 | 0.723 | 0.157 | 0.000000e+00 |
    | Bcl2l14 | 3.380196e-216 | 0.7417932 | 0.419 | 0.022 | 1.091296e-211 |
    | Mir99ahg | 9.439248e-217 | 0.6265346 | 0.844 | 0.613 | 3.047461e-212 |
    | Nrxn1 | 0.000000e+00 | 0.5995935 | 0.970 | 0.892 | 0.000000e+00 |

```
lapply(names(comparisons), FUN = \(comp) {
    write.csv(overlaps[[comp]], file = str_c(
        "plots/", "deg_overlap_", comp, ".tsv"
    ))
})
```

1. NULL
2. NULL
3. NULL

---

```
feats <- c(
    degs[["37c_t"]][["G1"]] %>% head(., n = 15) %>% rownames,
    degs[["37c_t"]][["G1"]] %>% tail(., n = 15) %>% rownames
)
p1 <- DotPlot(sobj, features = feats, cols = cols_features) + theme(axis.text.x = element_text(angle = 45, vjust = 1, hjust=1))
feats <- c(
    degs[["37c_t"]][["G2M"]] %>% head(., n = 15) %>% rownames,
    degs[["37c_t"]][["G2M"]] %>% tail(., n = 15) %>% rownames
)
p2 <- DotPlot(sobj, features = feats, cols = cols_features) + theme(axis.text.x = element_text(angle = 45, vjust = 1, hjust=1))
feats <- c(
    degs[["37c_t"]][["S"]] %>% head(., n = 15) %>% rownames,
    degs[["37c_t"]][["S"]] %>% tail(., n = 15) %>% rownames
)
p3 <- DotPlot(sobj, features = feats, cols = cols_features) + theme(axis.text.x = element_text(angle = 45, vjust = 1, hjust=1))
```

```
Warning message:
“Scaling data with a low number of groups may produce misleading results”
Warning message:
“Scaling data with a low number of groups may produce misleading results”
Warning message:
“Scaling data with a low number of groups may produce misleading results”
```

```
plot <- (p1 + grid::textGrob("G1")) / p2 / p3
plot
```

```
ggsave(plot, filename = "plots/1.svg", device = "svg", units = "in", width = 12, height = 12)
```

```
one_dot_plot <- \(comparison = "37c_t", n_genes = 15, phase = "S") {
    feats <- c(
        degs[[comparison]][[phase]] %>% head(., n = n_genes) %>% rownames,
        degs[[comparison]][[phase]] %>% tail(., n = n_genes) %>% rownames
    )
    plot <- DotPlot(sobj, features = feats, cols = cols_features) +
        theme(
            axis.text.x = element_text(angle = 45, vjust = 1, hjust=1)
        ) +
        labs(
            # title = str_c("Comparison ", comparison, ":"),
            subtitle = str_c("Cell cycle phase ", phase, ":"),
            y = "",
            x = ""
            # y = "Condition",
            # x = "DEGs"
        )
    return(plot)
}
one_dot_plot()
```

```
Warning message:
“Scaling data with a low number of groups may produce misleading results”
```

```
comp <- "37c_t"
n_genes <- 15
plot <- one_dot_plot(comparison = comp, phase = "G1") /
one_dot_plot(comparison = comp, phase = "G2M") /
one_dot_plot(comparison = comp, phase = "S") + plot_annotation(
  title = str_c(
    "DEGs between ",
    comparisons[[comp]][[1]],
    " and ",
    "(", str_flatten(comparisons[[comp]][[2]], " + "), ")"
  ),
  subtitle = "",
  caption = str_c(
    "The top ", n_genes, " DEGs for ", comp[[1]],
    " and the top ", n_genes, " DEGs for ", 
    str_flatten(comparisons[[comp]][[2]], " + ")
  )
)
plot
```

```
Warning message:
“Scaling data with a low number of groups may produce misleading results”
Warning message:
“Scaling data with a low number of groups may produce misleading results”
Warning message:
“Scaling data with a low number of groups may produce misleading results”
```

```
ggsave(plot, filename = "plots/1.svg", device = "svg", units = "in", width = 12, height = 12)
```

```
all_phases_dot_plot <- \(comparison = "37c_t", n_genes = 15) {
  plot <- one_dot_plot(comparison = comparison, phase = "G1") /
  one_dot_plot(comparison = comparison, phase = "G2M") /
  one_dot_plot(comparison = comparison, phase = "S") + plot_annotation(
    title = str_c(
      "DEGs between ",
      comparisons[[comparison]][[1]],
      " and ",
      str_flatten(comparisons[[comparison]][[2]], " + ")
    ),
    subtitle = "Cell cycle phase-wise",
    caption = str_c(
      "The top ", n_genes, " DEGs for ", comparisons[[comparison]][[1]],
      " (left) and the top ", n_genes, " DEGs for ", 
      str_flatten(comparisons[[comparison]][[2]], " + "),
      " (right)"
    )
  )
  plot %>% return
}
all_phases_dot_plot()
```

```
Warning message:
“Scaling data with a low number of groups may produce misleading results”
Warning message:
“Scaling data with a low number of groups may produce misleading results”
Warning message:
“Scaling data with a low number of groups may produce misleading results”
```

---

# Collated dot plots

```
plot <- all_phases_dot_plot(comparison = "37c_t")
plot
ggsave(plot, filename = "plots/37c_t.svg", device = "svg", units = "in", width = 12, height = 12)
```

```
Warning message:
“Scaling data with a low number of groups may produce misleading results”
Warning message:
“Scaling data with a low number of groups may produce misleading results”
Warning message:
“Scaling data with a low number of groups may produce misleading results”
```

```
plot <- all_phases_dot_plot(comparison = "37c_no_t")
plot
ggsave(plot, filename = "plots/37c_no_t.svg", device = "svg", units = "in", width = 12, height = 12)
```

```
Warning message:
“Scaling data with a low number of groups may produce misleading results”
Warning message:
“Scaling data with a low number of groups may produce misleading results”
Warning message:
“Scaling data with a low number of groups may produce misleading results”
```

```
plot <- all_phases_dot_plot(comparison = "37c_t_vs_no_t")
plot
ggsave(plot, filename = "plots/37c_t_vs_no_t.svg", device = "svg", units = "in", width = 12, height = 12)
```

```
Warning message:
“Scaling data with a low number of groups may produce misleading results”
Warning message:
“Scaling data with a low number of groups may produce misleading results”
Warning message:
“Scaling data with a low number of groups may produce misleading results”
```

## Dot plots overlapping DEGs

```
one_dot_plot_overlaps <- \(comparison = "37c_t", n_genes = 15) {
    feats <- c(
        overlaps[[comparison]] %>% head(., n = n_genes) %>% rownames,
        overlaps[[comparison]] %>% tail(., n = n_genes) %>% rownames
    )
    plot <- DotPlot(sobj, features = feats, cols = cols_features) +
        theme(
            axis.text.x = element_text(angle = 45, vjust = 1, hjust=1)
        ) +
        labs(
            title = str_c(
                "DEGs between ",
                comparisons[[comparison]][[1]],
                " and ",
                str_flatten(comparisons[[comparison]][[2]], " + ")
            ),
            subtitle = str_c(
                "Overlaps/intersection between cell cycle phase-wise DEG analyses"
            ),
            caption = str_c(
                "The top ", n_genes, " DEGs for ", comparisons[[comparison]][[1]],
                " (left) and the top ", n_genes, " DEGs for ", 
                str_flatten(comparisons[[comparison]][[2]], " + "),
                " (right)"
            ),
            y = "Condition",
            x = "DEGs"
        )
    return(plot)
}
one_dot_plot_overlaps()
```

```
Warning message:
“Scaling data with a low number of groups may produce misleading results”
```

```
names(comparisons)
```

1. '37c\_t'
2. '37c\_no\_t'
3. '37c\_t\_vs\_no\_t'

```
one_dot_plot_overlaps("37c_t")
one_dot_plot_overlaps("37c_t_vs_no_t")
one_dot_plot_overlaps("37c_no_t")
```

```
Warning message:
“Scaling data with a low number of groups may produce misleading results”
Warning message:
“Scaling data with a low number of groups may produce misleading results”
Warning message:
“Scaling data with a low number of groups may produce misleading results”
```

```
lapply(names(overlaps), FUN = \(comp) {
    ggsave(
        one_dot_plot_overlaps(comparison = comp),
        filename = str_c("plots/", "deg_overlap_", comp, ".svg"),
        device = "svg", units = "in", width = 12, height = 5
    )
})
```

```
Warning message:
“Scaling data with a low number of groups may produce misleading results”
Warning message:
“Scaling data with a low number of groups may produce misleading results”
Warning message:
“Scaling data with a low number of groups may produce misleading results”
```

1. 'plots/deg\_overlap\_37c\_t.svg'
2. 'plots/deg\_overlap\_37c\_no\_t.svg'
3. 'plots/deg\_overlap\_37c\_t\_vs\_no\_t.svg'

---

# QC plots

```
sobj[[]] %>% colnames
```

1. 'orig.ident'
2. 'nCount\_RNA'
3. 'nFeature\_RNA'
4. 'nCount\_ADT'
5. 'nFeature\_ADT'
6. 'nCount\_HTO'
7. 'nFeature\_HTO'
8. 'percent.mt'
9. 'hto'
10. 'sample'
11. 'sample\_longname'
12. 'buffer\_treatment'
13. 'incubation\_method'
14. 'S.Score'
15. 'G2M.Score'
16. 'Phase'
17. 'old.ident'
18. 'RNA\_snn\_res.0.8'
19. 'seurat\_clusters'
20. 'stress\_signature1'
21. 'is\_stressed'

```
feats_of_interest <- list(
  "nCount_RNA", "nFeature_RNA", "nCount_ADT", "nCount_HTO", "percent.mt", "stress_signature1"
)
feats_of_interest
```

1. 'nCount\_RNA'
2. 'nFeature\_RNA'
3. 'nCount\_ADT'
4. 'nCount\_HTO'
5. 'percent.mt'
6. 'stress\_signature1'

```
qc_plot <- \(feat = "nCount_RNA") {
    VlnPlot(sobj, features = feat) +
        scale_fill_viridis(discrete = T, option = "plasma")
}
qc_plot()
```

```
plots <- lapply(feats_of_interest, qc_plot)
```

```
names(plots) <- feats_of_interest
```

```
lapply(feats_of_interest, FUN = \(feat) ggsave(plots[[feat]],
    filename = str_c("plots/qc_", feat, ".svg"),
    device = "svg",
    units = "in",
    width = 6,
    height = 6)
)
```

1. 'plots/qc\_nCount\_RNA.svg'
2. 'plots/qc\_nFeature\_RNA.svg'
3. 'plots/qc\_nCount\_ADT.svg'
4. 'plots/qc\_nCount\_HTO.svg'
5. 'plots/qc\_percent.mt.svg'
6. 'plots/qc\_stress\_signature1.svg'

```
plots
```

```
$nCount_RNA

$nFeature_RNA

$nCount_ADT

$nCount_HTO

$percent.mt

$stress_signature1
```

---

# Signatures

```
signature_file <- "../data/raw/Genesets.gmx.txt"
```

```
dirname(signature_file) %>% dir
```

1. 'Annas - Stress GEX analysis'
2. 'cellranger-GRCh38-mm10'
3. 'cellranger-mm10'
4. 'Genesets.gmx.txt'
5. 'HTO-fastqs'
6. 'md5sum.txt'
7. 'RNA-fastqs'
8. 'stress\_signature.tsv'

```
sigs_dirty <- read.delim(signature_file)
sigs_dirty %>% head
```

A data.frame: 6 × 4

|  | Stress.induced.in.HSCs | Aged.HSC.genes\_Flohr.Svendsen.et.al | Young.HSC.genes\_Flohr.Svendsen.et.al | Cell.cycle.in.HSCs |
| --- | --- | --- | --- | --- |
|  | <chr> | <chr> | <chr> | <chr> |
| 1 | na | na | na | na |
| 2 | Egr2 | Ntf3 | Lsp1 | Rpa2 |
| 3 | Atf3 | Mab21l2 | Slc28a2 | Hspa8 |
| 4 | Klf2 | Sbspon | Rfc2 | Dtymk |
| 5 | Nr4a1 | Osmr | Ctss | Sqle |
| 6 | Ptgs2 | Cntn1 | Mlec | Mcm5 |

```
sigs <- list()
```

```
sigs[["cc"]] <- sigs_dirty[[colnames(sigs_dirty)[
    str_detect("cycle", string = colnames(sigs_dirty))]
]]
sigs[["young"]] <- sigs_dirty[[colnames(sigs_dirty)[
    str_detect("Young", string = colnames(sigs_dirty))]
]]
sigs[["aged"]] <- sigs_dirty[[colnames(sigs_dirty)[
    str_detect("Aged", string = colnames(sigs_dirty))]
]]
sigs[["stress"]] <- sigs_dirty[[colnames(sigs_dirty)[
    str_detect("Stress", string = colnames(sigs_dirty))]
]]
sigs
```

$cc
:   1. 'na'
    2. 'Rpa2'
    3. 'Hspa8'
    4. 'Dtymk'
    5. 'Sqle'
    6. 'Mcm5'
    7. 'Stmn1'
    8. 'Dmd'
    9. 'Prmt5'
    10. 'Bcl3'
    11. 'Nasp'
    12. 'Ss18'
    13. 'Mcm3'
    14. 'Orc6'
    15. 'Sfpq'
    16. 'Mybl2'
    17. 'Orc5'
    18. 'Wrn'
    19. 'Pttg1'
    20. 'Ythdc1'
    21. 'Smad3'
    22. 'Mapk14'
    23. 'Amd1'
    24. 'Map3k20'
    25. 'Bub3'
    26. 'Hnrnpd'
    27. 'Kpnb1'
    28. 'Ube2s'
    29. 'Cks1b'
    30. 'Srsf1'
    31. 'E2f4'
    32. 'Ddx39a'
    33. 'Traip'
    34. 'Lbr'
    35. 'H2az2'
    36. 'Polq'
    37. 'Dkc1'
    38. 'Arid4a'
    39. 'Rps6ka5'
    40. 'Cdc7'
    41. 'Slc7a1'
    42. 'Pola2'
    43. 'Prpf4b'
    44. 'H2az1'
    45. 'Abl1'
    46. 'Pafah1b1'
    47. 'Lmnb1'
    48. 'Jpt1'
    49. 'Gspt1'
    50. 'Odf2'
    51. 'Foxn3'
    52. 'Troap'
    53. 'Hus1'
    54. 'Ctcf'
    55. 'Numa1'
    56. 'Cdc27'
    57. 'Top1'
    58. 'Prim2'
    59. 'Pml'
    60. 'Pds5b'
    61. 'Kif5b'
    62. 'Nup50'
    63. 'Xpo1'
    64. 'Notch2'
    65. 'Atrx'
    66. 'Nsd2'
    67. 'Rad21'
    68. 'Tle3'
    69. 'Ezh2'
    70. 'Nup98'
    71. 'Stag1'
    72. 'Tent4a'
    73. 'Cdc25a'
    74. 'Smc1a'
    75. 'Rbl1'
    76. 'Lig3'
    77. 'Tmpo'
    78. 'E2f3'
    79. 'Slc38a1'
    80. 'Rad54l'
    81. 'Suv39h1'
    82. 'Mtf2'
    83. 'Egf'
    84. 'Pura'
    85. 'Rasal2'
    86. 'Mad2l1'
    87. 'Pole'
    88. 'Ccnt1'
    89. 'Smarcc1'
    90. 'Cdkn2c'
    91. 'Cdc25b'
    92. 'Brca2'
    93. 'Exo1'
    94. 'Hira'
    95. 'Dbf4'
    96. 'Cks2'
    97. 'Cdc45'
    98. 'Smc2'
    99. 'Smc4'
    100. 'Pbk'
    101. 'H2ax'
    102. 'Cdc20'
    103. 'Plk4'
    104. 'Ndc80'
    105. 'Tacc3'
    106. 'Cenpa'
    107. 'Kif22'
    108. 'Incenp'
    109. 'Stil'
    110. 'Kif23'
    111. 'Knl1'
    112. 'Racgap1'
    113. 'Top2a'
    114. 'Fbxo5'
    115. 'Cdk1'
    116. 'Ccnf'
    117. 'Ccnb2'
    118. 'Ttk'
    119. 'Aurka'
    120. 'Kif20b'
    121. 'Cdkn3'
    122. 'Mki67'
    123. 'Plk1'
    124. 'Ccna2'
    125. 'Tpx2'
    126. 'Aurkb'
    127. 'Birc5'
    128. 'Nusap1'
    129. 'Kif4'
    130. 'Hmmr'
    131. 'Nek2'
    132. 'Espl1'
    133. 'Bub1'
    134. 'Cenpe'
    135. 'Kif11'
    136. 'Prc1'
    137. 'Kif2c'
    138. 'Cenpf'
    139. 'Ube2c'
    140. ''
    141. ''
    142. ''
    143. ''
    144. ''
    145. ''
    146. ''
    147. ''
    148. ''
    149. ''
    150. ''
    151. ''
    152. ''
    153. ''
    154. ''
    155. ''
    156. ''
    157. ''
    158. ''
    159. ''
    160. ''
    161. ''
    162. ''
    163. ''
    164. ''
    165. ''
    166. ''
    167. ''
    168. ''
    169. ''
    170. ''
    171. ''
    172. ''
    173. ''
    174. ''
    175. ''
    176. ''
    177. ''
    178. ''
    179. ''
    180. ''
    181. ''
    182. ''

$young
:   1. 'na'
    2. 'Lsp1'
    3. 'Slc28a2'
    4. 'Rfc2'
    5. 'Ctss'
    6. 'Mlec'
    7. 'Il15'
    8. 'Anxa2'
    9. 'Syk'
    10. 'Ect2'
    11. 'Mcm5'
    12. 'Antxr2'
    13. 'Mamdc2'
    14. 'Cd34'
    15. 'Arhgap30'
    16. 'Lst1'
    17. 'Mgst1'
    18. 'Col4a2'
    19. 'Tm6sf1'
    20. 'Ebi3'
    21. 'Dnmt3b'
    22. 'Cd37'
    23. 'Plxdc2'
    24. 'Rnase6'
    25. 'Flt3'
    26. 'Sell'
    27. 'Rassf4'
    28. 'Csf2rb'
    29. 'Mcm7'
    30. 'Plac8'
    31. 'Cd86'
    32. 'Hnf4a'
    33. 'Socs2'
    34. 'Il12rb2'
    35. 'Satb1'
    36. 'Nrk'
    37. 'Camk1d'
    38. 'Lgals1'
    39. 'Anxa6'
    40. 'Mmp2'
    41. ''
    42. ''
    43. ''
    44. ''
    45. ''
    46. ''
    47. ''
    48. ''
    49. ''
    50. ''
    51. ''
    52. ''
    53. ''
    54. ''
    55. ''
    56. ''
    57. ''
    58. ''
    59. ''
    60. ''
    61. ''
    62. ''
    63. ''
    64. ''
    65. ''
    66. ''
    67. ''
    68. ''
    69. ''
    70. ''
    71. ''
    72. ''
    73. ''
    74. ''
    75. ''
    76. ''
    77. ''
    78. ''
    79. ''
    80. ''
    81. ''
    82. ''
    83. ''
    84. ''
    85. ''
    86. ''
    87. ''
    88. ''
    89. ''
    90. ''
    91. ''
    92. ''
    93. ''
    94. ''
    95. ''
    96. ''
    97. ''
    98. ''
    99. ''
    100. ''
    101. ''
    102. ''
    103. ''
    104. ''
    105. ''
    106. ''
    107. ''
    108. ''
    109. ''
    110. ''
    111. ''
    112. ''
    113. ''
    114. ''
    115. ''
    116. ''
    117. ''
    118. ''
    119. ''
    120. ''
    121. ''
    122. ''
    123. ''
    124. ''
    125. ''
    126. ''
    127. ''
    128. ''
    129. ''
    130. ''
    131. ''
    132. ''
    133. ''
    134. ''
    135. ''
    136. ''
    137. ''
    138. ''
    139. ''
    140. ''
    141. ''
    142. ''
    143. ''
    144. ''
    145. ''
    146. ''
    147. ''
    148. ''
    149. ''
    150. ''
    151. ''
    152. ''
    153. ''
    154. ''
    155. ''
    156. ''
    157. ''
    158. ''
    159. ''
    160. ''
    161. ''
    162. ''
    163. ''
    164. ''
    165. ''
    166. ''
    167. ''
    168. ''
    169. ''
    170. ''
    171. ''
    172. ''
    173. ''
    174. ''
    175. ''
    176. ''
    177. ''
    178. ''
    179. ''
    180. ''
    181. ''
    182. ''

$aged
:   1. 'na'
    2. 'Ntf3'
    3. 'Mab21l2'
    4. 'Sbspon'
    5. 'Osmr'
    6. 'Cntn1'
    7. 'Gabra4'
    8. 'Gipc2'
    9. 'Clu'
    10. 'Rbpjl'
    11. 'Zg16'
    12. 'Selp'
    13. 'Tdrd9'
    14. 'Mt2'
    15. 'Lrrn1'
    16. 'Clca3a1'
    17. 'Plscr2'
    18. 'Tc2n'
    19. 'Matn4'
    20. 'Fap'
    21. 'Muc1'
    22. 'Ramp2'
    23. 'Tmem215'
    24. 'Trpc1'
    25. 'Chrna7'
    26. 'Rgn'
    27. 'Rorb'
    28. 'Tm4sf1'
    29. 'Wwtr1'
    30. 'Gpr183'
    31. 'Zswim5'
    32. 'C4b'
    33. 'Nupr1'
    34. 'Gstm2'
    35. 'Bmpr1a'
    36. 'Tmem47'
    37. 'Gda'
    38. 'Sult1a1'
    39. 'Tmem56'
    40. 'Mt1'
    41. 'Aspa'
    42. 'Cyb561'
    43. 'Neo1'
    44. 'Adgrg2'
    45. 'Pclo'
    46. 'Gm10419'
    47. 'Klrb1c'
    48. 'Jam2'
    49. 'Maf'
    50. 'Dsg2'
    51. 'Perp'
    52. 'Lpl'
    53. 'Pgr'
    54. 'Ddr1'
    55. 'Clec1a'
    56. 'Dnm3'
    57. 'B3galt1'
    58. 'Meis2'
    59. 'Ptprk'
    60. 'Ehd3'
    61. 'Aldh1a1'
    62. 'Cavin2'
    63. 'Phf11d'
    64. 'Abat'
    65. 'Runx1t1'
    66. 'Amotl2'
    67. 'Acpp'
    68. 'Cd200r4'
    69. 'Kdr'
    70. 'Alcam'
    71. 'Asb4'
    72. 'Cd38'
    73. 'Klhl4'
    74. 'Enpp5'
    75. 'Il1rapl2'
    76. 'Gadd45g'
    77. 'Sfrp1'
    78. 'Zfp36'
    79. 'Nrg4'
    80. 'Cpne8'
    81. 'Fhdc1'
    82. 'Cysltr2'
    83. 'Plcl1'
    84. 'Abca4'
    85. 'Clec1b'
    86. 'Vldlr'
    87. 'Pcdhb16'
    88. 'Hpgds'
    89. 'Abcb1a'
    90. 'Vwf'
    91. 'Ghr'
    92. 'Slc6a15'
    93. 'Mmp14'
    94. 'Klrb1b'
    95. 'Id2'
    96. 'Zfp334'
    97. 'Gem'
    98. 'Ocln'
    99. 'Plek'
    100. 'Ptger4'
    101. 'Thbd'
    102. 'Ldhd'
    103. 'Itgb3'
    104. 'S100a6'
    105. 'Dhrs3'
    106. 'Rhoj'
    107. 'Rdh10'
    108. 'Fhl1'
    109. 'Oxr1'
    110. 'Rorc'
    111. 'Rab34'
    112. 'Cyyr1'
    113. 'Myo1e'
    114. 'Stxbp4'
    115. 'Dennd5b'
    116. 'Ampd3'
    117. 'Stom'
    118. 'Lsr'
    119. 'Cyp26b1'
    120. 'Plscr1'
    121. 'Casp12'
    122. 'Mllt3'
    123. 'Pbx3'
    124. 'Tacstd2'
    125. 'Bcl6'
    126. 'Pdgfd'
    127. 'Egr1'
    128. 'Cytip'
    129. 'Ndrg1'
    130. 'Cd9'
    131. 'Tox'
    132. 'Efna1'
    133. 'Vmp1'
    134. 'Trim47'
    135. 'Tgm2'
    136. 'Arhgap29'
    137. 'Serpinb8'
    138. 'Prcp'
    139. 'Pros1'
    140. 'Prtn3'
    141. 'Nckap1'
    142. 'Phactr1'
    143. 'Tbc1d8'
    144. 'Arhgef28'
    145. 'Slc14a1'
    146. 'Lamp2'
    147. 'Acsl4'
    148. 'Cxcl16'
    149. 'Gpx3'
    150. 'Dhx40'
    151. 'Sema7a'
    152. 'Slamf1'
    153. 'Art4'
    154. 'Jun'
    155. 'Serpinb6a'
    156. 'Fyb'
    157. 'Evc'
    158. 'Kcnip3'
    159. 'Mef2c'
    160. 'Abcb1b'
    161. 'Lpar6'
    162. 'Prnp'
    163. 'Exoc6b'
    164. 'Pla2g4a'
    165. 'Ndn'
    166. 'Npdc1'
    167. 'Plscr4'
    168. 'Tnfsf10'
    169. 'Nt5c3'
    170. 'Ppp1r16b'
    171. 'Gstm1'
    172. 'Gstm7'
    173. 'Mmrn1'
    174. 'Cldn12'
    175. 'Procr'
    176. 'Muc13'
    177. 'Tmem176a'
    178. 'Trpc6'
    179. 'Ly6e'
    180. 'Btg2'
    181. 'Cd63'
    182. 'Tsc22d1'

$stress
:   1. 'na'
    2. 'Egr2'
    3. 'Atf3'
    4. 'Klf2'
    5. 'Nr4a1'
    6. 'Ptgs2'
    7. 'Klf4'
    8. 'Fosb'
    9. 'Btg2'
    10. 'Junb'
    11. 'Nr4a2'
    12. 'Zfp36'
    13. 'Cxcl2'
    14. 'Jun'
    15. 'Klf6'
    16. 'Ccn1'
    17. 'Rhob'
    18. 'Socs3'
    19. 'Ier2'
    20. 'Dusp1'
    21. 'Egr1'
    22. 'Cdkn1a'
    23. 'Ppp1r15a'
    24. 'Phlda1'
    25. 'Maff'
    26. 'Cd69'
    27. 'Dusp5'
    28. 'Hes1'
    29. 'Cebpb'
    30. 'Ier5'
    31. 'Plk2'
    32. 'Gem'
    33. 'Tiparp'
    34. 'Gadd45b'
    35. 'Sgk1'
    36. 'Egr3'
    37. 'Ier3'
    38. 'Dusp2'
    39. 'Per1'
    40. 'Hbegf'
    41. 'Trib1'
    42. 'Lif'
    43. 'Tnfaip3'
    44. 'Fos'
    45. 'Cxcl10'
    46. 'Ccrl2'
    47. 'Il7r'
    48. 'Gadd45a'
    49. 'Plek'
    50. 'Fjx1'
    51. 'Zc3h12a'
    52. 'Bhlhe40'
    53. 'Fosl1'
    54. 'Ccnl1'
    55. 'Btg1'
    56. 'Eif1'
    57. 'Mcl1'
    58. 'Plaur'
    59. 'Sdc4'
    60. 'Areg'
    61. 'Vegfa'
    62. 'Rnf19b'
    63. 'Cxcl5'
    64. 'Panx1'
    65. 'Tnf'
    66. 'Sat1'
    67. 'Phlda2'
    68. 'Gfpt2'
    69. 'Id2'
    70. 'Il1b'
    71. 'Gpr183'
    72. 'Pfkfb3'
    73. 'Spsb1'
    74. 'Plau'
    75. 'Cd83'
    76. 'Ripk2'
    77. 'Btg3'
    78. 'Csf1'
    79. 'Tnfrsf9'
    80. ''
    81. ''
    82. ''
    83. ''
    84. ''
    85. ''
    86. ''
    87. ''
    88. ''
    89. ''
    90. ''
    91. ''
    92. ''
    93. ''
    94. ''
    95. ''
    96. ''
    97. ''
    98. ''
    99. ''
    100. ''
    101. ''
    102. ''
    103. ''
    104. ''
    105. ''
    106. ''
    107. ''
    108. ''
    109. ''
    110. ''
    111. ''
    112. ''
    113. ''
    114. ''
    115. ''
    116. ''
    117. ''
    118. ''
    119. ''
    120. ''
    121. ''
    122. ''
    123. ''
    124. ''
    125. ''
    126. ''
    127. ''
    128. ''
    129. ''
    130. ''
    131. ''
    132. ''
    133. ''
    134. ''
    135. ''
    136. ''
    137. ''
    138. ''
    139. ''
    140. ''
    141. ''
    142. ''
    143. ''
    144. ''
    145. ''
    146. ''
    147. ''
    148. ''
    149. ''
    150. ''
    151. ''
    152. ''
    153. ''
    154. ''
    155. ''
    156. ''
    157. ''
    158. ''
    159. ''
    160. ''
    161. ''
    162. ''
    163. ''
    164. ''
    165. ''
    166. ''
    167. ''
    168. ''
    169. ''
    170. ''
    171. ''
    172. ''
    173. ''
    174. ''
    175. ''
    176. ''
    177. ''
    178. ''
    179. ''
    180. ''
    181. ''
    182. ''

```
saved_names <- names(sigs)
sigs <- lapply(sigs, FUN = \(s) s[s %>% str_length > 0 & s != "na"])
# sigs <- lapply(names(sigs), FUN = \(s) sigs[[s]][sigs[[s]] %>% str_length > 0 & sigs[[s]] != "na"])
# names(sigs) <- saved_names
sigs
```

$cc
:   1. 'Rpa2'
    2. 'Hspa8'
    3. 'Dtymk'
    4. 'Sqle'
    5. 'Mcm5'
    6. 'Stmn1'
    7. 'Dmd'
    8. 'Prmt5'
    9. 'Bcl3'
    10. 'Nasp'
    11. 'Ss18'
    12. 'Mcm3'
    13. 'Orc6'
    14. 'Sfpq'
    15. 'Mybl2'
    16. 'Orc5'
    17. 'Wrn'
    18. 'Pttg1'
    19. 'Ythdc1'
    20. 'Smad3'
    21. 'Mapk14'
    22. 'Amd1'
    23. 'Map3k20'
    24. 'Bub3'
    25. 'Hnrnpd'
    26. 'Kpnb1'
    27. 'Ube2s'
    28. 'Cks1b'
    29. 'Srsf1'
    30. 'E2f4'
    31. 'Ddx39a'
    32. 'Traip'
    33. 'Lbr'
    34. 'H2az2'
    35. 'Polq'
    36. 'Dkc1'
    37. 'Arid4a'
    38. 'Rps6ka5'
    39. 'Cdc7'
    40. 'Slc7a1'
    41. 'Pola2'
    42. 'Prpf4b'
    43. 'H2az1'
    44. 'Abl1'
    45. 'Pafah1b1'
    46. 'Lmnb1'
    47. 'Jpt1'
    48. 'Gspt1'
    49. 'Odf2'
    50. 'Foxn3'
    51. 'Troap'
    52. 'Hus1'
    53. 'Ctcf'
    54. 'Numa1'
    55. 'Cdc27'
    56. 'Top1'
    57. 'Prim2'
    58. 'Pml'
    59. 'Pds5b'
    60. 'Kif5b'
    61. 'Nup50'
    62. 'Xpo1'
    63. 'Notch2'
    64. 'Atrx'
    65. 'Nsd2'
    66. 'Rad21'
    67. 'Tle3'
    68. 'Ezh2'
    69. 'Nup98'
    70. 'Stag1'
    71. 'Tent4a'
    72. 'Cdc25a'
    73. 'Smc1a'
    74. 'Rbl1'
    75. 'Lig3'
    76. 'Tmpo'
    77. 'E2f3'
    78. 'Slc38a1'
    79. 'Rad54l'
    80. 'Suv39h1'
    81. 'Mtf2'
    82. 'Egf'
    83. 'Pura'
    84. 'Rasal2'
    85. 'Mad2l1'
    86. 'Pole'
    87. 'Ccnt1'
    88. 'Smarcc1'
    89. 'Cdkn2c'
    90. 'Cdc25b'
    91. 'Brca2'
    92. 'Exo1'
    93. 'Hira'
    94. 'Dbf4'
    95. 'Cks2'
    96. 'Cdc45'
    97. 'Smc2'
    98. 'Smc4'
    99. 'Pbk'
    100. 'H2ax'
    101. 'Cdc20'
    102. 'Plk4'
    103. 'Ndc80'
    104. 'Tacc3'
    105. 'Cenpa'
    106. 'Kif22'
    107. 'Incenp'
    108. 'Stil'
    109. 'Kif23'
    110. 'Knl1'
    111. 'Racgap1'
    112. 'Top2a'
    113. 'Fbxo5'
    114. 'Cdk1'
    115. 'Ccnf'
    116. 'Ccnb2'
    117. 'Ttk'
    118. 'Aurka'
    119. 'Kif20b'
    120. 'Cdkn3'
    121. 'Mki67'
    122. 'Plk1'
    123. 'Ccna2'
    124. 'Tpx2'
    125. 'Aurkb'
    126. 'Birc5'
    127. 'Nusap1'
    128. 'Kif4'
    129. 'Hmmr'
    130. 'Nek2'
    131. 'Espl1'
    132. 'Bub1'
    133. 'Cenpe'
    134. 'Kif11'
    135. 'Prc1'
    136. 'Kif2c'
    137. 'Cenpf'
    138. 'Ube2c'

$young
:   1. 'Lsp1'
    2. 'Slc28a2'
    3. 'Rfc2'
    4. 'Ctss'
    5. 'Mlec'
    6. 'Il15'
    7. 'Anxa2'
    8. 'Syk'
    9. 'Ect2'
    10. 'Mcm5'
    11. 'Antxr2'
    12. 'Mamdc2'
    13. 'Cd34'
    14. 'Arhgap30'
    15. 'Lst1'
    16. 'Mgst1'
    17. 'Col4a2'
    18. 'Tm6sf1'
    19. 'Ebi3'
    20. 'Dnmt3b'
    21. 'Cd37'
    22. 'Plxdc2'
    23. 'Rnase6'
    24. 'Flt3'
    25. 'Sell'
    26. 'Rassf4'
    27. 'Csf2rb'
    28. 'Mcm7'
    29. 'Plac8'
    30. 'Cd86'
    31. 'Hnf4a'
    32. 'Socs2'
    33. 'Il12rb2'
    34. 'Satb1'
    35. 'Nrk'
    36. 'Camk1d'
    37. 'Lgals1'
    38. 'Anxa6'
    39. 'Mmp2'

$aged
:   1. 'Ntf3'
    2. 'Mab21l2'
    3. 'Sbspon'
    4. 'Osmr'
    5. 'Cntn1'
    6. 'Gabra4'
    7. 'Gipc2'
    8. 'Clu'
    9. 'Rbpjl'
    10. 'Zg16'
    11. 'Selp'
    12. 'Tdrd9'
    13. 'Mt2'
    14. 'Lrrn1'
    15. 'Clca3a1'
    16. 'Plscr2'
    17. 'Tc2n'
    18. 'Matn4'
    19. 'Fap'
    20. 'Muc1'
    21. 'Ramp2'
    22. 'Tmem215'
    23. 'Trpc1'
    24. 'Chrna7'
    25. 'Rgn'
    26. 'Rorb'
    27. 'Tm4sf1'
    28. 'Wwtr1'
    29. 'Gpr183'
    30. 'Zswim5'
    31. 'C4b'
    32. 'Nupr1'
    33. 'Gstm2'
    34. 'Bmpr1a'
    35. 'Tmem47'
    36. 'Gda'
    37. 'Sult1a1'
    38. 'Tmem56'
    39. 'Mt1'
    40. 'Aspa'
    41. 'Cyb561'
    42. 'Neo1'
    43. 'Adgrg2'
    44. 'Pclo'
    45. 'Gm10419'
    46. 'Klrb1c'
    47. 'Jam2'
    48. 'Maf'
    49. 'Dsg2'
    50. 'Perp'
    51. 'Lpl'
    52. 'Pgr'
    53. 'Ddr1'
    54. 'Clec1a'
    55. 'Dnm3'
    56. 'B3galt1'
    57. 'Meis2'
    58. 'Ptprk'
    59. 'Ehd3'
    60. 'Aldh1a1'
    61. 'Cavin2'
    62. 'Phf11d'
    63. 'Abat'
    64. 'Runx1t1'
    65. 'Amotl2'
    66. 'Acpp'
    67. 'Cd200r4'
    68. 'Kdr'
    69. 'Alcam'
    70. 'Asb4'
    71. 'Cd38'
    72. 'Klhl4'
    73. 'Enpp5'
    74. 'Il1rapl2'
    75. 'Gadd45g'
    76. 'Sfrp1'
    77. 'Zfp36'
    78. 'Nrg4'
    79. 'Cpne8'
    80. 'Fhdc1'
    81. 'Cysltr2'
    82. 'Plcl1'
    83. 'Abca4'
    84. 'Clec1b'
    85. 'Vldlr'
    86. 'Pcdhb16'
    87. 'Hpgds'
    88. 'Abcb1a'
    89. 'Vwf'
    90. 'Ghr'
    91. 'Slc6a15'
    92. 'Mmp14'
    93. 'Klrb1b'
    94. 'Id2'
    95. 'Zfp334'
    96. 'Gem'
    97. 'Ocln'
    98. 'Plek'
    99. 'Ptger4'
    100. 'Thbd'
    101. 'Ldhd'
    102. 'Itgb3'
    103. 'S100a6'
    104. 'Dhrs3'
    105. 'Rhoj'
    106. 'Rdh10'
    107. 'Fhl1'
    108. 'Oxr1'
    109. 'Rorc'
    110. 'Rab34'
    111. 'Cyyr1'
    112. 'Myo1e'
    113. 'Stxbp4'
    114. 'Dennd5b'
    115. 'Ampd3'
    116. 'Stom'
    117. 'Lsr'
    118. 'Cyp26b1'
    119. 'Plscr1'
    120. 'Casp12'
    121. 'Mllt3'
    122. 'Pbx3'
    123. 'Tacstd2'
    124. 'Bcl6'
    125. 'Pdgfd'
    126. 'Egr1'
    127. 'Cytip'
    128. 'Ndrg1'
    129. 'Cd9'
    130. 'Tox'
    131. 'Efna1'
    132. 'Vmp1'
    133. 'Trim47'
    134. 'Tgm2'
    135. 'Arhgap29'
    136. 'Serpinb8'
    137. 'Prcp'
    138. 'Pros1'
    139. 'Prtn3'
    140. 'Nckap1'
    141. 'Phactr1'
    142. 'Tbc1d8'
    143. 'Arhgef28'
    144. 'Slc14a1'
    145. 'Lamp2'
    146. 'Acsl4'
    147. 'Cxcl16'
    148. 'Gpx3'
    149. 'Dhx40'
    150. 'Sema7a'
    151. 'Slamf1'
    152. 'Art4'
    153. 'Jun'
    154. 'Serpinb6a'
    155. 'Fyb'
    156. 'Evc'
    157. 'Kcnip3'
    158. 'Mef2c'
    159. 'Abcb1b'
    160. 'Lpar6'
    161. 'Prnp'
    162. 'Exoc6b'
    163. 'Pla2g4a'
    164. 'Ndn'
    165. 'Npdc1'
    166. 'Plscr4'
    167. 'Tnfsf10'
    168. 'Nt5c3'
    169. 'Ppp1r16b'
    170. 'Gstm1'
    171. 'Gstm7'
    172. 'Mmrn1'
    173. 'Cldn12'
    174. 'Procr'
    175. 'Muc13'
    176. 'Tmem176a'
    177. 'Trpc6'
    178. 'Ly6e'
    179. 'Btg2'
    180. 'Cd63'
    181. 'Tsc22d1'

$stress
:   1. 'Egr2'
    2. 'Atf3'
    3. 'Klf2'
    4. 'Nr4a1'
    5. 'Ptgs2'
    6. 'Klf4'
    7. 'Fosb'
    8. 'Btg2'
    9. 'Junb'
    10. 'Nr4a2'
    11. 'Zfp36'
    12. 'Cxcl2'
    13. 'Jun'
    14. 'Klf6'
    15. 'Ccn1'
    16. 'Rhob'
    17. 'Socs3'
    18. 'Ier2'
    19. 'Dusp1'
    20. 'Egr1'
    21. 'Cdkn1a'
    22. 'Ppp1r15a'
    23. 'Phlda1'
    24. 'Maff'
    25. 'Cd69'
    26. 'Dusp5'
    27. 'Hes1'
    28. 'Cebpb'
    29. 'Ier5'
    30. 'Plk2'
    31. 'Gem'
    32. 'Tiparp'
    33. 'Gadd45b'
    34. 'Sgk1'
    35. 'Egr3'
    36. 'Ier3'
    37. 'Dusp2'
    38. 'Per1'
    39. 'Hbegf'
    40. 'Trib1'
    41. 'Lif'
    42. 'Tnfaip3'
    43. 'Fos'
    44. 'Cxcl10'
    45. 'Ccrl2'
    46. 'Il7r'
    47. 'Gadd45a'
    48. 'Plek'
    49. 'Fjx1'
    50. 'Zc3h12a'
    51. 'Bhlhe40'
    52. 'Fosl1'
    53. 'Ccnl1'
    54. 'Btg1'
    55. 'Eif1'
    56. 'Mcl1'
    57. 'Plaur'
    58. 'Sdc4'
    59. 'Areg'
    60. 'Vegfa'
    61. 'Rnf19b'
    62. 'Cxcl5'
    63. 'Panx1'
    64. 'Tnf'
    65. 'Sat1'
    66. 'Phlda2'
    67. 'Gfpt2'
    68. 'Id2'
    69. 'Il1b'
    70. 'Gpr183'
    71. 'Pfkfb3'
    72. 'Spsb1'
    73. 'Plau'
    74. 'Cd83'
    75. 'Ripk2'
    76. 'Btg3'
    77. 'Csf1'
    78. 'Tnfrsf9'

```
lapply(sigs, FUN = \(s) s %in% rownames(sobj) %>% table)
# lapply(sigs, FUN = \(s) lapply(s,
#     FUN = \(gene) str_detect(pattern = gene, string = rownames(sobj)) %>% sum
#     ) %>% table
# )
```

```
$cc
.
FALSE  TRUE 
    4   134 

$young
.
TRUE 
  39 

$aged
.
TRUE 
 181 

$stress
.
TRUE 
  78
```

```
s <- "cc"
new <- AddModuleScore(sobj, features = sigs, name = names(sigs))
new
```

```
Warning message:
“The following features are not present in the object: Ddx39a, H2az2, H2az1, H2ax, not searching for symbol synonyms”
```

```
An object of class Seurat 
32293 features across 12566 samples within 3 assays 
Active assay: RNA (32285 features, 2000 variable features)
 2 other assays present: ADT, HTO
 2 dimensional reductions calculated: pca, umap
```

```
new[[]] %>% head
```

A data.frame: 6 × 25

|  | orig.ident | nCount\_RNA | nFeature\_RNA | nCount\_ADT | nFeature\_ADT | nCount\_HTO | nFeature\_HTO | percent.mt | hto | sample | ⋯ | Phase | old.ident | RNA\_snn\_res.0.8 | seurat\_clusters | stress\_signature1 | is\_stressed | cc1 | young2 | aged3 | stress4 |
| --- | --- | --- | --- | --- | --- | --- | --- | --- | --- | --- | --- | --- | --- | --- | --- | --- | --- | --- | --- | --- | --- |
|  | <chr> | <dbl> | <int> | <dbl> | <int> | <dbl> | <int> | <dbl> | <chr> | <chr> | ⋯ | <chr> | <fct> | <fct> | <fct> | <dbl> | <chr> | <dbl> | <dbl> | <dbl> | <dbl> |
| AAACCCAAGAGACAAG-1 | DB\_AKC\_citeseq | 11976 | 3437 | 128 | 4 | 32 | 3 | 2.104208 | HTO2 | 37c\_no\_t | ⋯ | G1 | HTO2 | 0 | 0 | 0.19599848 | stressed | 0.03920330 | -0.149312883 | -0.01416662 | 0.19209645 |
| AAACCCAAGAGTGAAG-1 | DB\_AKC\_citeseq | 21028 | 5475 | 54 | 4 | 108 | 4 | 2.425338 | HTO3 | ice\_t | ⋯ | S | HTO3 | 1 | 1 | -0.10710847 | not\_stressed | 0.10166306 | -0.142437271 | -0.04962019 | -0.10621570 |
| AAACCCAAGCGAAACC-1 | DB\_AKC\_citeseq | 10688 | 2813 | 56 | 3 | 87 | 2 | 4.519087 | HTO4 | 37c\_t | ⋯ | G1 | HTO4 | 5 | 5 | -0.04234327 | not\_stressed | -0.06231414 | 0.005144908 | -0.05448123 | -0.04383476 |
| AAACCCAAGGTAAAGG-1 | DB\_AKC\_citeseq | 10627 | 3677 | 68 | 4 | 79 | 4 | 2.888868 | HTO1 | ice\_no\_t | ⋯ | G1 | HTO1 | 2 | 2 | -0.04919840 | not\_stressed | -0.04869483 | -0.107309223 | -0.04362710 | -0.05414716 |
| AAACCCAAGGTCTACT-1 | DB\_AKC\_citeseq | 16865 | 3915 | 106 | 4 | 99 | 2 | 2.514082 | HTO4 | 37c\_t | ⋯ | S | HTO4 | 7 | 7 | -0.08366769 | not\_stressed | 0.15123558 | -0.007321353 | -0.07141288 | -0.08157760 |
| AAACCCAAGTCGGCCT-1 | DB\_AKC\_citeseq | 13939 | 4192 | 60 | 3 | 75 | 2 | 3.285745 | HTO3 | ice\_t | ⋯ | G1 | HTO3 | 2 | 2 | -0.09381362 | not\_stressed | -0.09269975 | -0.104607233 | 0.03109269 | -0.09134155 |

```
cols <- c("gray", "lightcoral", "red")
```

```
cc <- FeaturePlot(new, features = "cc1", coord.fixed = T, order = T, cols = cols) +
    ggtitle("Cell cycle signature")
young <- FeaturePlot(new, features = "young2", coord.fixed = T, order = T, cols = cols) +
    ggtitle("Young signature")
aged <- FeaturePlot(new, features = "aged3", coord.fixed = T, order = T, cols = cols) +
    ggtitle("Aged signature")
cc
young
aged
```

```
ggsave(cc, filename = str_c("plots/signature_", "cc", ".svg"),
    device = "svg", units = "in", width = 6, height = 6)
ggsave(young, filename = str_c("plots/signature_", "young", ".svg"),
    device = "svg", units = "in", width = 6, height = 6)
ggsave(aged, filename = str_c("plots/signature_", "aged", ".svg"),
    device = "svg", units = "in", width = 6, height = 6)
```
