# Supplementary material for: Implications of stress-induced gene expression for hematopoietic stem cell aging studies
Source: Nat Aging. 2024 Jan 16;4(2):177–84. doi: 10.1038/s43587-023-00558-z (PMC10878961; doi:10.1038/s43587-023-00558-z)
Supplement: Supplementary file 7 — The code for the bioinformatics analysis. [file 43587_2023_558_MOESM7_ESM.tar › notebooks/plots.r.html]

IER DEGs


## Table of contents

- Summary and overview
- Setup
- Visualization of dataset with conditions, for reference
- Old DEG analysis
  - DEGs visualized on UMAP
    - G1
    - G2M
    - S
- New DEG analysis
  - 37c with tript *vs* ice with tript
    - DEG table
    - DEG visualizations

## Other Formats

- PDF

# IER DEGs

Author

Rasmus Olofzon

# Summary and overview

Highlights:

- UMAPs with sample and cell cycle phase visualized: Figure 1
- Metadata for dataset: Table 1
- Cell cycle phase classification distribution per condition: Table 2
- Previously performed DEG analysis, comparisons shown here explained in this diagram: Figure 2
  - For **G1** phase: Figure 3
  - For **G2M** phase: Figure 4
  - For **S** phase: Figure 5
- New DEG analysis (explanatory diagram in Figure 6)
  - Table with DEGs: Table 3
  - Figure with top DEGs shown: Figure 7

# Setup

Code

```
library(Seurat)
library(stringr)
library(dplyr)
library(ggplot2)
```

Code

```
sobj <- readRDS("../data/processed/seurat_object_w_stress_sig.rds")
```

Code

```
sobj
```

```
An object of class Seurat 
32293 features across 12566 samples within 3 assays 
Active assay: RNA (32285 features, 2000 variable features)
 2 other assays present: ADT, HTO
 2 dimensional reductions calculated: pca, umap
```

Code

```
cols_features <- c("moccasin", "darkslategray")
```

Code

```
phases <- list("G1", "G2M", "S")
names(phases) <- phases
deg_files <- lapply(phases, FUN = \(x) str_c("../data/processed/deg/deg_", x, "_ice_t_vs_37c_t.tsv"))
# deg_files
```

Code

```
degs <- lapply(phases, FUN = \(phase) read.table(deg_files[[phase]]))
```

---

# Visualization of dataset with conditions, for reference

Metadata:

Code

```
sobj[[]] %>% head
```

Table 1: Metadata for dataset

|  | orig.ident | nCount\_RNA | nFeature\_RNA | nCount\_ADT | nFeature\_ADT | nCount\_HTO | nFeature\_HTO | percent.mt | hto | sample | ⋯ | buffer\_treatment | incubation\_method | S.Score | G2M.Score | Phase | old.ident | RNA\_snn\_res.0.8 | seurat\_clusters | stress\_signature1 | is\_stressed |
| --- | --- | --- | --- | --- | --- | --- | --- | --- | --- | --- | --- | --- | --- | --- | --- | --- | --- | --- | --- | --- | --- |
|  | <chr> | <dbl> | <int> | <dbl> | <int> | <dbl> | <int> | <dbl> | <chr> | <chr> | ⋯ | <chr> | <chr> | <dbl> | <dbl> | <chr> | <fct> | <fct> | <fct> | <dbl> | <chr> |
| AAACCCAAGAGACAAG-1 | DB\_AKC\_citeseq | 11976 | 3437 | 128 | 4 | 32 | 3 | 2.104208 | HTO2 | 37c\_no\_t | ⋯ | no\_triptolide | 37c | -0.2740383 | -0.19990412 | G1 | HTO2 | 0 | 0 | 0.19599848 | stressed |
| AAACCCAAGAGTGAAG-1 | DB\_AKC\_citeseq | 21028 | 5475 | 54 | 4 | 108 | 4 | 2.425338 | HTO3 | ice\_t | ⋯ | with\_triptolide | ice | 0.2386460 | -0.01205648 | S | HTO3 | 1 | 1 | -0.10710847 | not\_stressed |
| AAACCCAAGCGAAACC-1 | DB\_AKC\_citeseq | 10688 | 2813 | 56 | 3 | 87 | 2 | 4.519087 | HTO4 | 37c\_t | ⋯ | with\_triptolide | 37c | -0.1966232 | -0.14132145 | G1 | HTO4 | 5 | 5 | -0.04234327 | not\_stressed |
| AAACCCAAGGTAAAGG-1 | DB\_AKC\_citeseq | 10627 | 3677 | 68 | 4 | 79 | 4 | 2.888868 | HTO1 | ice\_no\_t | ⋯ | no\_triptolide | ice | -0.1555948 | -0.21232288 | G1 | HTO1 | 2 | 2 | -0.04919840 | not\_stressed |
| AAACCCAAGGTCTACT-1 | DB\_AKC\_citeseq | 16865 | 3915 | 106 | 4 | 99 | 2 | 2.514082 | HTO4 | 37c\_t | ⋯ | with\_triptolide | 37c | 0.2038750 | 0.17149954 | S | HTO4 | 7 | 7 | -0.08366769 | not\_stressed |
| AAACCCAAGTCGGCCT-1 | DB\_AKC\_citeseq | 13939 | 4192 | 60 | 3 | 75 | 2 | 3.285745 | HTO3 | ice\_t | ⋯ | with\_triptolide | ice | -0.3008664 | -0.25975436 | G1 | HTO3 | 2 | 2 | -0.09381362 | not\_stressed |

Code

```
DimPlot(sobj, group.by = "sample") + coord_fixed()
DimPlot(sobj, group.by = "Phase") + coord_fixed()
```

Figure 1: UMAP coloured on metadata

(a) Coloured on condition/sample

(b) Coloured on cell cycle phases

Code

```
table(sobj[[]][,c("sample", "Phase")]) %>% as.data.frame.matrix
```

Table 2: Cell cycle phase classification distribution per condition

|  | G1 | G2M | S |
| --- | --- | --- | --- |
|  | <int> | <int> | <int> |
| 37c\_no\_t | 2164 | 334 | 375 |
| 37c\_t | 1799 | 608 | 1173 |
| ice\_no\_t | 1825 | 351 | 872 |
| ice\_t | 1760 | 360 | 945 |

---

# Old DEG analysis

From former analysis we performed differential gene expression testing cell cycle phase-wise. The comparison was between conditions/samples. This yielded the following set of files:

Code

```
system("ls ../data/processed/deg/", intern = T)
```

1. 'Archive.zip'
2. 'deg\_G1\_37c\_no\_t\_vs\_37c\_t.tsv'
3. 'deg\_G1\_ice\_no\_t\_vs\_37c\_no\_t.tsv'
4. 'deg\_G1\_ice\_no\_t\_vs\_ice\_t.tsv'
5. 'deg\_G1\_ice\_t\_vs\_37c\_t.tsv'
6. 'deg\_G1\_ice\_vs\_37c\_no\_t.tsv'
7. 'deg\_G1\_ice\_vs\_37c\_t.tsv'
8. 'deg\_G2M\_37c\_no\_t\_vs\_37c\_t.tsv'
9. 'deg\_G2M\_ice\_no\_t\_vs\_37c\_no\_t.tsv'
10. 'deg\_G2M\_ice\_no\_t\_vs\_ice\_t.tsv'
11. 'deg\_G2M\_ice\_t\_vs\_37c\_t.tsv'
12. 'deg\_G2M\_ice\_vs\_37c\_no\_t.tsv'
13. 'deg\_G2M\_ice\_vs\_37c\_t.tsv'
14. 'deg\_S\_37c\_no\_t\_vs\_37c\_t.tsv'
15. 'deg\_S\_ice\_no\_t\_vs\_37c\_no\_t.tsv'
16. 'deg\_S\_ice\_no\_t\_vs\_ice\_t.tsv'
17. 'deg\_S\_ice\_t\_vs\_37c\_t.tsv'
18. 'deg\_S\_ice\_vs\_37c\_no\_t.tsv'
19. 'deg\_S\_ice\_vs\_37c\_t.tsv'

Figure 2: Schema for (old) DEG comparisons to follow.

```
flowchart TB
  subgraph ice with triptolide
  G1_ice[G1]
  G2M_ice[G2M]
  S_ice[S]
  end
  subgraph 37c with triptolide
  G1_37c[G1] --- G1_ice
  G2M_37c[G2M] --- G2M_ice
  S_37c[S] --- S_ice
  end
```

For each file, both up- and down-regulated genes are included. Here we have loaded the comparison between 37c with triptolide and ice with triptolide (see Figure 2). So first we have the top 6 ice-specific, for the G1 phase:

```
degs[["G1"]] %>% head
```

A data.frame: 6 × 5

|  | p\_val | avg\_log2FC | pct.1 | pct.2 | p\_val\_adj |
| --- | --- | --- | --- | --- | --- |
|  | <dbl> | <dbl> | <dbl> | <dbl> | <dbl> |
| Zfp36l2 | 0 | 1.958553 | 0.990 | 0.439 | 0 |
| Mir142hg | 0 | 1.894032 | 0.962 | 0.245 | 0 |
| Txnip | 0 | 1.855793 | 0.945 | 0.183 | 0 |
| Sox4 | 0 | 1.635571 | 0.945 | 0.458 | 0 |
| Dapp1 | 0 | 1.552930 | 0.966 | 0.568 | 0 |
| Gcnt2 | 0 | 1.479956 | 0.959 | 0.586 | 0 |

And the top 6 37c-specific, for the G1 phase as well:

```
degs[["G1"]] %>% tail
```

A data.frame: 6 × 5

|  | p\_val | avg\_log2FC | pct.1 | pct.2 | p\_val\_adj |
| --- | --- | --- | --- | --- | --- |
|  | <dbl> | <dbl> | <dbl> | <dbl> | <dbl> |
| Sh2b2 | 0.000000e+00 | -1.357543 | 0.093 | 0.691 | 0.000000e+00 |
| A930015D03Rik | 3.628043e-15 | -1.372958 | 0.487 | 0.298 | 1.171314e-10 |
| Asah2 | 7.332960e-212 | -1.375551 | 0.322 | 0.723 | 2.367446e-207 |
| Bcl2l14 | 3.884711e-151 | -1.661921 | 0.061 | 0.419 | 1.254179e-146 |
| Gm40841 | 0.000000e+00 | -1.682257 | 0.040 | 0.759 | 0.000000e+00 |
| 4933406J09Rik | 0.000000e+00 | -2.247251 | 0.101 | 0.844 | 0.000000e+00 |

---

## DEGs visualized on UMAP

### G1

Code

```
FeaturePlot(sobj, features = degs[["G1"]] %>% head %>% rownames, coord.fixed = T, order = T, cols = cols_features)
FeaturePlot(sobj, features = degs[["G1"]] %>% tail %>% rownames, coord.fixed = T, order = T, cols = cols_features)
```

Figure 3: Top DEGs for phase **G1**.

(a) Top 6 DEGs specific to **ice w/ t** condition

(b) Top 6 DEGs specific to **37c w/ t** condition

### G2M

Code

```
FeaturePlot(sobj, features = degs[["G2M"]] %>% head %>% rownames, coord.fixed = T, order = T, cols = cols_features)
FeaturePlot(sobj, features = degs[["G2M"]] %>% tail %>% rownames, coord.fixed = T, order = T, cols = cols_features)
```

Figure 4: Top DEGs for phase **G2M**.

(a) Top 6 DEGs specific to **ice w/ t** condition

(b) Top 6 DEGs specific to **37c w/ t** condition

### S

Code

```
FeaturePlot(sobj, features = degs[["S"]] %>% head %>% rownames, coord.fixed = T, order = T, cols = cols_features)
FeaturePlot(sobj, features = degs[["S"]] %>% tail %>% rownames, coord.fixed = T, order = T, cols = cols_features)
```

Figure 5: Top DEGs for phase **S**.

(a) Top 6 DEGs specific to **ice w/ t** condition

(b) Top 6 DEGs specific to **37c w/ t** condition

---

# New DEG analysis

Specifically done *not* cell cycle phase-wise. Will be done for:

- 37c with tript *VS* ice with tript

Figure 6: Schema for new DEG comparison.

```
flowchart TB
  subgraph ice with triptolide
  ice[G1, G2M, S]
  end
  subgraph 37c with triptolide
  37c[G1, G2M, S] --- ice
  end
```

## 37c with tript *vs* ice with tript

Sample names used in object:

Code

```
sobj[[]][["sample"]] %>% unique
```

1. '37c\_no\_t'
2. 'ice\_t'
3. '37c\_t'
4. 'ice\_no\_t'

```
Idents(sobj) <- "sample"
DefaultAssay(sobj) <- "RNA"
new_degs <- FindMarkers(sobj, ident.1 = "37c_t", ident.2 = "ice_t", assay = "RNA", features = VariableFeatures(sobj)) %>% arrange(desc(avg_log2FC))
```

### DEG table

Code

```
new_degs
```

Table 3: Differentially expressed genes between `37c_t` and `ice_t`. Positive fold change = up in `37c_t`, negative fold change = up in `ice_t`.

|  | p\_val | avg\_log2FC | pct.1 | pct.2 | p\_val\_adj |
| --- | --- | --- | --- | --- | --- |
|  | <dbl> | <dbl> | <dbl> | <dbl> | <dbl> |
| 4933406J09Rik | 0.000000e+00 | 2.0847480 | 0.862 | 0.115 | 0.000000e+00 |
| Bcl2l14 | 0.000000e+00 | 1.6003084 | 0.465 | 0.052 | 0.000000e+00 |
| Gm40841 | 0.000000e+00 | 1.5380264 | 0.771 | 0.039 | 0.000000e+00 |
| A930015D03Rik | 3.476910e-33 | 1.2839138 | 0.339 | 0.552 | 1.122520e-28 |
| Sh2b2 | 0.000000e+00 | 1.2106941 | 0.709 | 0.127 | 0.000000e+00 |
| Asah2 | 0.000000e+00 | 1.2047546 | 0.730 | 0.334 | 0.000000e+00 |
| Arhgap15 | 0.000000e+00 | 1.1174486 | 0.996 | 0.989 | 0.000000e+00 |
| Pex1 | 0.000000e+00 | 1.0954294 | 0.599 | 0.195 | 0.000000e+00 |
| Gm28403 | 0.000000e+00 | 1.0177440 | 0.566 | 0.076 | 0.000000e+00 |
| Mctp1 | 9.482904e-216 | 0.9753501 | 0.951 | 0.923 | 3.061555e-211 |
| Ccser1 | 4.533434e-135 | 0.9561586 | 0.773 | 0.678 | 1.463619e-130 |
| 4930435F18Rik | 0.000000e+00 | 0.8894889 | 0.494 | 0.005 | 0.000000e+00 |
| Diaph3 | 4.369147e-115 | 0.8549220 | 0.699 | 0.592 | 1.410579e-110 |
| Diaph2 | 9.363747e-142 | 0.8476800 | 0.736 | 0.609 | 3.023086e-137 |
| Cep128 | 6.111412e-98 | 0.8312018 | 0.620 | 0.509 | 1.973069e-93 |
| Gm36431 | 0.000000e+00 | 0.8201461 | 0.396 | 0.012 | 0.000000e+00 |
| Tmbim7 | 0.000000e+00 | 0.8201281 | 0.425 | 0.008 | 0.000000e+00 |
| Gm5099 | 0.000000e+00 | 0.7989243 | 0.513 | 0.008 | 0.000000e+00 |
| Pard3b | 2.769102e-149 | 0.7973588 | 0.875 | 0.808 | 8.940047e-145 |
| Kcnq1ot1 | 1.714648e-114 | 0.7565798 | 0.908 | 0.864 | 5.535742e-110 |
| Prkg1 | 4.200709e-63 | 0.7341202 | 0.827 | 0.799 | 1.356199e-58 |
| Acot12 | 0.000000e+00 | 0.7065082 | 0.455 | 0.003 | 0.000000e+00 |
| Fgfr2 | 4.977226e-110 | 0.7027180 | 0.658 | 0.531 | 1.606897e-105 |
| Mir99ahg | 1.173616e-66 | 0.7014709 | 0.816 | 0.826 | 3.789018e-62 |
| Rad51b | 1.169004e-37 | 0.6700331 | 0.572 | 0.502 | 3.774131e-33 |
| Nek10 | 4.344622e-288 | 0.6620993 | 0.373 | 0.011 | 1.402661e-283 |
| Map3k15 | 5.950301e-300 | 0.6444104 | 0.409 | 0.026 | 1.921055e-295 |
| Gna14 | 7.698322e-271 | 0.6443333 | 0.403 | 0.041 | 2.485403e-266 |
| Hormad2 | 1.147156e-251 | 0.6362092 | 0.367 | 0.028 | 3.703594e-247 |
| Esr1 | 1.082358e-81 | 0.6282468 | 0.715 | 0.623 | 3.494394e-77 |
| ⋮ | ⋮ | ⋮ | ⋮ | ⋮ | ⋮ |
| Fam111a | 4.671189e-242 | -0.7086627 | 0.335 | 0.679 | 1.508093e-237 |
| Runx1 | 1.348801e-235 | -0.7112591 | 0.813 | 0.955 | 4.354603e-231 |
| Dleu2 | 0.000000e+00 | -0.7185027 | 0.207 | 0.680 | 0.000000e+00 |
| Rps6ka5 | 2.082905e-307 | -0.7386294 | 0.365 | 0.764 | 6.724660e-303 |
| Zeb2 | 1.804149e-182 | -0.7529491 | 0.468 | 0.754 | 5.824696e-178 |
| Gm4258 | 0.000000e+00 | -0.7675290 | 0.222 | 0.668 | 0.000000e+00 |
| Ltb | 9.087834e-167 | -0.7731711 | 0.309 | 0.636 | 2.934007e-162 |
| Tubb4b | 1.347083e-245 | -0.7773395 | 0.443 | 0.801 | 4.349059e-241 |
| Hist1h1e | 3.536736e-154 | -0.7796415 | 0.264 | 0.619 | 1.141835e-149 |
| Ikzf2 | 5.149385e-243 | -0.7877484 | 0.505 | 0.819 | 1.662479e-238 |
| Calcrl | 2.602565e-237 | -0.7918211 | 0.518 | 0.816 | 8.402380e-233 |
| Fut8 | 2.539314e-214 | -0.8042616 | 0.680 | 0.908 | 8.198174e-210 |
| Abhd17b | 0.000000e+00 | -0.8345477 | 0.186 | 0.717 | 0.000000e+00 |
| Fli1 | 0.000000e+00 | -0.8746406 | 0.603 | 0.916 | 0.000000e+00 |
| Hlf | 3.186349e-205 | -0.8866952 | 0.817 | 0.936 | 1.028713e-200 |
| Samsn1 | 0.000000e+00 | -0.9064422 | 0.785 | 0.970 | 0.000000e+00 |
| Hist1h1b | 1.075679e-12 | -0.9673167 | 0.297 | 0.386 | 3.472828e-08 |
| Igkc | 1.736173e-01 | -0.9861650 | 0.429 | 0.457 | 1.000000e+00 |
| Il12a | 0.000000e+00 | -1.0254985 | 0.091 | 0.550 | 0.000000e+00 |
| Myc | 0.000000e+00 | -1.0260883 | 0.091 | 0.675 | 0.000000e+00 |
| Etv6 | 0.000000e+00 | -1.0842947 | 0.947 | 0.994 | 0.000000e+00 |
| Ikzf1 | 0.000000e+00 | -1.0933579 | 0.591 | 0.927 | 0.000000e+00 |
| Angpt1 | 2.649381e-294 | -1.1610093 | 0.954 | 0.985 | 8.553526e-290 |
| Fchsd2 | 0.000000e+00 | -1.2107342 | 0.522 | 0.946 | 0.000000e+00 |
| Meis1 | 0.000000e+00 | -1.3478146 | 0.690 | 0.969 | 0.000000e+00 |
| Adgrl4 | 0.000000e+00 | -1.3949749 | 0.931 | 0.984 | 0.000000e+00 |
| Slc38a2 | 0.000000e+00 | -1.3967915 | 0.419 | 0.957 | 0.000000e+00 |
| Gcnt2 | 0.000000e+00 | -1.4181788 | 0.590 | 0.958 | 0.000000e+00 |
| Dapp1 | 0.000000e+00 | -1.5052702 | 0.630 | 0.977 | 0.000000e+00 |
| Zfp36l2 | 0.000000e+00 | -1.7229168 | 0.546 | 0.992 | 0.000000e+00 |

### DEG visualizations

Code

```
FeaturePlot(sobj, features = new_degs %>% head %>% rownames, coord.fixed = T, order = T, cols = cols_features)
FeaturePlot(sobj, features = new_degs %>% tail %>% rownames, coord.fixed = T, order = T, cols = cols_features)
```

Figure 7: Top DEGs between 37c with triptolide and ice with triptolide.

(a) Top 6 DEGs specific to **`37c_t`** condition

(b) Top 6 DEGs specific to **`ice_t`** condition
