# Supplementary figures and images for: Implications of stress-induced gene expression for hematopoietic stem cell aging studies
Source: Nat Aging. 2024 Jan 16;4(2):177–84. doi: 10.1038/s43587-023-00558-z (PMC10878961; doi:10.1038/s43587-023-00558-z)

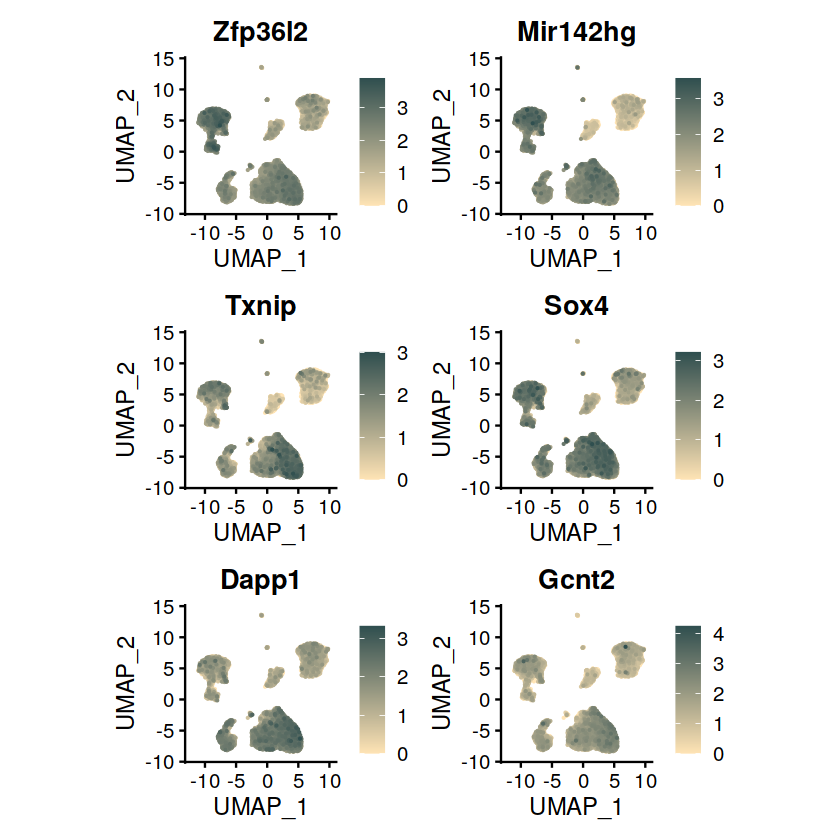

Supplement: Supplementary file 7 — The code for the bioinformatics analysis. [file 43587_2023_558_MOESM7_ESM.tar › notebooks/plots.r_files/figure-markdown_strict/fig-deg-old-g1-output-1.png]

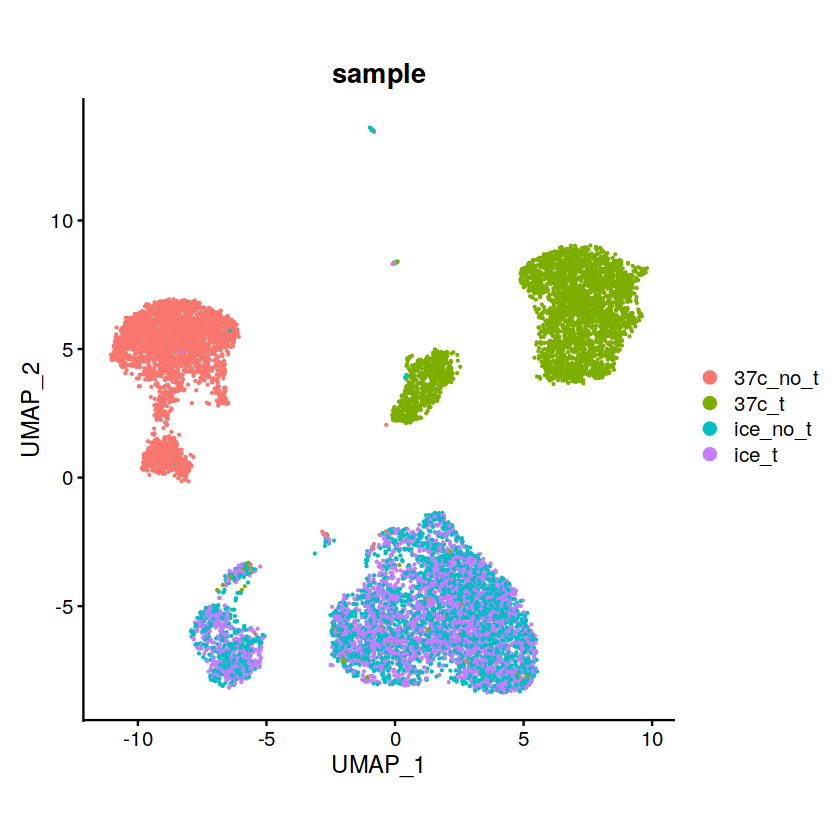

Supplement: Supplementary file 7 — The code for the bioinformatics analysis. [file 43587_2023_558_MOESM7_ESM.tar › notebooks/sc_qc.r_files/figure-markdown_strict/fig-dimplots-output-1.png]

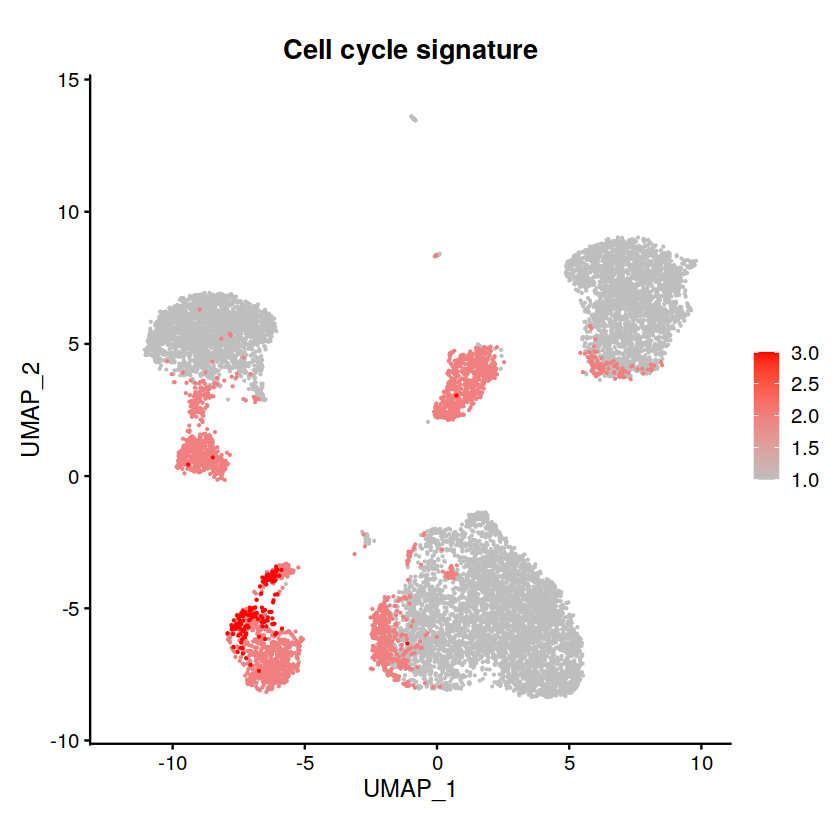

Supplement: Supplementary file 7 — The code for the bioinformatics analysis. [file 43587_2023_558_MOESM7_ESM.tar › notebooks/sc_qc.r_files/figure-markdown_strict/cell-66-output-1.png]

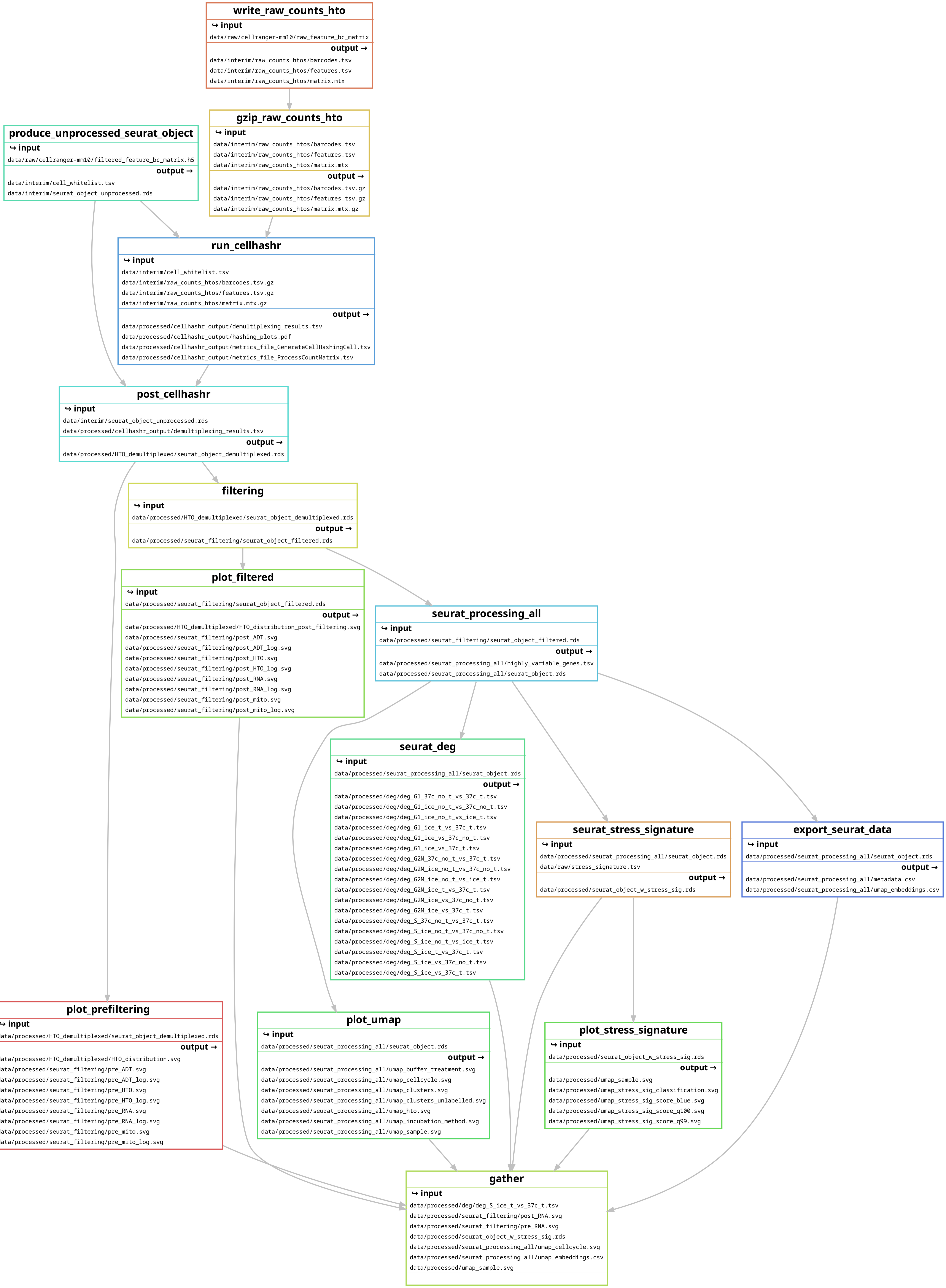

Supplement: Supplementary file 7 — The code for the bioinformatics analysis. [file 43587_2023_558_MOESM7_ESM.tar › smk_filegraph.pdf]

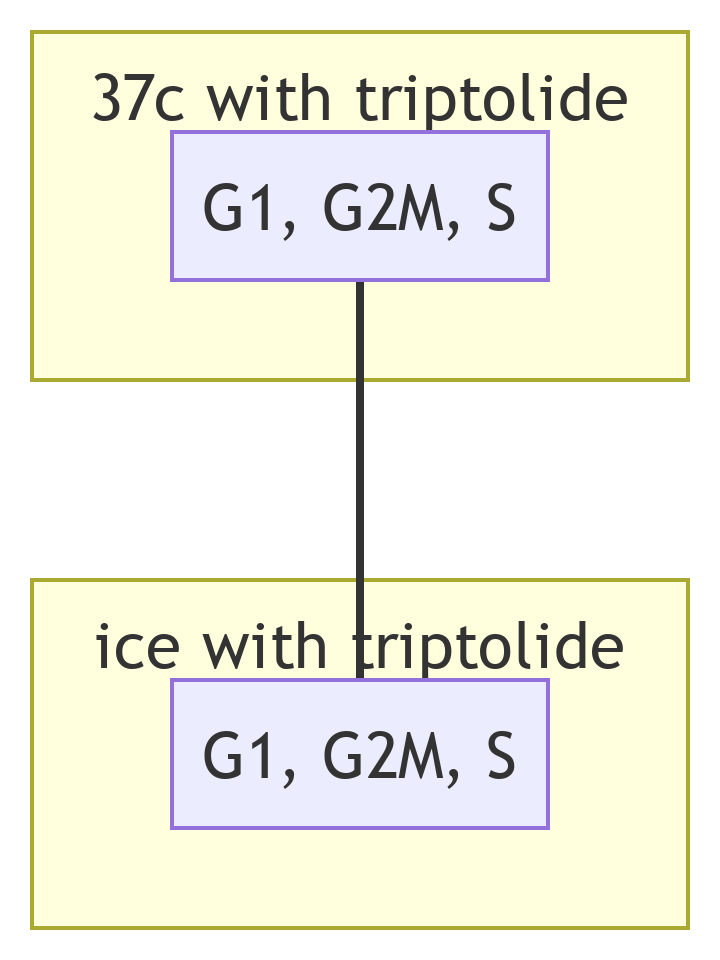

Supplement: Supplementary file 7 — The code for the bioinformatics analysis. [file 43587_2023_558_MOESM7_ESM.tar › notebooks/plots.r_files/figure-markdown_strict/mermaid-figure-2.png]

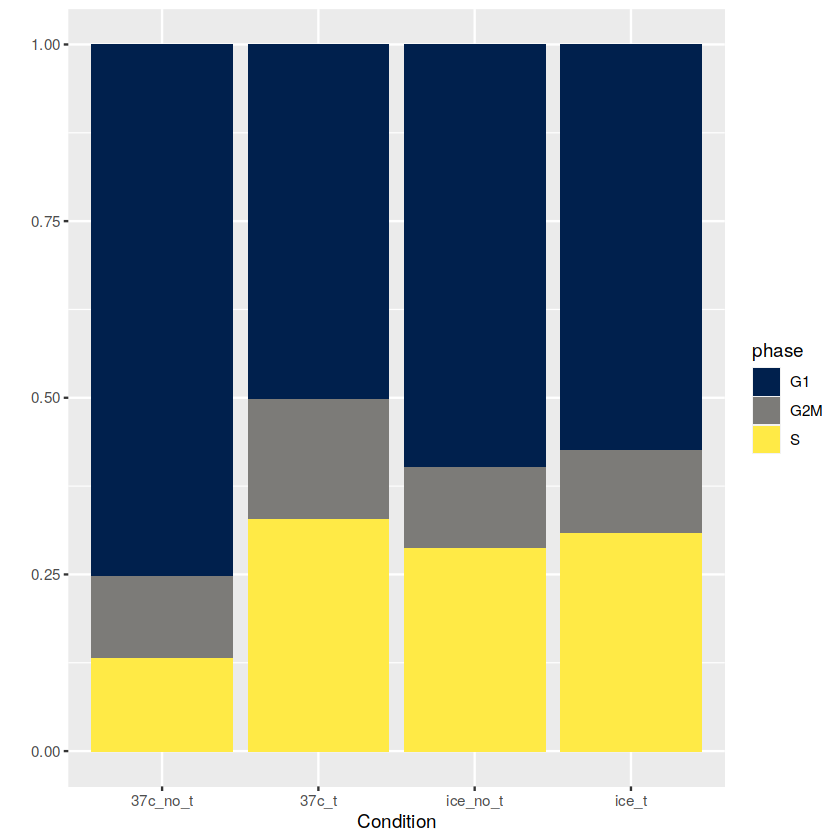

Supplement: Supplementary file 7 — The code for the bioinformatics analysis. [file 43587_2023_558_MOESM7_ESM.tar › notebooks/sc_qc.r_files/figure-markdown_strict/cell-14-output-1.png]

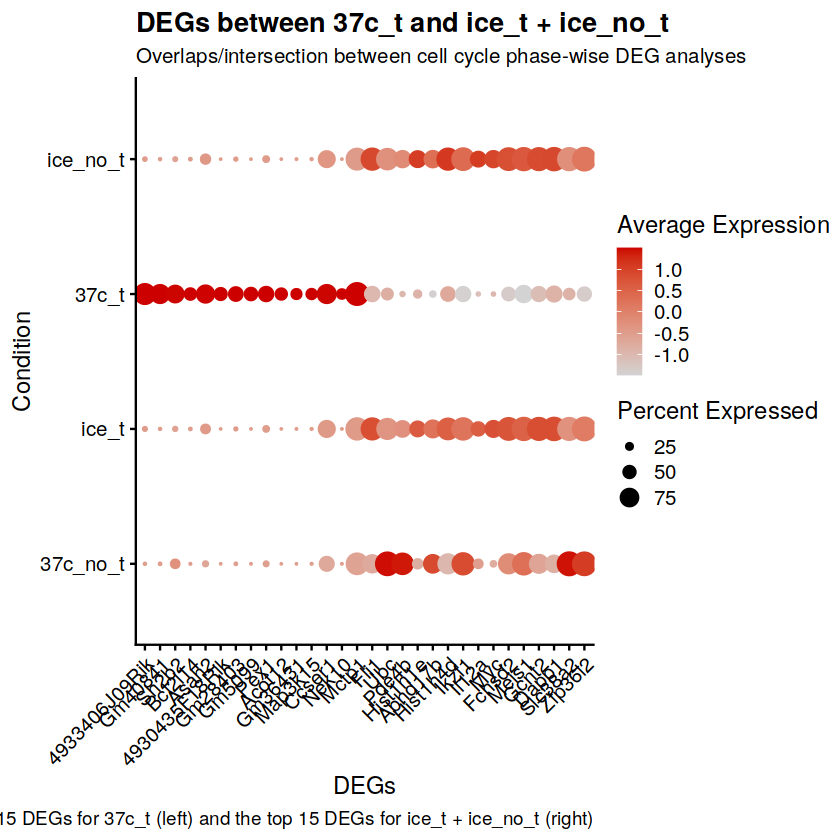

Supplement: Supplementary file 7 — The code for the bioinformatics analysis. [file 43587_2023_558_MOESM7_ESM.tar › notebooks/sc_qc.r_files/figure-markdown_strict/cell-45-output-2.png]

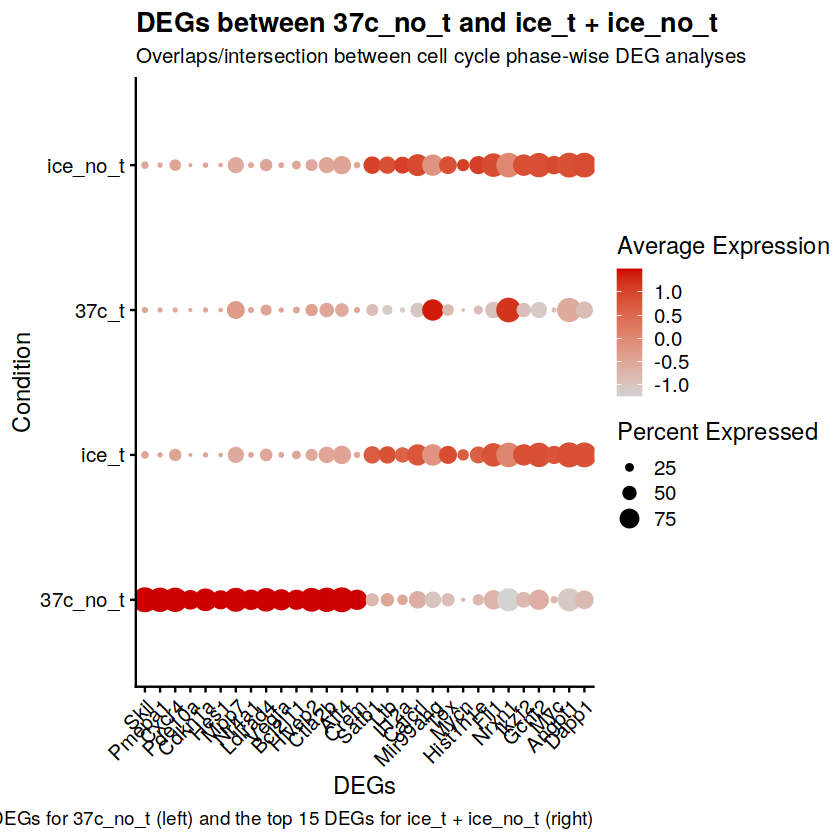

Supplement: Supplementary file 7 — The code for the bioinformatics analysis. [file 43587_2023_558_MOESM7_ESM.tar › notebooks/sc_qc.r_files/figure-markdown_strict/cell-47-output-4.png]

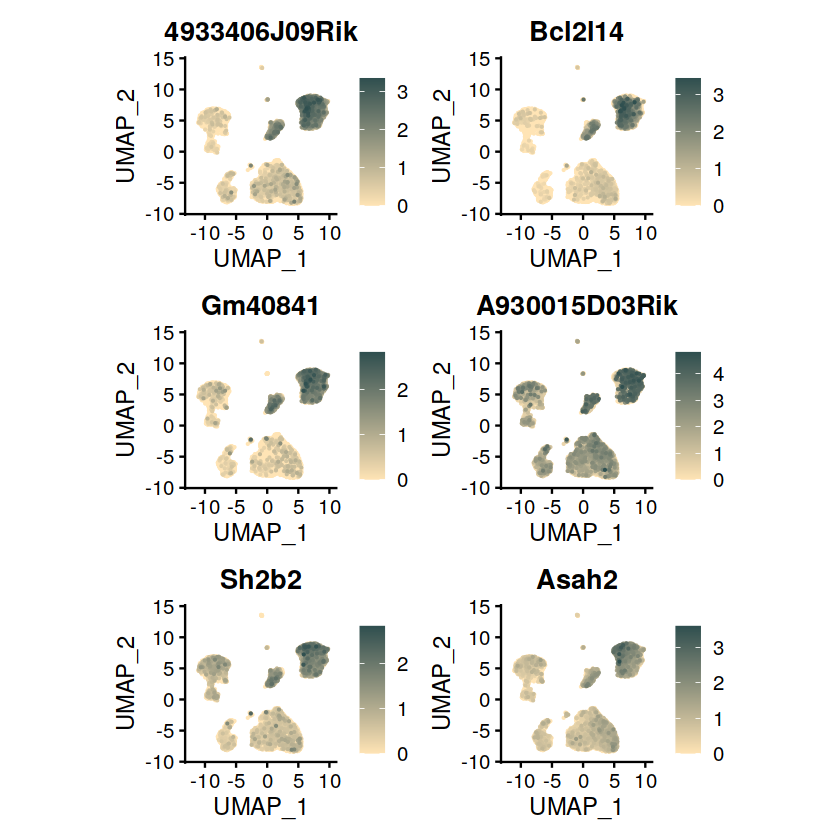

Supplement: Supplementary file 7 — The code for the bioinformatics analysis. [file 43587_2023_558_MOESM7_ESM.tar › notebooks/plots.r_files/figure-markdown_strict/fig-deg-new-output-1.png]

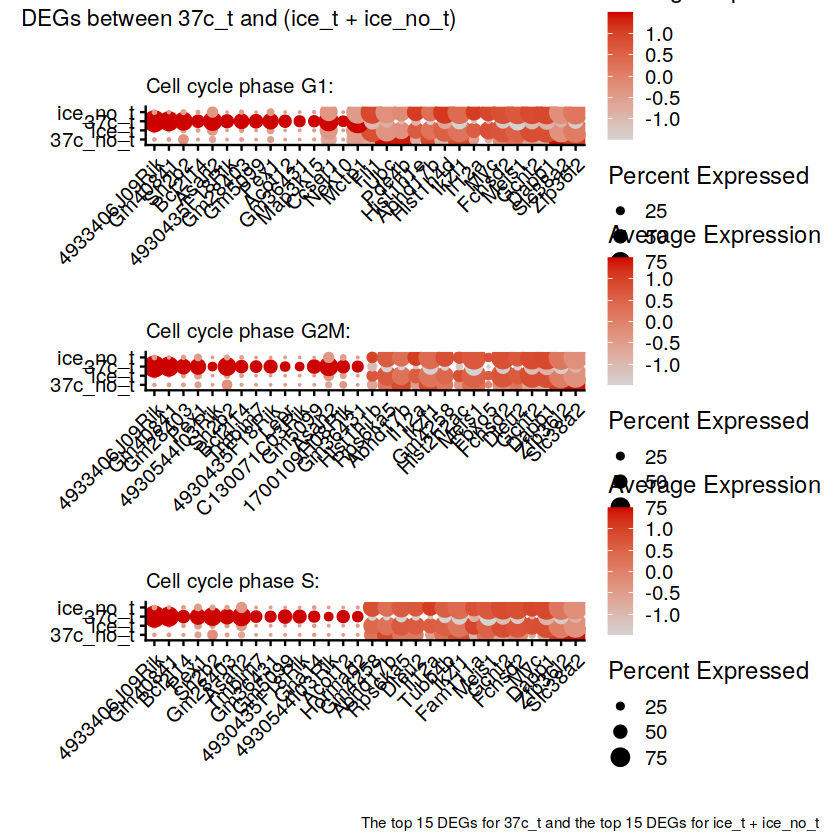

Supplement: Supplementary file 7 — The code for the bioinformatics analysis. [file 43587_2023_558_MOESM7_ESM.tar › notebooks/sc_qc.r_files/figure-markdown_strict/cell-39-output-2.png]

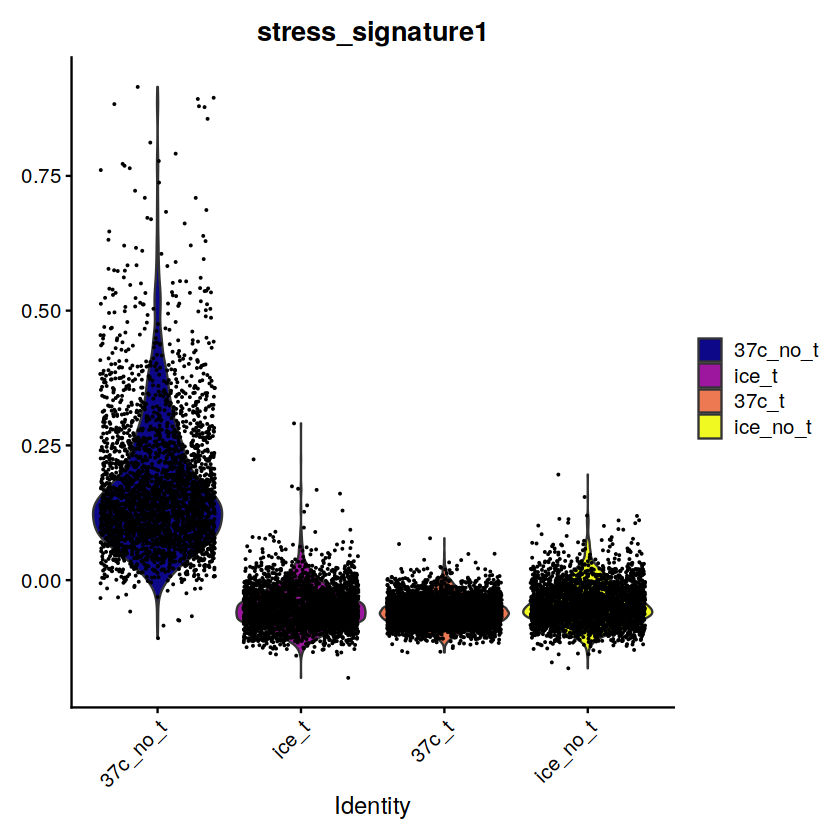

Supplement: Supplementary file 7 — The code for the bioinformatics analysis. [file 43587_2023_558_MOESM7_ESM.tar › notebooks/sc_qc.r_files/figure-markdown_strict/cell-55-output-7.png]

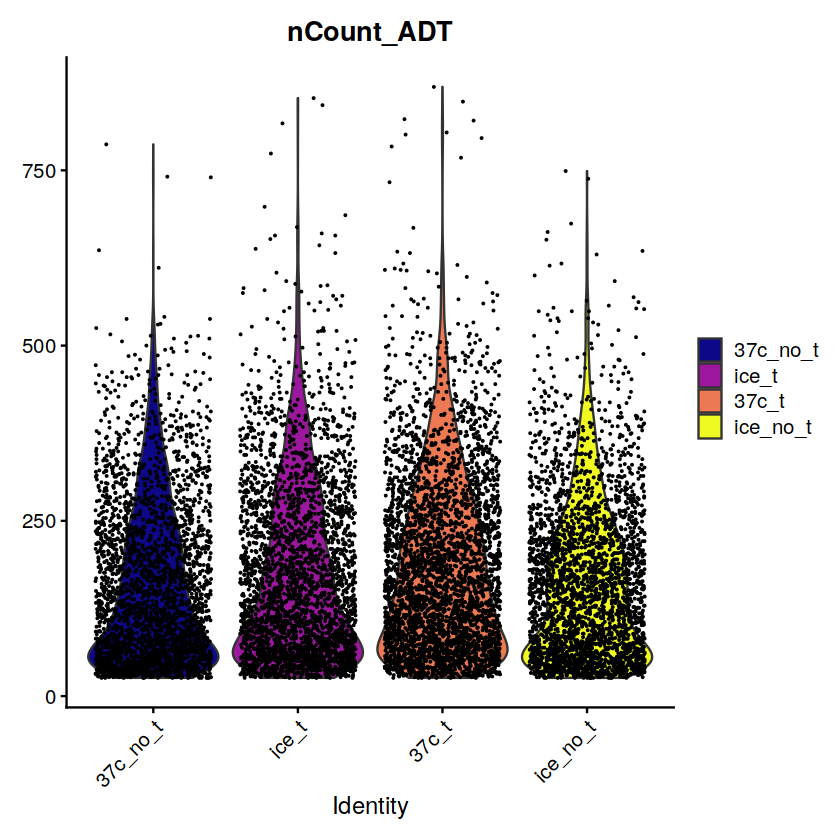

Supplement: Supplementary file 7 — The code for the bioinformatics analysis. [file 43587_2023_558_MOESM7_ESM.tar › notebooks/sc_qc.r_files/figure-markdown_strict/cell-55-output-3.png]

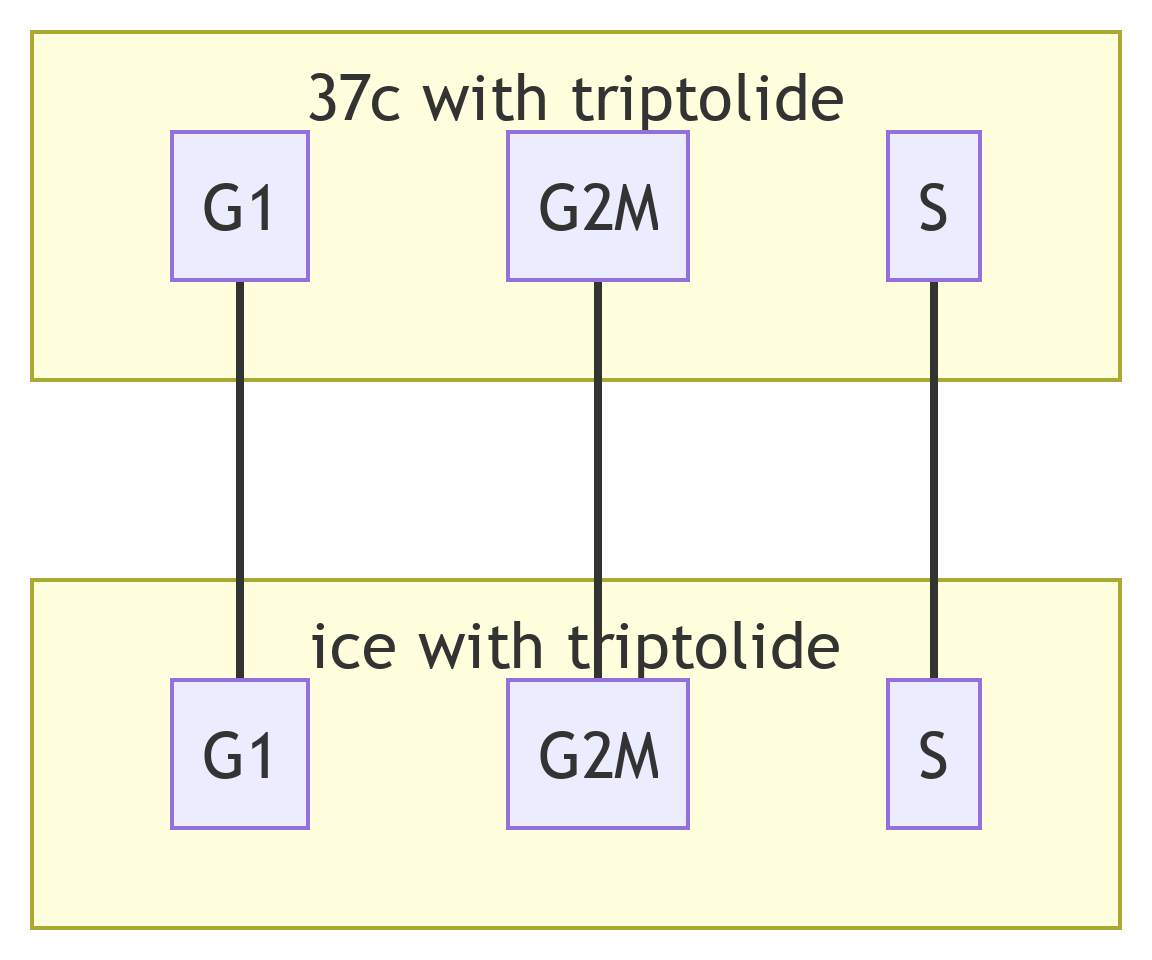

Supplement: Supplementary file 7 — The code for the bioinformatics analysis. [file 43587_2023_558_MOESM7_ESM.tar › notebooks/plots.r_files/figure-markdown_strict/mermaid-figure-1.png]

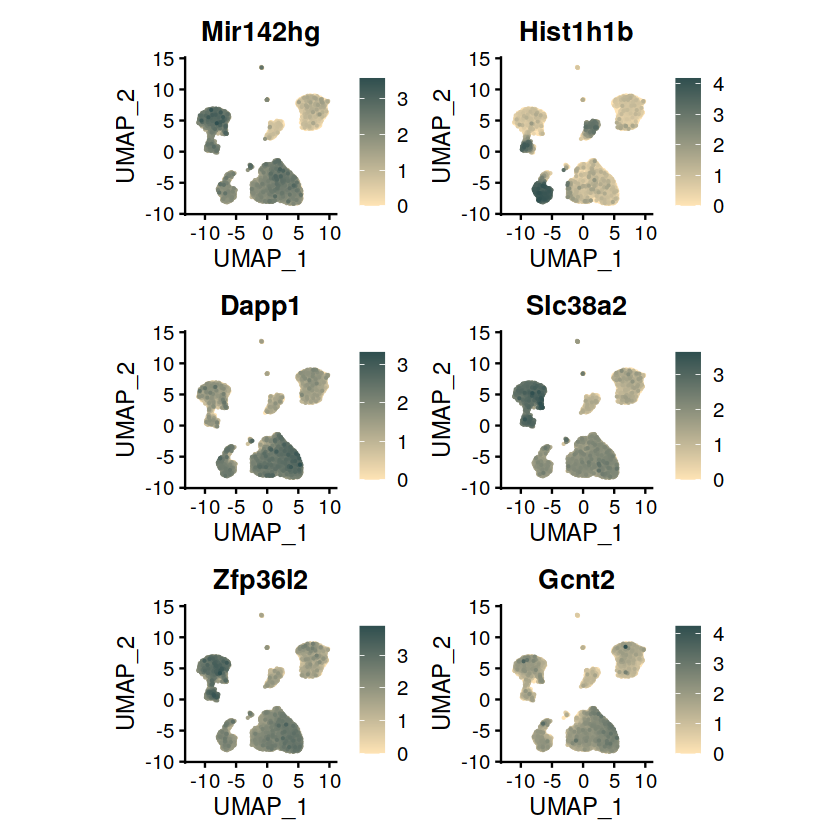

Supplement: Supplementary file 7 — The code for the bioinformatics analysis. [file 43587_2023_558_MOESM7_ESM.tar › notebooks/plots.r_files/figure-markdown_strict/fig-deg-old-g2m-output-1.png]

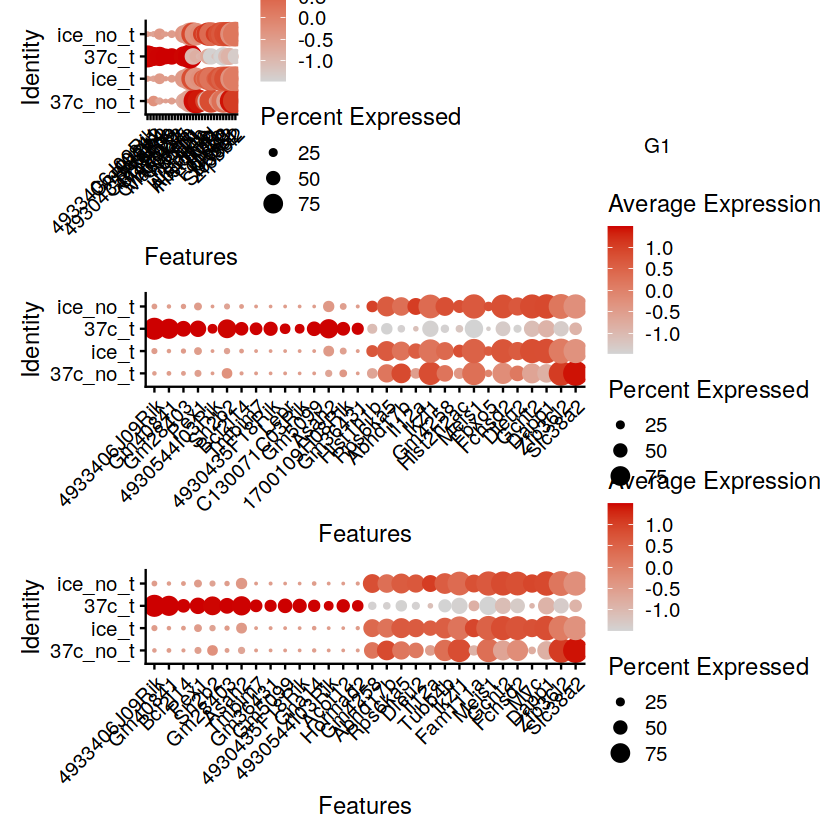

Supplement: Supplementary file 7 — The code for the bioinformatics analysis. [file 43587_2023_558_MOESM7_ESM.tar › notebooks/sc_qc.r_files/figure-markdown_strict/cell-36-output-1.png]

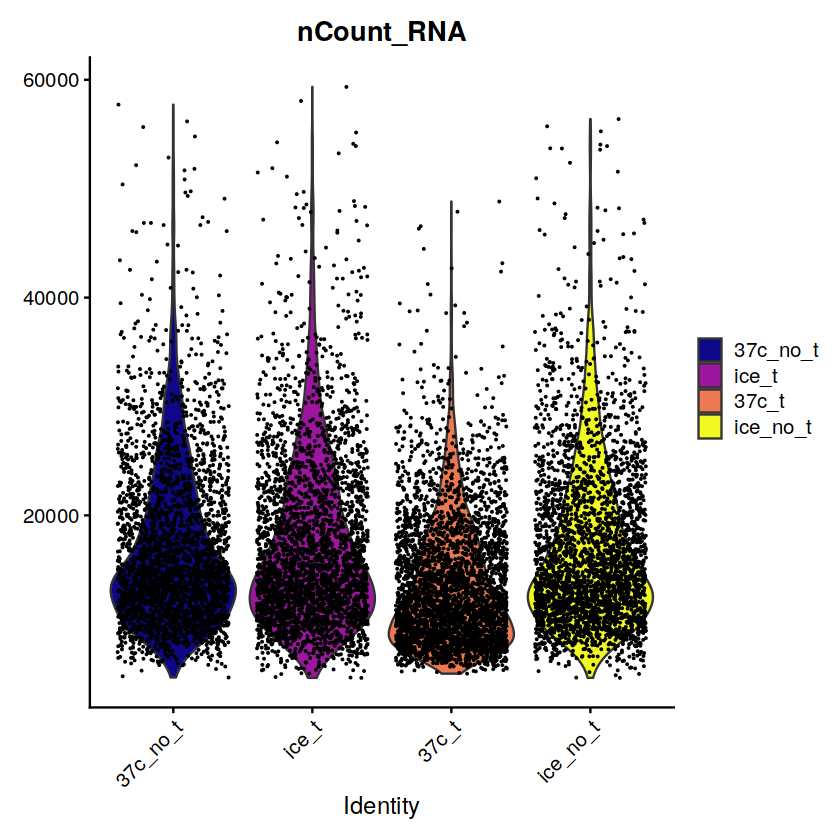

Supplement: Supplementary file 7 — The code for the bioinformatics analysis. [file 43587_2023_558_MOESM7_ESM.tar › notebooks/sc_qc.r_files/figure-markdown_strict/cell-51-output-1.png]

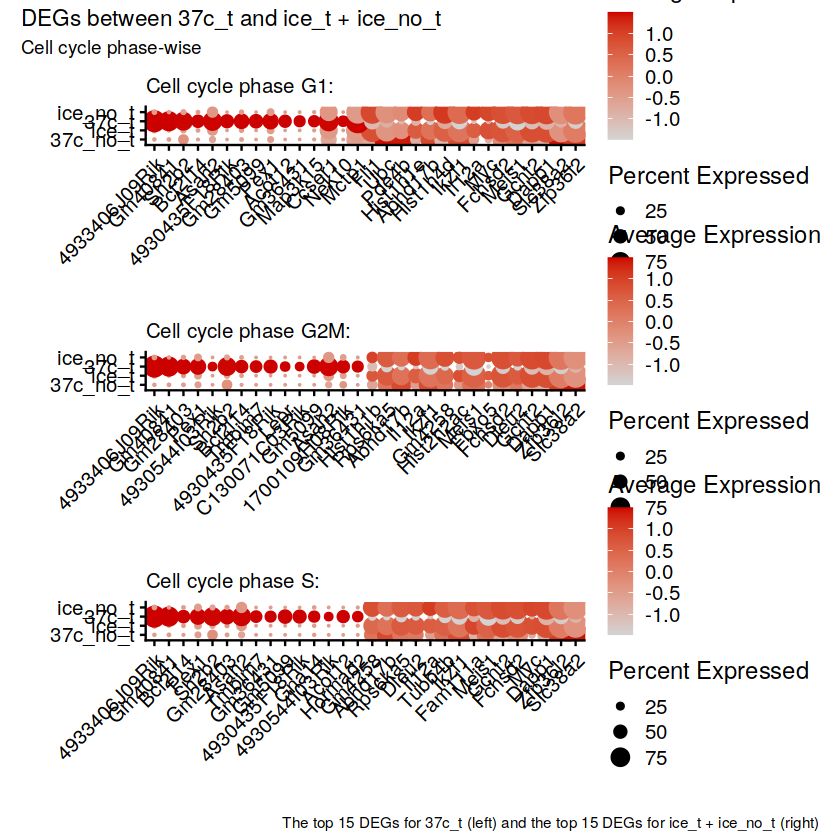

Supplement: Supplementary file 7 — The code for the bioinformatics analysis. [file 43587_2023_558_MOESM7_ESM.tar › notebooks/sc_qc.r_files/figure-markdown_strict/cell-41-output-2.png]

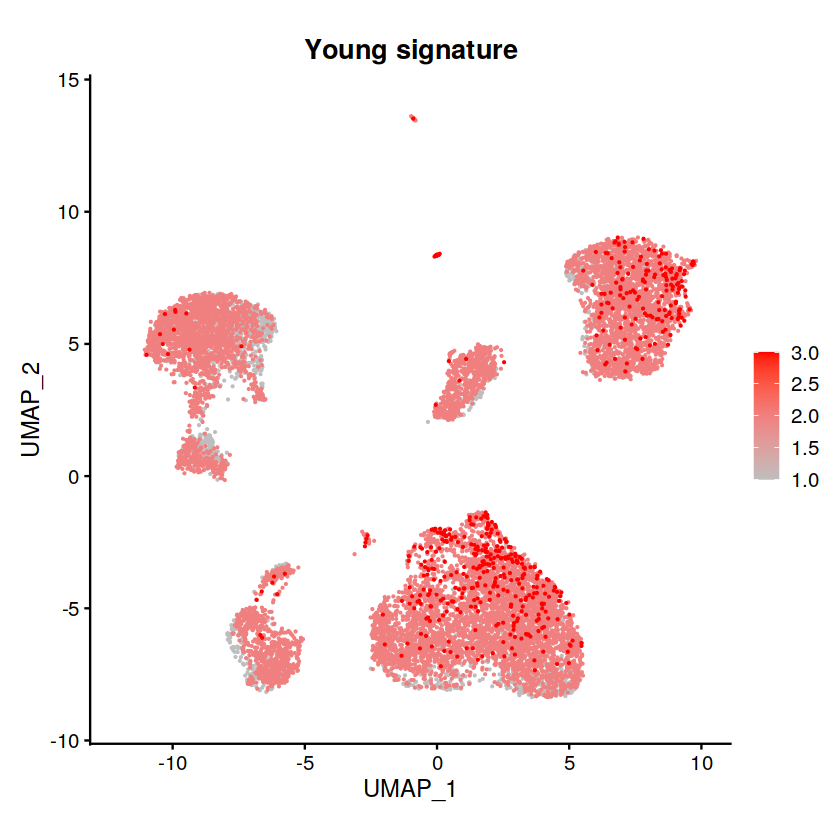

Supplement: Supplementary file 7 — The code for the bioinformatics analysis. [file 43587_2023_558_MOESM7_ESM.tar › notebooks/sc_qc.r_files/figure-markdown_strict/cell-66-output-2.png]

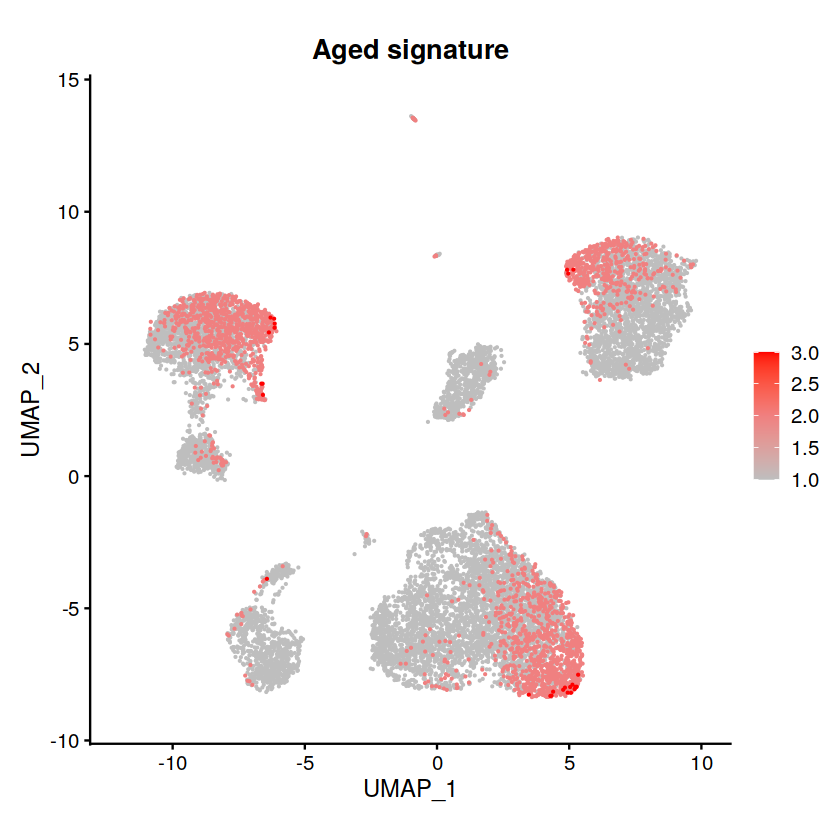

Supplement: Supplementary file 7 — The code for the bioinformatics analysis. [file 43587_2023_558_MOESM7_ESM.tar › notebooks/sc_qc.r_files/figure-markdown_strict/cell-66-output-3.png]

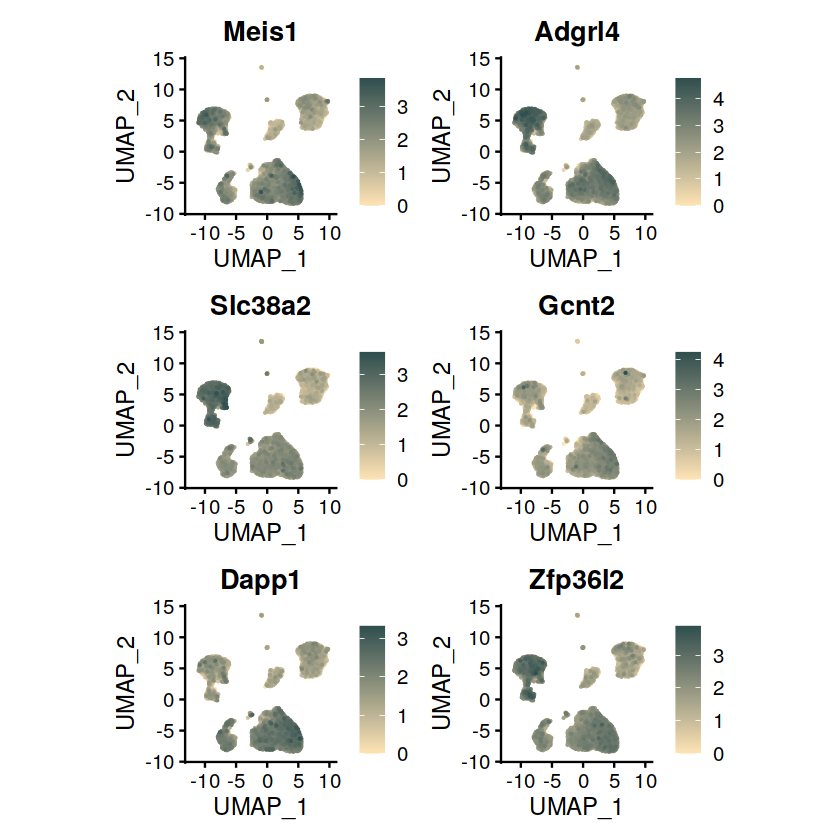

Supplement: Supplementary file 7 — The code for the bioinformatics analysis. [file 43587_2023_558_MOESM7_ESM.tar › notebooks/plots.r_files/figure-markdown_strict/fig-deg-new-output-2.png]

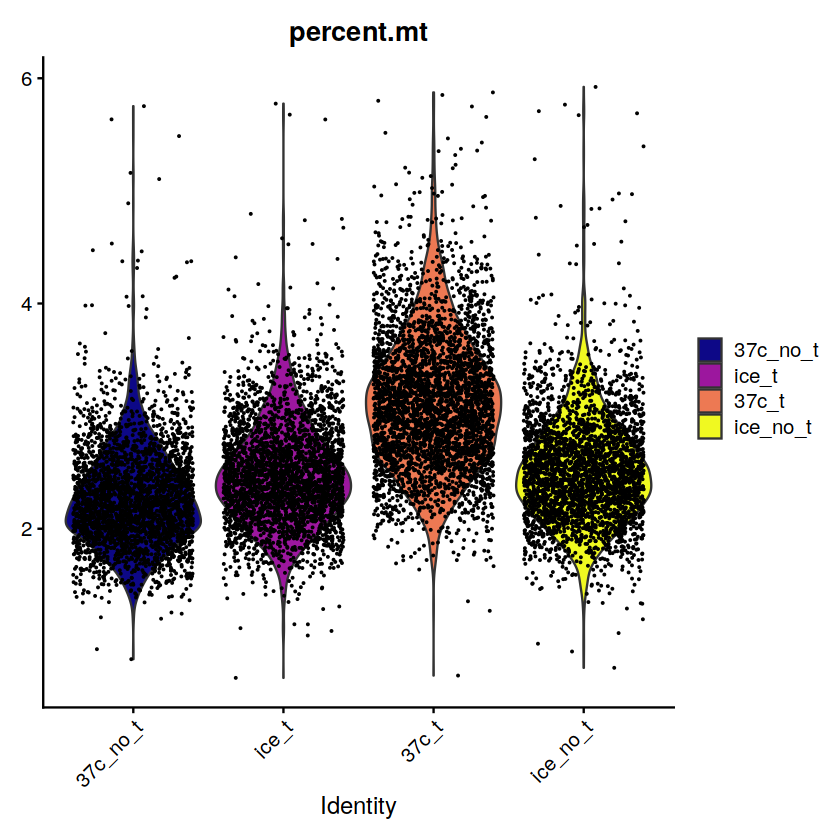

Supplement: Supplementary file 7 — The code for the bioinformatics analysis. [file 43587_2023_558_MOESM7_ESM.tar › notebooks/sc_qc.r_files/figure-markdown_strict/cell-55-output-6.png]

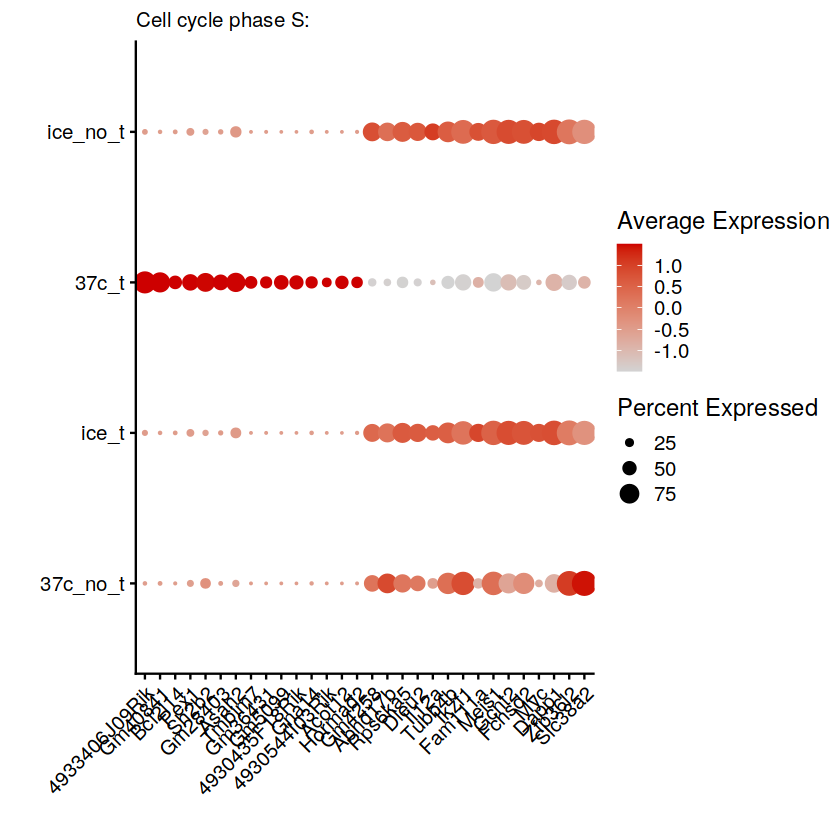

Supplement: Supplementary file 7 — The code for the bioinformatics analysis. [file 43587_2023_558_MOESM7_ESM.tar › notebooks/sc_qc.r_files/figure-markdown_strict/cell-38-output-2.png]

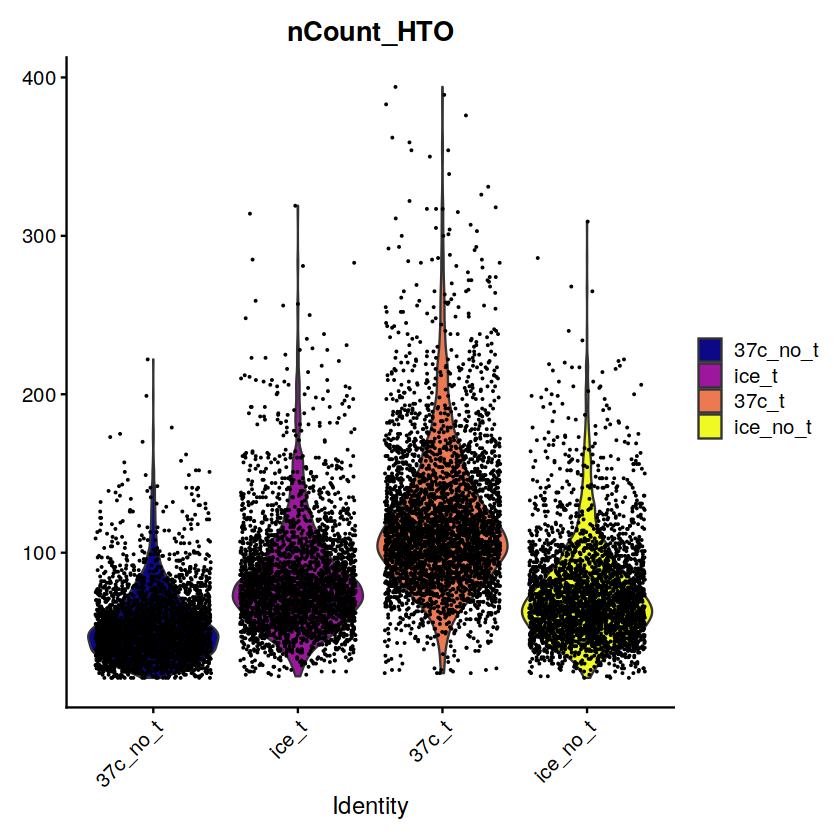

Supplement: Supplementary file 7 — The code for the bioinformatics analysis. [file 43587_2023_558_MOESM7_ESM.tar › notebooks/sc_qc.r_files/figure-markdown_strict/cell-55-output-4.png]

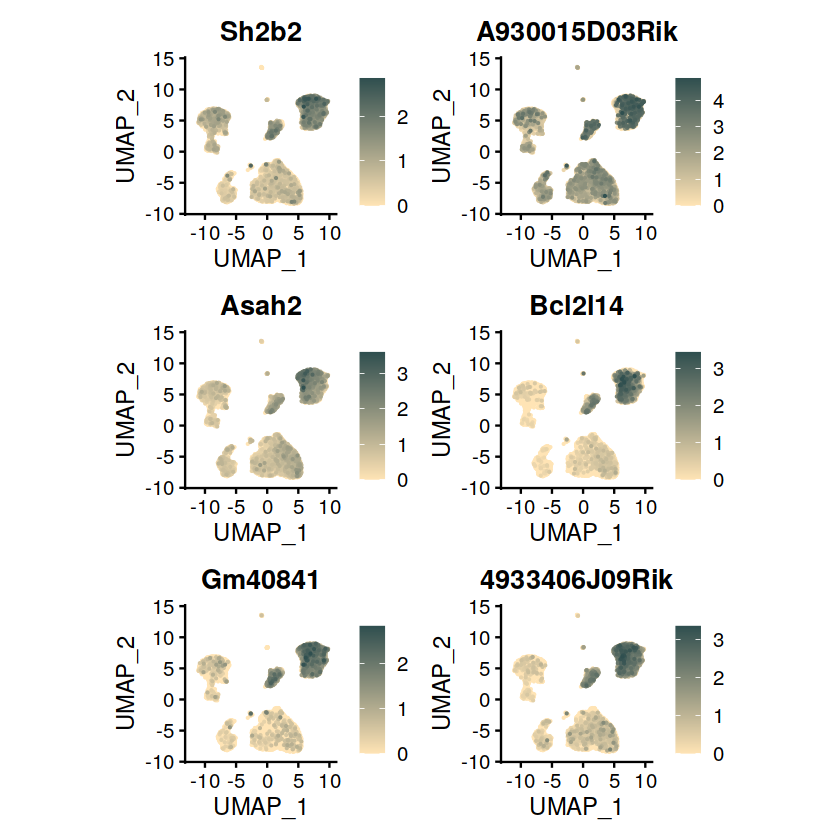

Supplement: Supplementary file 7 — The code for the bioinformatics analysis. [file 43587_2023_558_MOESM7_ESM.tar › notebooks/plots.r_files/figure-markdown_strict/fig-deg-old-g1-output-2.png]

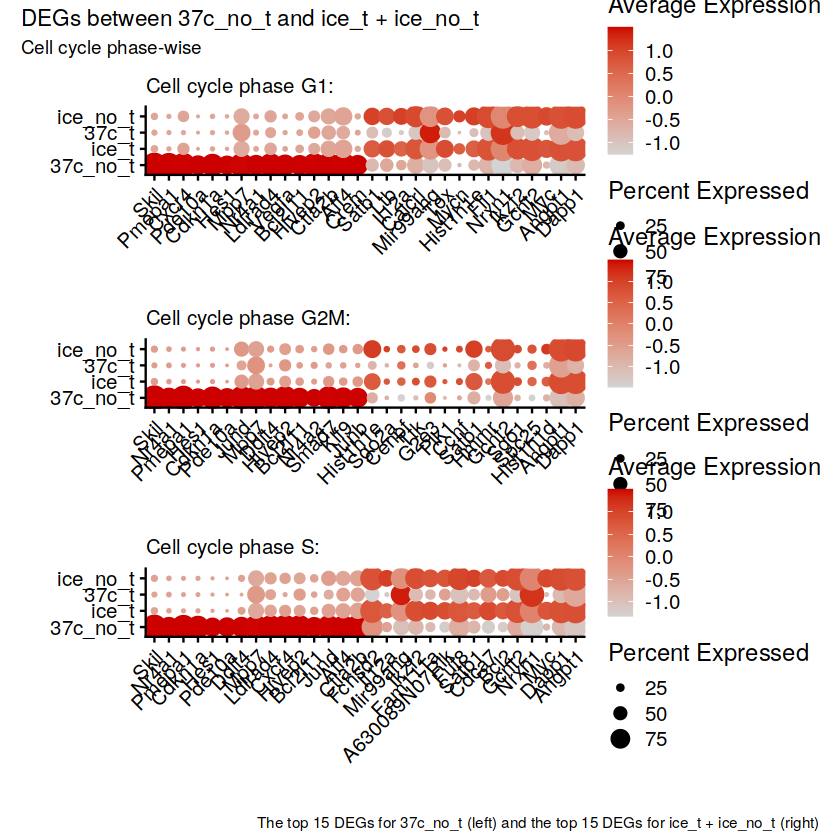

Supplement: Supplementary file 7 — The code for the bioinformatics analysis. [file 43587_2023_558_MOESM7_ESM.tar › notebooks/sc_qc.r_files/figure-markdown_strict/cell-43-output-2.png]

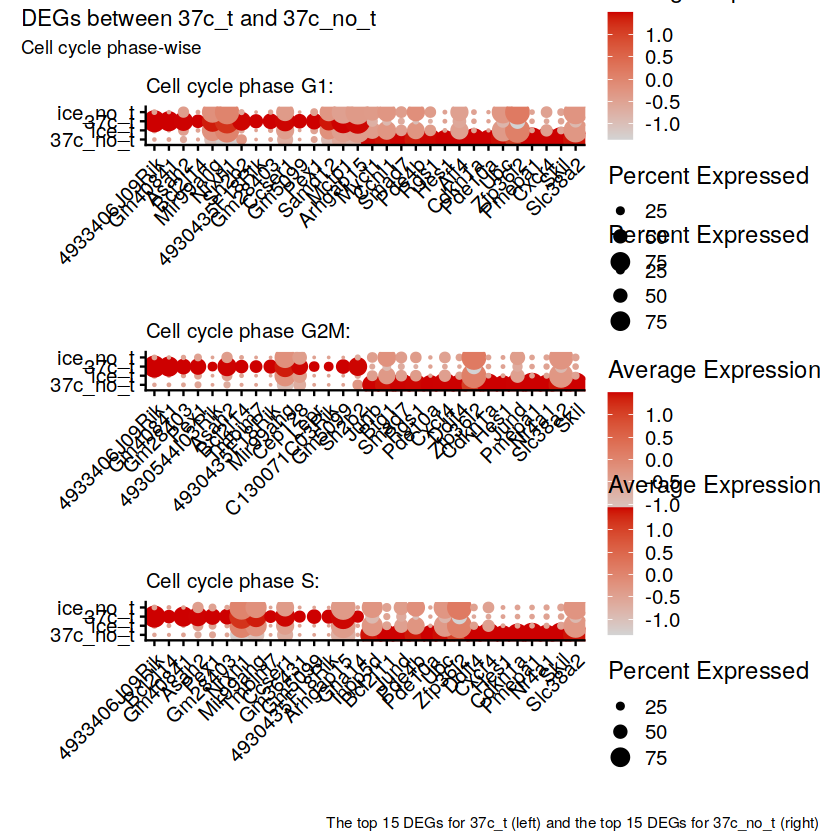

Supplement: Supplementary file 7 — The code for the bioinformatics analysis. [file 43587_2023_558_MOESM7_ESM.tar › notebooks/sc_qc.r_files/figure-markdown_strict/cell-44-output-2.png]

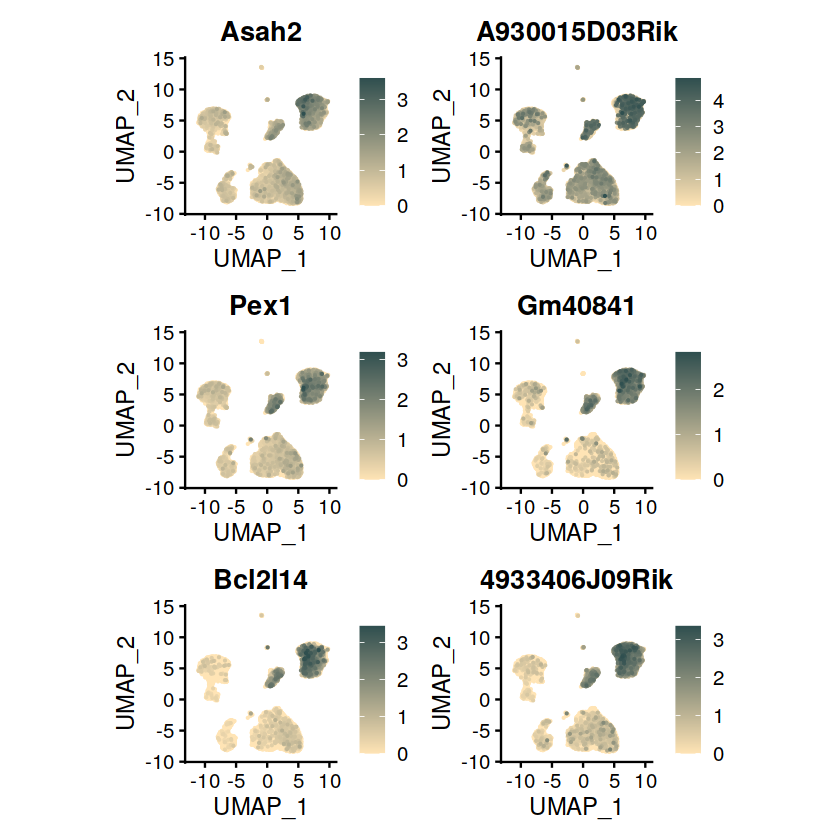

Supplement: Supplementary file 7 — The code for the bioinformatics analysis. [file 43587_2023_558_MOESM7_ESM.tar › notebooks/plots.r_files/figure-markdown_strict/fig-deg-old-s-output-2.png]

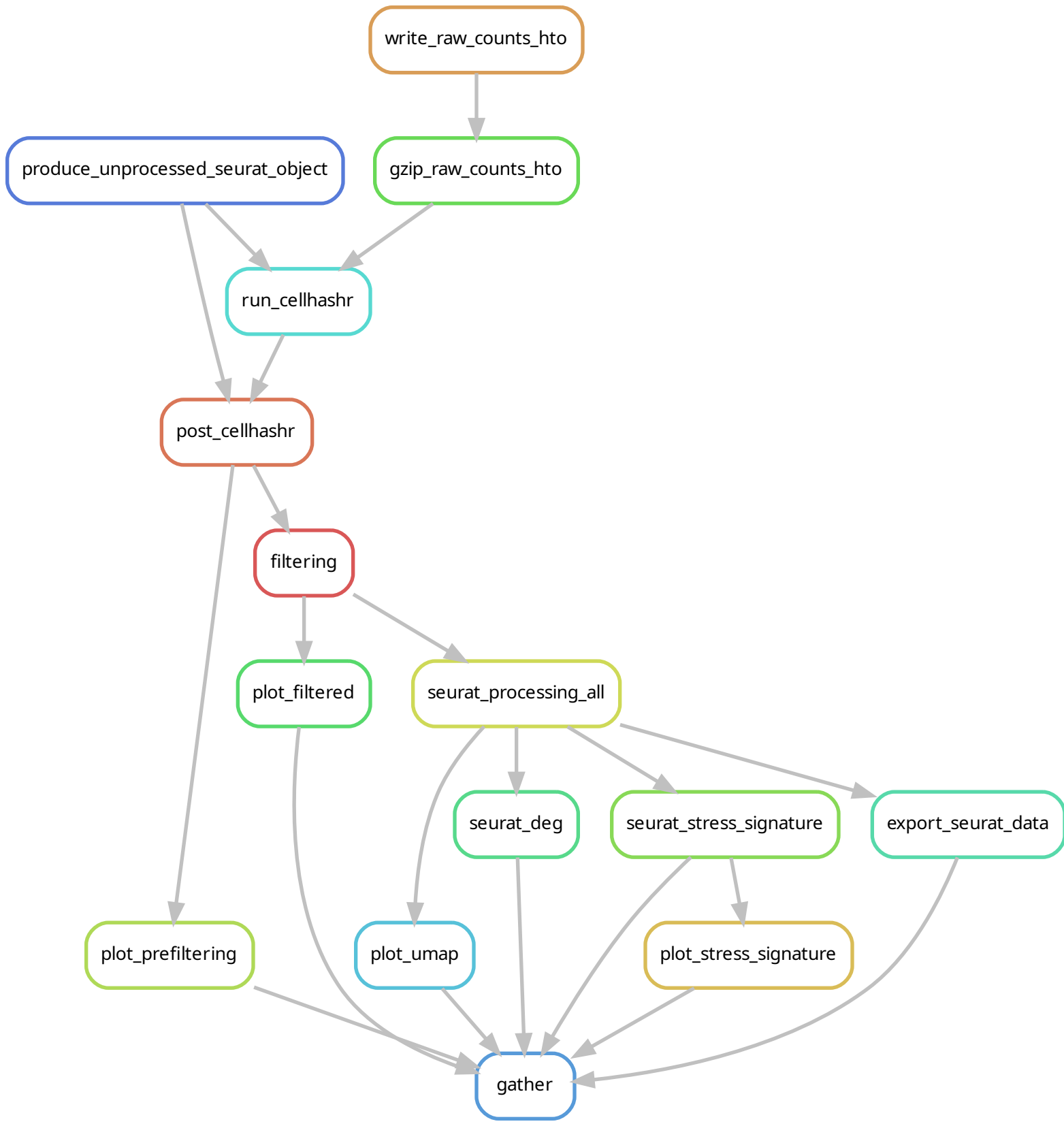

Supplement: Supplementary file 7 — The code for the bioinformatics analysis. [file 43587_2023_558_MOESM7_ESM.tar › smk_rulegraph.pdf]

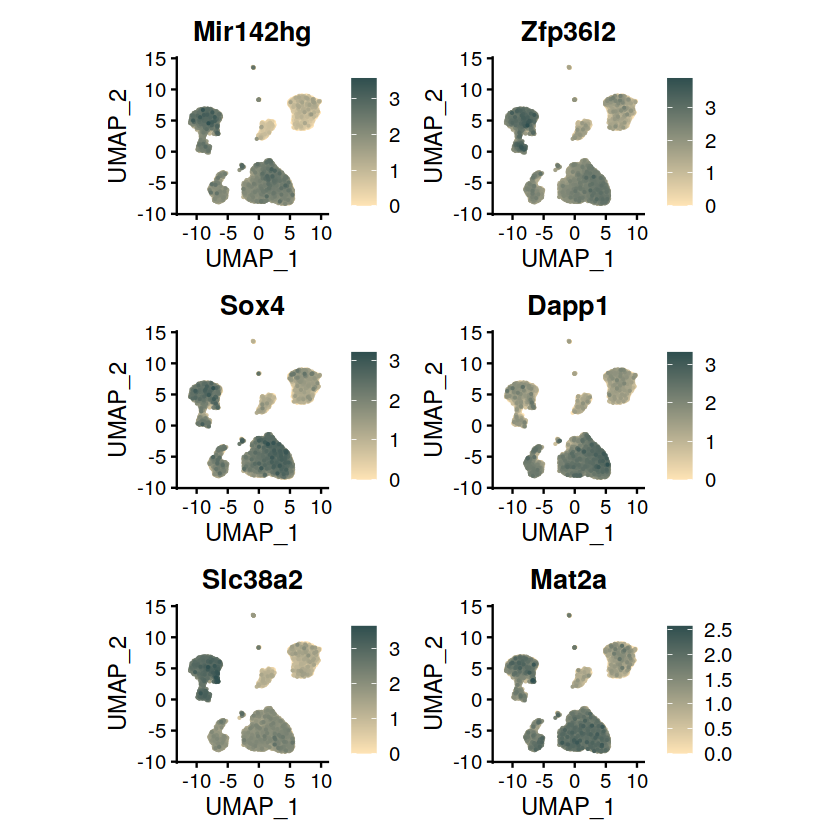

Supplement: Supplementary file 7 — The code for the bioinformatics analysis. [file 43587_2023_558_MOESM7_ESM.tar › notebooks/plots.r_files/figure-markdown_strict/fig-deg-old-s-output-1.png]

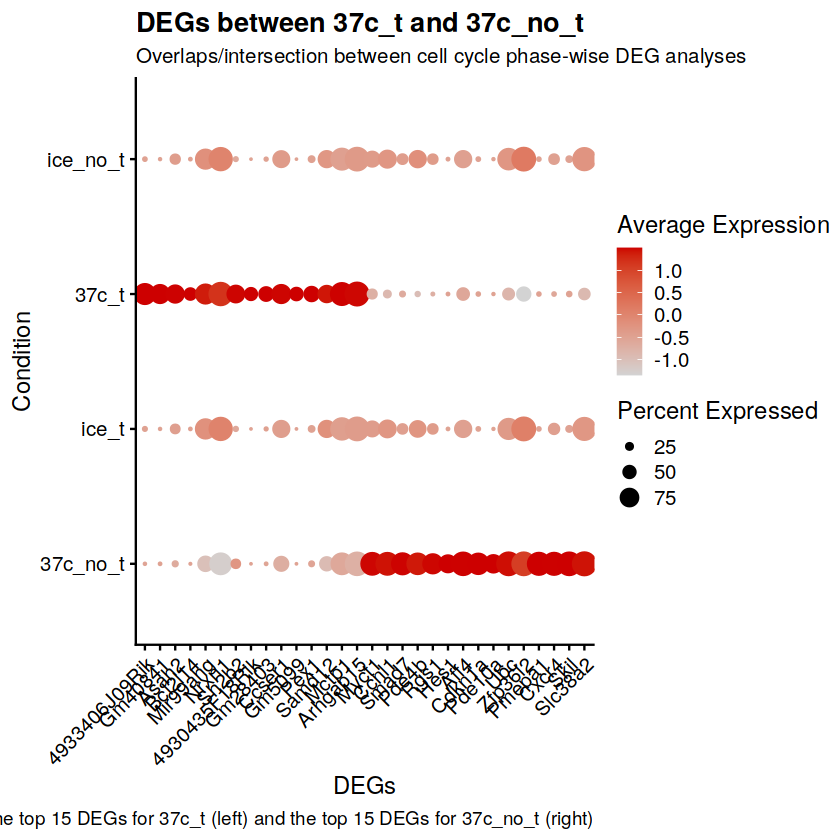

Supplement: Supplementary file 7 — The code for the bioinformatics analysis. [file 43587_2023_558_MOESM7_ESM.tar › notebooks/sc_qc.r_files/figure-markdown_strict/cell-47-output-3.png]

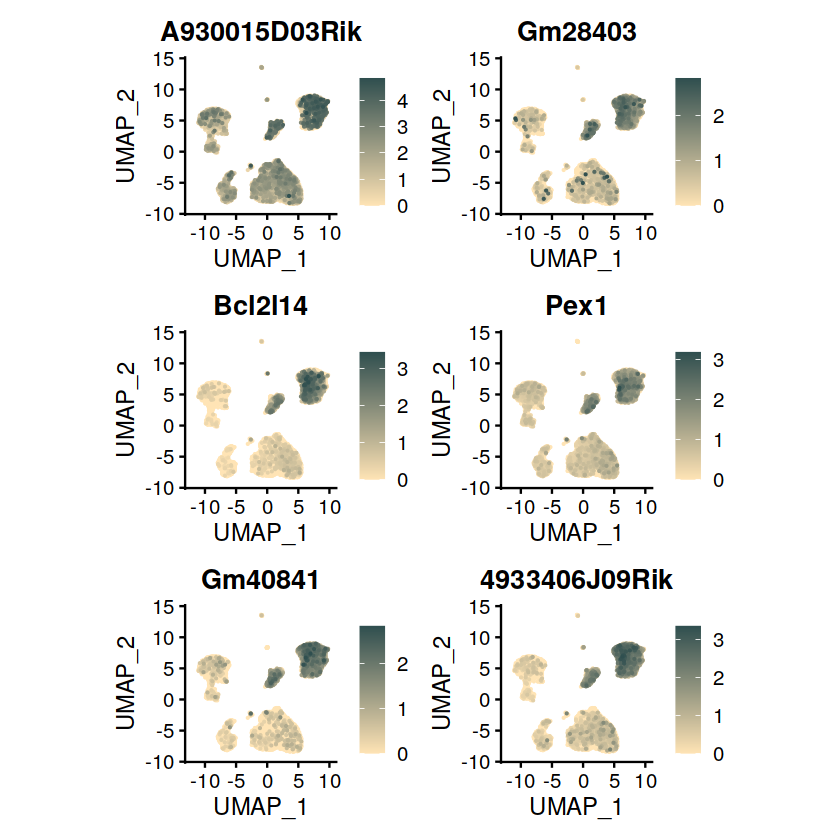

Supplement: Supplementary file 7 — The code for the bioinformatics analysis. [file 43587_2023_558_MOESM7_ESM.tar › notebooks/plots.r_files/figure-markdown_strict/fig-deg-old-g2m-output-2.png]

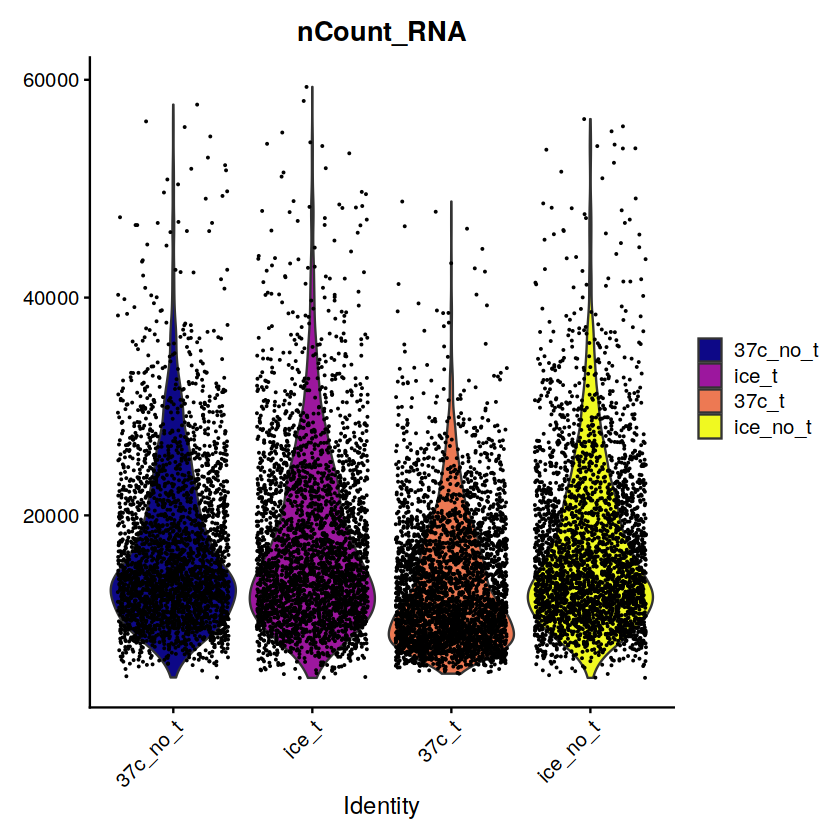

Supplement: Supplementary file 7 — The code for the bioinformatics analysis. [file 43587_2023_558_MOESM7_ESM.tar › notebooks/sc_qc.r_files/figure-markdown_strict/cell-55-output-1.png]

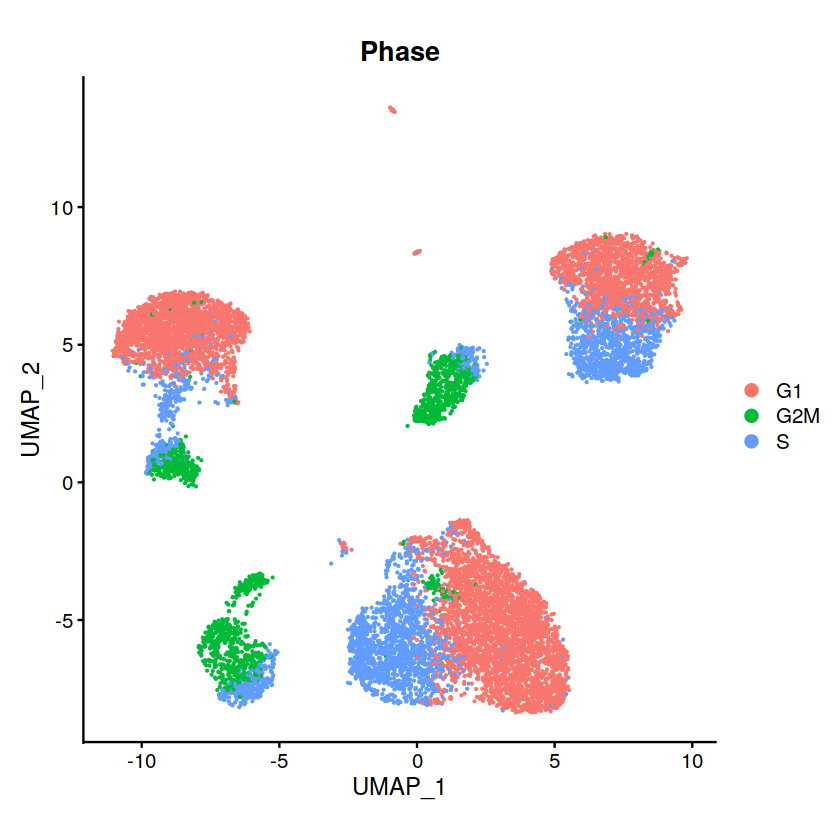

Supplement: Supplementary file 7 — The code for the bioinformatics analysis. [file 43587_2023_558_MOESM7_ESM.tar › notebooks/plots.r_files/figure-markdown_strict/fig-dimplots-output-2.png]

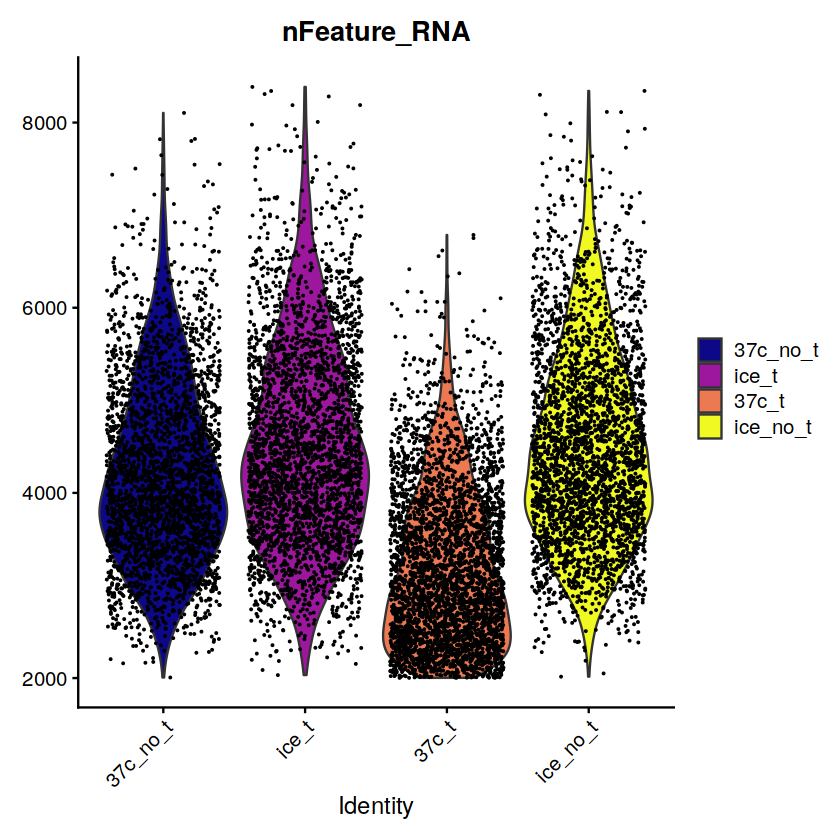

Supplement: Supplementary file 7 — The code for the bioinformatics analysis. [file 43587_2023_558_MOESM7_ESM.tar › notebooks/sc_qc.r_files/figure-markdown_strict/cell-55-output-2.png]
